# Structural dynamics of RNA polymerase II throughout the nucleotide addition cycle

**DOI:** 10.1038/s41467-026-75257-5

**Published:** 2026-07-08

**Authors:** Gangshun Yi, Qingrong Li, Hannah Holmberg, Shisheng Li, Daniel K. Clare, Dong Wang, Peijun Zhang

**Affiliations:** 1 Division of Structural Biology, Nuffield Department of Medicine, University of Oxford, Oxford, UK; 2 Diamond Light Source, Harwell Science and Innovation Campus, Didcot, UK; 3 Department of Pharmaceutical Sciences, Skaggs School of Pharmacy and Pharmaceutical Sciences, University of California San Diego, La Jolla, CA USA; 4 Department of Comparative Biomedical Sciences, School of Veterinary Medicine, Louisiana State University, Baton Rouge, LA USA; 5 Department of Molecular and Cellular Medicine, School of Medicine, University of California San Diego, La Jolla, CA USA; 6 Department of Chemistry and Biochemistry, University of California San Diego, La Jolla, CA USA; 7 Chinese Academy of Medical Sciences Oxford Institute, Nuffield Department of Medicine, University of Oxford, Oxford, UK

**Keywords:** Cryoelectron microscopy, Transcription, Biological physics

## Abstract

RNA polymerase II (RNAPII) drives gene expression through iterative nucleotide addition cycles (NACs) comprising translocation, substrate binding, and catalysis. The lack of pre-catalysis and post-catalysis intermediates has precluded a complete mechanistic understanding of the NAC. Here we present 31 Cryo-electron Microscopy structures (with 43 maps) capturing distinct stages of *Saccharomyces cerevisiae* RNAPII elongation complex (EC) NAC, including previously intractable transition intermediates. We establish a continuous spectrum of RNAPII EC structural dynamics during the NAC, which can be divided into two coordinated phases: a substrate-induced EC tightening phase and a post-catalysis EC relaxation phase. For the substrate-induced EC tightening phase, the substrate binding initiates allosteric conformational changes across the entire RNAPII EC, including Trigger Loop folding, funnel closure, clamp closure, transcription bubble ordering, and precise alignment of the RNA 3′-end with substrate to form a catalysis-competent configuration. For the post-catalysis EC relaxation phase, we capture the long-sought, short-lived post-catalysis product state and identify a series of intermediates that reveal a reverse conformational transition that facilitates rapid translocation. Together, our findings define a comprehensive structural and dynamic framework for RNAPII NAC, yielding a “molecular movie” of RNAPII in action and revealing a fundamental principle by which the enzyme balances speed and fidelity through coordinated conformational dynamics.

## Abstract (web summary)

RNA polymerase II catalyses transcription through nucleotide addition cycles, but key structural intermediates were unresolved. Here, the authors present cryo‑EM snapshots capturing the elongation cycle, revealing coordinated conformational dynamics that balance catalytic efficiency and fidelity.

## Introduction

**Fig. 1:**
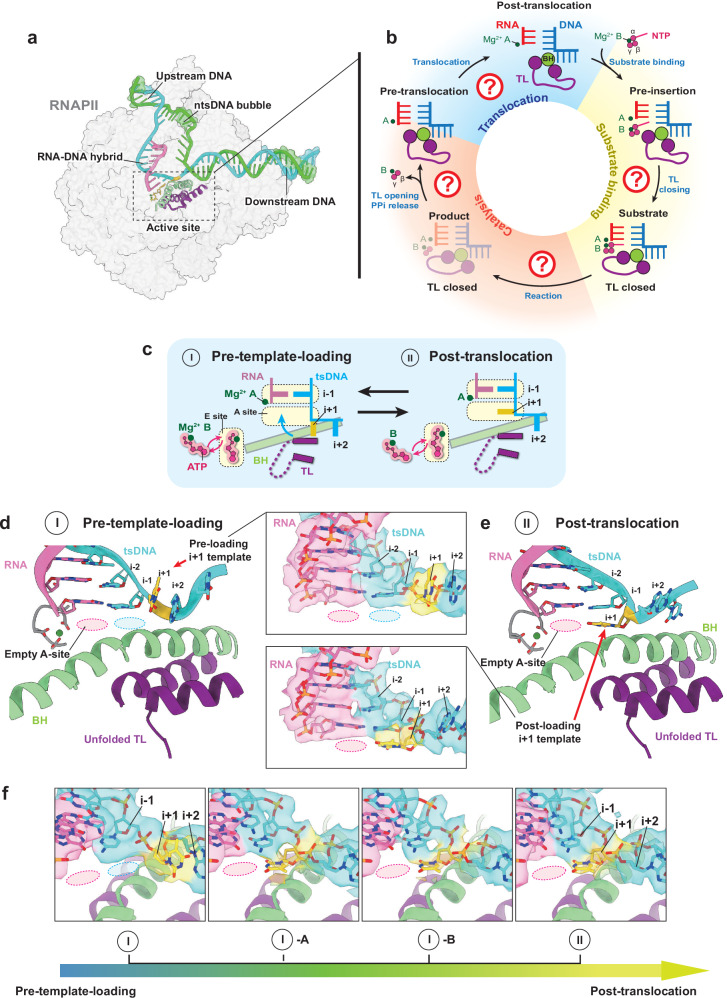
A pre-template-loading state reveals early events in the nucleotide addition cycle. **a** Representative structure of the RNAPII EC. The structure is shown from Sample 2 (frame 20, state V) and is presented as a representative conformation of the RNAPII EC. RNAPII is colored in silver, with tsDNA (cyan), ntsDNA (lime), and RNA (hot pink). The active center is highlighted by a dashed box. Key active center elements, including the BH (light green) and TL (purple), are shown. The substrate ATP is depicted in gold. Abbreviations, residue ranges, and color codes for RNAPII EC elements are summarized in Supplementary Table 6. **b** Schematic illustrating sequential conformational changes within the active center of RNAPII during the nucleotide addition cycle. Four of the five states have been resolved previously by cryo-EM or X-ray crystallography (solid colors). The previously unresolved product state is depicted as a transparent cartoon. The nucleotide addition cycle is categorized into three sections highlighted by distinct background colors: translocation (blue), substrate binding (yellow green), and catalysis (orange). Unknown mechanisms of conformational transition are indicated as question marks. Domain-specific color is consistent with (**a**). **c** Cartoon representation detailing the pre-template-loading state (state I) and its transition to the post-translocation state (state II), observed in both the RNAPII EC and RNAPII EC substrate-binding samples. **d**,**e** Cryo-EM structures of RNAPII EC in the pre-template-loading state (state I, **d**) and post-translocation state (state II, **e**) derived from the RNAPII EC substrate-free. Insets highlight cryo-EM density corresponding to the loading of the i + 1 template nucleotide (contoured at 3-σ). Coloring of RNAPII EC elements matches the scheme depicted in (**c**). **f** Cryo-EM maps displaying dynamic density changes during the translocation of the i + 1 template nucleotide (contoured at 3-σ).

RNA polymerase II (RNAPII) is a central enzyme for gene transcription. During transcription, RNAPII selects the correct substrate for reaction and undergoes significant conformational changes in an iterative nucleotide addition cycle (NAC)^1–7^. The NAC can be divided into three major stages: translocation, substrate binding, and catalysis (Fig. 1a, b)^3,4^. During translocation^8^, RNAPII moves one base pair forward from the pre-translocation state^9^ to the post-translocation state^10,11^, in which the template base is loaded at i + 1 position, and the active site is available for substrate binding^11,12^. During substrate binding, RNAPII selects and binds the cognate substrate at the addition site (A-site) to form a Watson-Crick base pair with the template base at i + 1 position^12,13^. Previous research has shown that the trigger loop (TL) (Rpb1 residues Met1063–Val1107), a conserved structural motif, switches from an open to a closed state upon correct substrate binding, which is essential for RNAPII substrate selection and subsequent catalysis^12–15^. During catalysis, a phosphodiester bond forms between the incoming substrate and the 3′-end of RNA. Following bond formation, the TL is thought to transition from a closed to an open state, releasing byproduct pyrophosphate from the active site and returning the complex to the pre-translocation state, ready for the next cycle.

Despite significant progress over the past 25 years^2,5–7^, our understanding of the structural dynamics of the NAC remains limited, relying largely on four static snapshots of the translocation and substrate-binding steps (Fig. 1b)^3,4^. The critical process of catalysis, one of the central stages of the NAC, is not understood due to the absence of a high-resolution product structure upon catalysis (Fig. 1b, shaded orange). This product state is short-lived and challenging to capture using canonical structural approaches. Furthermore, the molecular basis of dynamic transitions between these states of the NAC remains elusive. Long-range allosteric conformational changes triggered by substrate binding and catalysis, beyond the TL opening and closing, are poorly understood. Several fundamental questions remain unanswered: Is template loading synchronized with upstream DNA/RNA movement during translocation? How does the TL fold to a closed conformation upon substrate binding and then unfold after catalysis? What is the temporal relationship between pyrophosphate release and TL opening? Does substrate binding lead to allosteric conformational changes in structural motifs beyond the TL, and how are these motions communicated?

Here we report cryo-EM structures capturing distinct stages of the *S. cerevisiae* RNAPII NAC, including previously intractable intermediates, and reveal dynamic conformational transitions across all stages of the cycle. In translocation, we identified a previously uncharacterized pre-template-loading state. In substrate binding, we resolved multiple previously unobserved intermediates that trace a near-continuous trajectory of TL folding, from fully open to partially folded to fully folded conformations. Substrate binding initiates long-range allosteric conformational changes across the entire RNAPII elongation complex (EC), which we refer to as substrate-induced EC tightening. These include TL folding, funnel closure, clamp closure, transcription bubble ordering, and precise alignment of the RNA 3′-end with the incoming substrate to form a catalysis-ready configuration. In catalysis, we captured the long-sought, short-lived post-catalysis product state and identified a series of intermediates that reveal a reverse conformational transition, which we termed post-catalysis EC relaxation, as RNAPII resets to a relaxed, translocation-ready state. Taken together, our results establish a coherent and comprehensive structural dynamic framework for the complete NAC, yielding a molecular movie of RNA polymerase II in action and revealing mechanistic insights that resolve long-standing questions in transcription.

## Results

To achieve a comprehensive understanding of the RNAPII EC NAC, we designed four cryo-EM samples to dissect the key steps of the *S. cerevisiae* RNAPII elongation cycle: a substrate-free RNAPII EC containing a 3′-deoxy (3′-dOH) 9-mer RNA to capture the different translocation states (Sample 1) (Supplementary Fig. 1, Supplementary Table 1); a RNAPII EC containing a 3′-dOH 9-mer RNA and ATP substrate to probe substrate binding states (Sample 2) (Supplementary Fig. 2, Supplementary Tables 2–3); a RNAPII EC with a regular 9-mer RNA primer and NTP substrates to enable completion of full NAC cycles (Sample 3) (Supplementary Figs. 3–4, Supplementary Table 4); and a RNAPII EC containing a long 3′-dOH RNA (19-mer) and ATP substrate to mimic a mature EC (Sample 4) (Supplementary Fig. 5, Supplementary Table 5). We employed monodispersed single-particle streptavidin (mspSA) affinity grids for cryo-EM sample preparation, which effectively mitigated air-water interface effects and reduced RNAPII orientation bias, thereby markedly enhancing resolution^16^. In both Samples 2 and 4, binding of ATP primes the EC for catalysis, but the absence of a 3′ hydroxyl group prevents nucleotide addition. For Sample 3, to sustain continuous NAC turnover and capture transient intermediates directly on cryo-EM grids, we employed a substrate-gradient affinity grid (SGAG) strategy (see Methods and Supplementary Figs. 3 and 4 for details). From these samples, we reconstructed 43 cryo-EM maps of RNAPII EC intermediates at resolutions ranging from 2.5 to 4.3 Å and built 31 corresponding atomic models (Supplementary Tables 1–5). Abbreviations, residue ranges, and color codes for RNAPII EC elements used in this study are listed in Supplementary Table 6. Collectively, these structures provide a comprehensive view of RNAPII structural dynamics, revealing conformational changes triggered by substrate binding and catalysis during the NAC (Summarized in Supplementary Movie 1).

### Template loading intermediates reveal unsynchronized RNAPII translocation

Previous studies have shown that multi-subunit RNAPs (including RNAPII) can spontaneously oscillate among translocation states in the absence of NTPs in vitro^17–24^. In addition to the previously characterized post-translocation state^10^, we captured several previously unobserved translocation intermediates in the substrate-free sample (Sample 1). One partial translocation intermediate, termed the pre-template-loading state (State I), features an upstream-shifted RNA-DNA hybrid, while the i + 1 template base has not yet crossed the bridge helix (Rpb1 residues Pro810–Ile848) or fully loaded into the active site (Fig. 1c, d). The position of the i + 1 base in this pre-template-loading state differs markedly from its canonical location in the post-translocation state^10^ (State II, Fig. 1b–d). We also identified two additional intermediates, States I-A and I-B, which capture progressive density shifts of the i + 1 nucleotide as it transitions from State I to the post-translocation conformation (State II) (Fig. 1e).

**Fig. 2:**
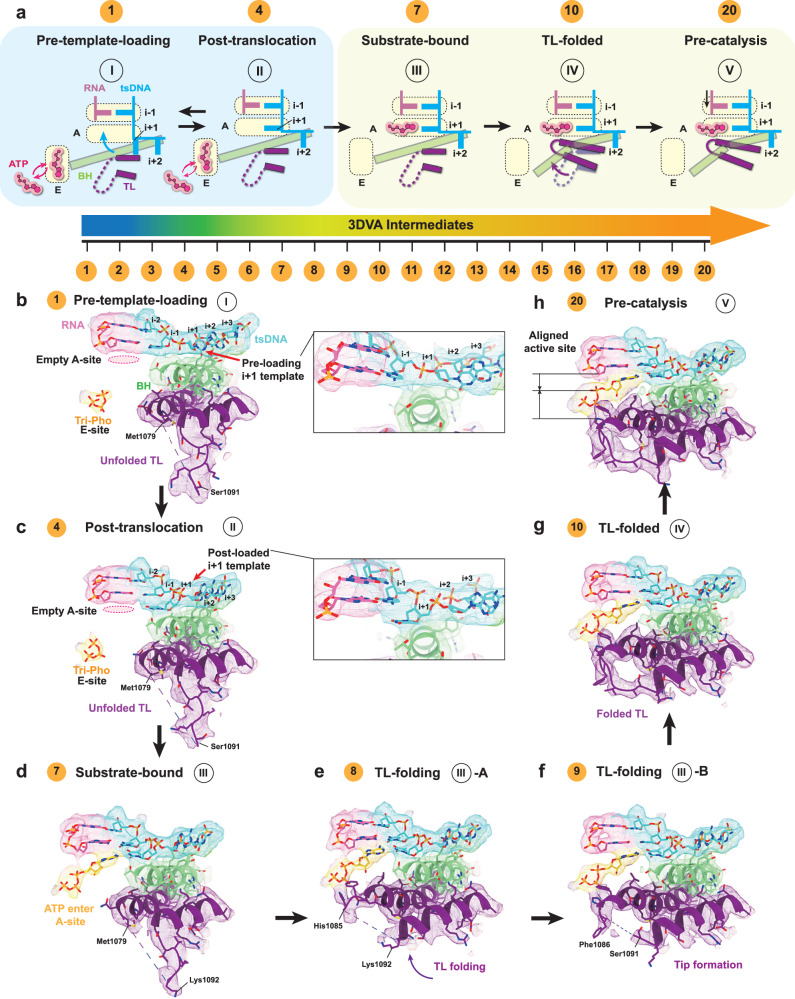
Structural dynamics of the TL folding and substrate loading. **a** Cartoon scheme illustrates the conformational dynamics and state transition in RNAPII’s active site during the substrate loading. A dynamic spectrum containing 20 intermediates (PC0) is shown in the lower panel. Cartoons schemes of five key states of transcription are shown in upper panel, including the pre-template-loading (State I, Frame 1), post-translocation (State II, Frame 4), substrate-bound (State III, Frame 7), TL-folded (State IV, Frame 10), and pre-catalysis (State V, Frame 20) states. RNAPII domains involved conformational dynamics are shown, including the TL (purple), bridge helix (light green), RNA primer & ATP substrate (hot pink), and template DNA (cyan). Frame indexes of key states are indicated and corresponding to the dynamic spectrum. **b–h** Cryo-EM structures of transcriptional states corresponding to the dynamic spectrum in (**a**), including five key states (**b**,**c**,**d**,**g**,**h**) as (**a**) shown and two intermediate states demonstrating the stepwise folding of TL (**e**,**f**). The color codes for RNAPII domains correspond to the cartoon scheme in (**a**). The cryo-EM densities are shown at a uniform contour level of 0.12 (4-σ). Detailed structures of the i + 1 template nucleotide in the pre-template-loading (**b**) and post-translocation (**c**) states, highlighting differences in the nucleotide positioning between these two states. Abbreviations, full names, and color assignments of all RNAPII domains used throughout this figure are provided in Supplementary Table 6.

Intriguingly, the pre-template-loading state was also observed in the RNAPII EC- substrate-binding sample (Sample 2; Fig. 2a, b; Supplementary Movie 2), indicating that the transition from pre-template-loading to post-translocation states can occur with or without a substrate in the entry site (E-site). Electron density corresponding to the ATP triphosphate moiety was detected at the E-site, whereas the nucleobase was unresolved, likely due to conformational flexibility. These observations suggest that ATP can associate with the E-site prior to template base loading.

In the same sample, we also captured the post-translocation state (State II), in which ATP remains bound at the E-site and has not yet transferred into the A-site (Fig. 2c). We further identified a third intermediate (State III), the substrate-bound state (Fig. 2d), where RNAPII adopts a post-translocation conformation, ATP occupies the A-site, and the TL remains open. Together, these structures (States I–III) suggest that the substrate may initially bind non-specifically at the E-site, and that correct base pairing occurs only after template loading, guiding the selective transfer of the nucleotide into the A-site.

### Conformational dynamic intermediates upon substrate binding

**Fig. 3:**
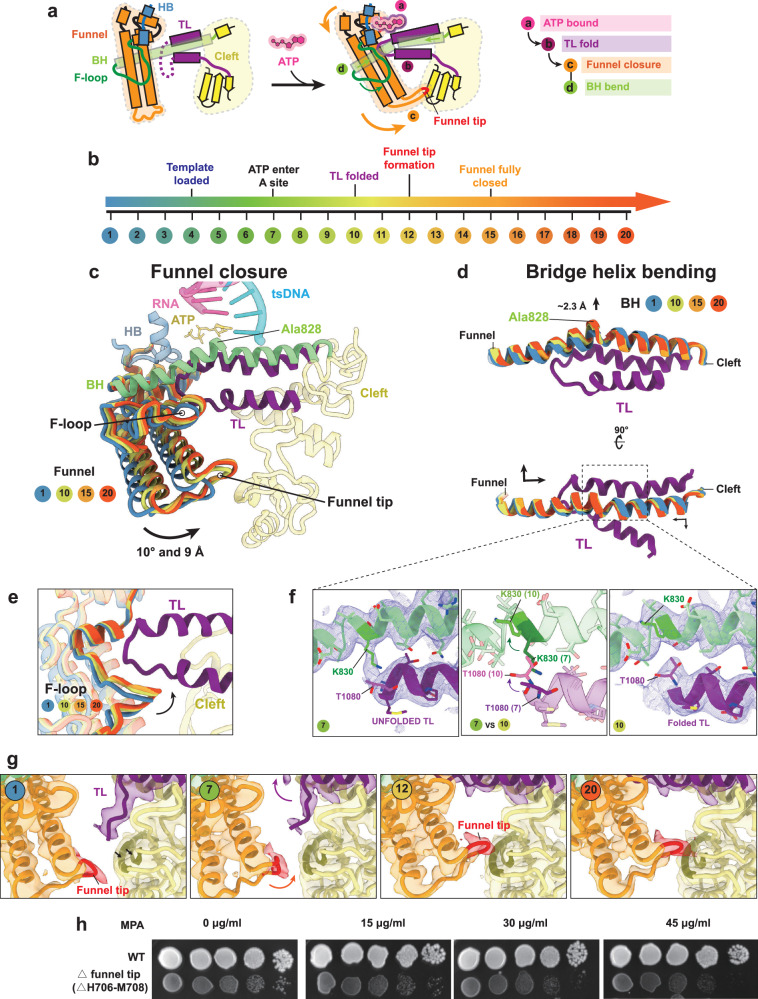
TL folding coordinates with BH bending and funnel closure. **a** Schematic illustration depicting the stepwise conformational changes associated with substate ATP binding, TL folding, BH bending and funnel closure. The tip region of funnel domain is highlighted in red. Abbreviations, full names, and corresponding color codes for all RNAPII domains are provided in Supplementary Table 6. **b** Frame spectrum represents dynamics across 20 cryo-EM intermediates (PC0) in Sample 2 with a gradient color from blue (early) to red (late). Annotated Landmark events indicate key structural rearrangements along the trajectory. **c–e** Structural comparisons of the funnel domain (**c**), BH (**d**), and F-loop (**e**) across selected 3DVA intermediates (frames 1, 10, 15, and 20). Arrows indicate domain movements from frame 1 to frame 20. Colors follow the 3DVA spectrum in (**b**); all other RNAPII elements are shown from frame 20 and colored as in (**a**). **f** Coupled dynamics of TL (purple) folding and BH (light green) bending. Unfolded (frame 7, left) and folded (frame 10, right) TL states correspond to the region highlighted in (**d**). Cryo-EM densities (blue mesh) are contoured at 4σ. The middle panel compares unfolded (transparent) and folded (solid) TL conformations with the BH, highlighting rotational changes. **g** Structural dynamics of funnel tip formation and insertion into the cleft domain. Four 3DVA intermediates (frames 1, 7, 12, and 20) are shown, focusing on the funnel tip region. Cryo-EM densities are contoured at 4σ. Black arrows indicate the funnel tip–binding site on the cleft domain; two associated β-strands (Val1282–Val1291 and Tyr1298–Asp1309) are highlighted in olive. **h** Deletion of the Rpb1 funnel tip (His706–Met708) impairs cell growth and confers sensitivity to MPA. Saturated cultures of wild-type and Rpb1 funnel tip-deleted cells were serially diluted 10-fold and spotted onto YPD plates containing the indicated concentrations of mycophenolic acid (MPA). Plates were incubated at 30 °C for 4 days before imaging.

**Fig. 4:**
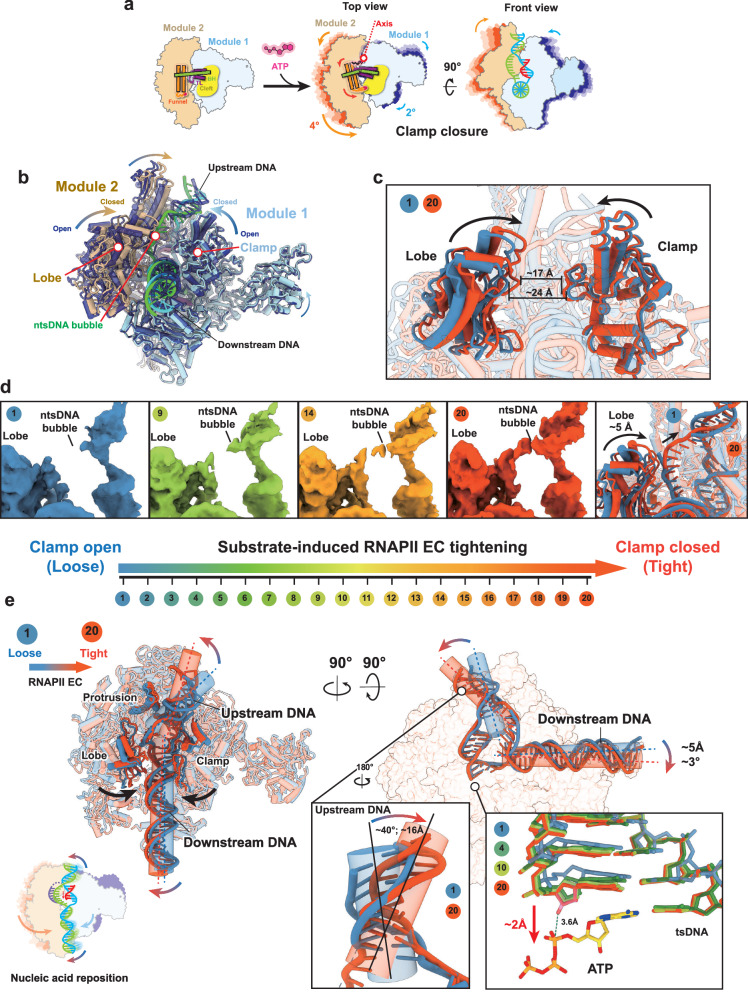
Substrate-induced clamp closure and nucleic acid scaffold repositioning. **a** Schematic of substrate-induced clamp closure. Module 1 (light blue) and module 2 (tan) undergo opposing motions from the pre-template-load state (State I, frame 1) to the pre-catalysis state (State V, frame 20). Arrows indicate the directions of clamp and nucleic acid movements. The rotation axis is shown as a red dashed line. The motion network is centered on the TL (purple), BH (light green), funnel (orange), and cleft (yellow). **b** Structural comparison between States I and V showing global clamp closure. State I is shown in transparent blue and State V in solid colors as in (**a**). Arrows indicate rotations of modules 1 and 2. **c** Clamp closure during RNAPII EC tightening, using the lobe (module 2) and clamp (module 1) domains as reference markers. The shortest distance between the two domains is indicated. **d** Nucleic acid scaffold repositioning during RNAPII EC tightening. Cryo-EM density of the ntsDNA bubble region, contoured at 3σ, shows progressive ordering and stabilization during the transition. **e** Nucleic acid scaffold repositioning and tunnel narrowing during RNAPII EC tightening. The protrusion, lobe, and clamp domains are highlighted. Upstream and downstream DNA trajectories are shown as shaded cylinders, with gradient arrows (blue to red) indicating movement from the loose to tight state. Close-up views show upstream DNA repositioning (left) and alignment of the RNA primer with substrate ATP (right). In the right close-up view, a 3′-OH group was modeled onto the RNA primer, which shows a distance of 3.6 Å to the ATP α-phosphate. A red arrow indicates a ~ 2 Å displacement of the RNA 3′ end during tightening. In the left corner of (**e**), the cartoon schematic summarizes nucleic acid scaffold repositioning. For (**c–e**), intermediates are selected from Sample 2 and indicated in each panel. Colors follow the 3DVA spectrum shown in (**d**). Black arrows indicate module 1 and module 2 motions associated with clamp closure. Abbreviations and full names of RNAPII domains are provided in Supplementary Table 6.

**Fig. 5:**
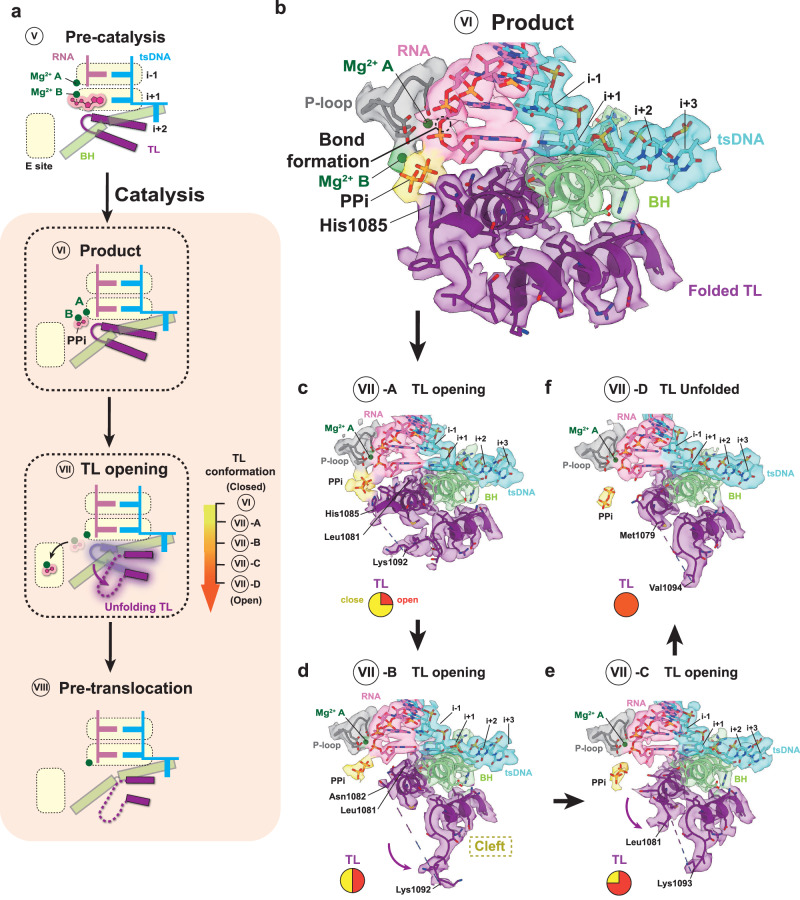
Cryo-EM structures of RNAPII EC at product state and TL-opening state after nucleotide addition. **a** Cartoon schemes illustrate the sequential transition of active center conformational states, including the pre-catalysis state (V), product state (VI), TL-opening state (VII), and pre-translocation state (VIII). A gradient spectrum adjacent to state VII depicts the TL conformational changes across four intermediate substates (VII-A, VII-B, VII-C, and VII-D), ranging from closed (yellow) to fully open (red). Arrows indicate the directions of TL movement and pyrophosphate (PPi) diffusion. **b–f** Cryo-EM structures of RNAPII EC captured at five distinct states: product state (VI, **b**) and TL-opening intermediate states VII-A (**c**), VII-B (**d**), VII-C (**e**), and VII-D (**f**). Cryo-EM density maps are contoured at 5-σ. The coloring and domain annotations correspond to those described in (**a**). Pie charts at the lower-left corners of (**c–f**) indicate the TL conformational distributions, with red representing open and yellow representing closed conformations. Abbreviations, full names, and color assignments of all RNAPII domains used throughout this figure are provided in Supplementary Table 6.

To capture substrate-bound RNAPII intermediates, we utilized an RNA scaffold containing a 3′-dOH modification in Sample 2, which allows nucleotide binding but prevents catalysis. Conformational changes triggered by substrate binding were analyzed using three-dimensional variability analysis (3DVA)^25^, which applies a linear subspace model to cluster and sequentially order particle conformations along principal components (PCs) (Supplementary Table 2), and complementary 3D classification (Supplementary Table 3). The convergence of these independent analyses (Supplementary Fig. 6b–d; Supplementary Tables 2, 3) reinforces the reliability of the captured conformational intermediates. The primary principal component (PC0) defined the dominant trajectory induced by substrate binding, referred to here as the substrate-induced EC tightening (Figs. 2–5). This trajectory reveals a coordinated structural transition, including TL folding (Supplementary Movie 3), funnel closure (Supplementary Movie 4), clamp closure (Supplementary Movie 5), transcription-bubble stabilization (Supplementary Movie 5), and active site alignment (Supplementary Movie 6).

Using particle subsets contributing to each frame, we reconstructed 20 cryo-EM structures representing distinct stages along this conformational trajectory, with global resolutions ranging from 3.3 to 4.3 Å (Fig. 2, Supplementary Fig. 2 and Supplementary Table 2). From these, we identified five primary conformational states: the pre-template-loading state (State I, Frame 1, Fig. 2b), post-translocation state (State II, Frame 4, Fig. 2c), substrate-bound state (State III, Frame 7, Fig. 2d), TL-folded state (State IV, Frame 10, Fig. 2g), and pre-catalysis state (State V, Frame 20, Fig. 2h). The strongest densities for both substrate ATP and the folded TL were observed in State V, indicating a stabilized, catalytically primed conformation. In addition, we resolved two previously uncharacterized TL sub-states (States III-A and III-B, Fig. 2e–f), providing further insight into the dynamic process of TL folding upon substrate engagement. The following sections describe the coordinated local and global conformational dynamics accompanying substrate binding.

### TL folding intermediates upon substrate engagement

A complete spectrum of TL folding intermediates induced by substrate binding was resolved through 3DVA (Supplementary Movie 3). The transition of substrate ATP from the E-site to the A-site is tightly coupled with progressive TL folding. Prior to ATP loading into the A-site (Frames 1 to 6), the TL forms two helical segments (Met1063–Met1079 and Ser1091–Val1107), while the intervening region (Thr1080–Lys1092) remains disordered and lacks detectable density (Fig. 2b, c). Upon ATP engagement at the A-site (Frame 7, State III), the Met1063-Met1079 region remains flexible (Fig. 2d), consistent with the substrate-bound crystal structure (PDB: 2E2I)^12^.

Two intermediate states capture the stepwise process of TL folding (States III-A and III-B; Fig. 2e, f). In State III-A (Frame 8), the Lys1092 to Gly1097 segment detaches from the cleft and adopts a helical structure (Fig. 2e). In State III-B (Frame 9), the Met1079–His1085 region near the active center folds into a helix (Fig. 2f). Ordering of the loop between His1085 and Ala1090 forms a distinct tip, completing the fully folded TL conformation (Frame 10, State IV; Fig. 2g).

Following TL folding, the EC undergoes further rearrangements: The clamp continues to close, accompanied by precise repositioning of the nucleic acid scaffold and ATP. Densities for both ATP and the folded TL become stronger, reflecting a stabilized, catalytically poised conformation (Frame 20, State V, Fig. 2h). This ordered configuration is essential for efficient catalysis and ensures seamless progression through the transcription cycle.

### TL folding couples with BH bending and funnel closure

Substrate-induced TL folding results in a conformational transition that propagates through the RNAPII EC (Fig. 3a, b; Supplementary Movies 4, 5). Upon folding, the TL tip inserts into a pocket formed by the hybrid-binding domain (HB), bridge helix (BH), and funnel domain (Rpb1 residue Ser663–Leu808 aa) (Fig. 3c), triggering a ~ 10° rotation and ~9 Å displacement of the funnel domain toward the cleft domain (Rpb1 residue Pro810–Asp871, His1059–His1140, Gly1275–Thr1394) (Fig. 3a–c). From Frame 1 (steel blue) to Frame 15 (orange), the funnel domain establishes progressive engagement with the folded TL tip, particularly via residues Ser754–Arg806 (Figs. 3c, 3e). This region, analogous to the F-loop in *E. coli* RNAP, is known to rearrange during TL-folding^26^. Beyond Frame 15, funnel closure reaches a largely converged state, with no substantial differences observed up to Frame 20 (Fig. 3c, e).

Before TL folding (Frame 1–7), the unfolded TL (Lys1092–Ser1096) blocks formation of the funnel tip and its interaction with the β-strand region of the cleft domain (Rpb1 residue Val1282–Val1291 and Tyr1298–Asp1309) (Fig. 3g). As folding progresses, the TL disengages, allowing the funnel tip (Rpb1 residue His706–Met708) to insert and form a stabilizing interface after Frame 12 (Fig. 3c, g). This funnel tip appears to act as a conformational lock that reinforces the TL-closed, catalytically competent state. Notably, Gly707 within this tip is highly conserved (Supplementary Fig. 7). Deletion of the funnel tip impairs yeast growth, evidenced by smaller colonies (Fig. 3h), and increases sensitivity to mycophenolic acid (MPA), a nucleotide-depleting agent used to probe transcription elongation defects^27^ (Fig. 3h), underscoring its functional importance.

TL folding is also tightly coupled to BH bending (Fig. 3a, d). From the pre-template-loading (Frame 1, State I) to the pre-catalysis state (Frame 20, State V), the BH bending edge (Ala828) shifts upward by ~2.3 Å (Fig. 3d, top). A top-down view reveals opposite directional movements of BH segments (Thr809–Ala828 and Val829–Ile848), reflecting a pronounced bending motion (Fig. 3d, bottom). Early in the cycle (Frames 1–7), BH residue Lys830 inserts into the unfolded TL (Fig. 3f, left). During TL folding (Frame 8–10), BH rotation repositions Lys830 upward, avoiding steric clashes with TL residue Thr1080 (Fig. 3f, middle and right), further highlighting the coordinated rearrangements essential for catalysis.

### Substrate-induced RNAPII EC tightening

Conformational dynamics are not restricted to the active site but extend across the entire RNAPII EC through an interaction network that couples local rearrangements to global structural transitions (Fig. 4; Supplementary Fig. 8; Supplementary Movie 5). To analyze these large-scale motions, we partitioned the EC into two structural modules: module 1, primarily composed of Rpb1 (Fig. 4a, b, light blue), and module 2, largely corresponding to Rpb2 (Fig. 4a, b, tan).

Module 1 and module 2 undergo opposing rotations around the RNA-DNA hybrid axis, with module 2 rotating counterclockwise ( ~ 4°) and module 1 rotating clockwise ( ~ 2°) from Frame 1 to Frame 20 (Fig. 4a, e). These rotations are coordinated with the conformational changes observed at the active site, including movements of the TL folding, BH bending, and funnel closure (Fig. 4a). RNAPII adopts a pincer-like architecture in which module 1 and module 2 form two opposing arms, while the nucleic acid scaffold is positioned at the base of the cleft and clamped between them (Fig. 4a, b). Upon substrate binding, these two modules move toward each other along the downstream DNA tunnel, resulting in narrowing of the nucleic acid tunnel and formation of a more compact, closed conformation (Fig. 4a–c). We also found that the Rpb4/7 stalk moves in concert with module 1 in a coupled manner (Supplementary Fig. 8e), although in several intermediate states the stalk was too flexible to be modeled.

To quantitatively characterize this motion, we selected two representative domains from each module: the clamp domain from module 1 and the lobe domain from module 2, which are positioned on opposite sides of the downstream DNA tunnel. During clamp closure, the distance between these domains decreases from ~24 to ~17 Å (Fig. 4c), providing direct structural evidence for tunnel narrowing.

To elucidate the pathways underlying these global conformational changes, we mapped an interaction network that connects local rearrangements at the active site to distal regions of RNAPII (Supplementary Fig. 9a, b). Substrate binding induces structural changes within the active site that are propagated through this network, linking active-site elements with surrounding domains and reshaping the overall RNAPII conformation. Individual conformational events were resolved along the 3DVA trajectory and mapped onto the corresponding frame spectrum (Supplementary Fig. 9c), capturing the conformational continuum underlying the transition. Together, these results describe a substrate-induced, coordinated conformational transition of RNAPII that corresponds to progressive EC tightening.

Because Sample 2 used a short RNA scaffold lacking upstream RNA, the observed tightening could potentially reflect the absence of upstream RNA rather than an intrinsic substrate-induced conformational change. To address this possibility, we reconstituted an RNAPII EC using an extended RNA scaffold containing a 19-mer RNA, designed to mimic a more mature elongation complex. The complex was assembled using the same strategy as in Sample 2, with a 3′-dOH RNA primer in the presence of ATP substrate. From this sample, referred to as Sample 4, we resolved two structures corresponding to the TL-closed and TL-open states at resolutions of 2.5 Å and 2.6 Å, respectively (Supplementary Fig. 5; Supplementary Table 5). The TL-closed state closely corresponds to Frame 20, State V, in Sample 2, whereas the TL-open state resembles the substrate-bound state, Frame 7, State III.

Notably, clear density was observed for RNA extending into the exit tunnel, confirming proper assembly of a fully formed, mature elongation complex containing upstream RNA (Supplementary Fig. 10a). Structural comparison between the TL-open and TL-closed states revealed consistent conformational transitions, including TL folding, funnel closure, BH bending, clamp closure, and coordinated repositioning of the nucleic acid scaffold (Supplementary Fig. 10b–e). These observations demonstrate that substrate-induced EC tightening occurs in both short RNA scaffolds and mature elongation complexes containing upstream RNA.

### Nucleic acid scaffold repositioning couples to substrate alignment for catalysis

Accompanying clamp closure and global RNAPII tightening, the nucleic acid tunnel narrows, leading to a coordinated repositioning of the nucleic acid scaffold within the EC (Fig. 4d, e). These changes involve the non-template DNA strand (ntsDNA) of the transcription bubble (Fig. 4d), as well as the downstream and upstream DNA duplexes, and the RNA-DNA hybrid (Fig. 4e).

Within the transcription bubble, the ntsDNA region undergoes pronounced reorganization. As clamp closure proceeds, the lobe domain of module 2 shifts ~5 Å upward and pushes against the ntsDNA, promoting progressive ordering of this region (Fig. 4d; Supplementary Fig. 8c). The downstream DNA duplex exhibits relatively modest movement, coordinated with the lower jaw domain of module 1, undergoing a ~ 3° rotation and a ~ 5 Å displacement (Fig. 4e; Supplementary Fig. 8e). In contrast, the upstream DNA shows pronounced rearrangement. This motion is coordinated with module 2 and involves direct interactions with the protrusion domain, wall domain, and Rpb12 subunit (Fig. 4d; Supplementary Fig. 8e). The upstream DNA undergoes an approximately ~40° rotation and a ~ 16 Å displacement, reflecting large-amplitude repositioning (Fig. 4e).

In contrast to other regions of the nucleic acid scaffold, the RNA-DNA hybrid undergoes relatively minor positional changes. However, these subtle adjustments are functionally important. From State I to State V (Frame 1 to Frame 20), the RNA 3′-terminal nucleotide progressively approaches the ATP substrate by ~2 Å (Fig. 4e, right closed-up view; Supplementary Fig. 8d). In the pre-catalysis state (State V), a 3′-OH group was modeled onto the RNA primer, revealing a closest distance of 3.6 Å to the ATP α-phosphate, consistent with an optimal geometry for nucleophilic attack and coordination with the catalytic metal ions. These observations highlight that even small adjustments of the RNA-DNA hybrid are critical for achieving precise catalytic alignment and promoting efficient nucleotide incorporation.

### Post-catalysis structures and conformational states

Capturing RNAPII EC conformations immediately after phosphodiester bond formation has long been a challenge due to the rapid kinetics of nucleotide incorporation, leaving a critical gap in our understanding of the complete NAC. To overcome this, we developed a substrate-gradient strategy on cryo-EM affinity grids, enabling the capture of transient post-catalysis intermediates (see Methods for details). Using this approach, we established a native RNAPII EC assembled with an unmodified RNA primer and all four NTP substrates (ATP, UTP, CTP, GTP), allowing RNA extension. In this sample (Sample 3), we resolved five previously uncharacterized post-catalysis structures (States VI and VII-A–VII-D), in addition to the states observed in Sample 2 (States II and III) (Fig. 5; Supplementary Figs. 3–4; Supplementary Table 4).

The earliest post-catalysis intermediate, State VI, features a nascent phosphodiester bond with the TL remaining in a closed conformation, representing the product state (Fig. 5a [VI], 5b; Supplementary Movie 7). In this state, pyrophosphate (PPi) remains bound at the A-site, interacting with TL residue His1085 (Fig. 5b; Supplementary Fig. 11). Subsequent intermediates (States VII-A–VII-D) capture a stepwise TL opening and unfolding process (Fig. 5a [VII], 5c–f; Supplementary Movie 8). In State VII-A, the TL tip (Phe1084–Ser1091) becomes disordered (Fig. 5c). In State VII-B, the TL helix (Lys1092–Val1107) continues to unfold, with the loop (Lys1092–Gly1097) re-engaging the cleft domain while the opposing helix (Met1063–His1085) begins to destabilize and His1085 disengages from PPi (Fig. 5d). In State VII-C, the TL segment Met1063–Leu1081 unfolds further, and Leu1081 releases from the RNA 3′-end base (Fig. 5e). State VII-D captures the fully open TL, coinciding with PPi relocation to the E-site, consistent with TL opening creating the clearance required for PPi release (Fig. 5f).

### Post-catalysis RNAPII EC relaxation

**Fig. 6:**
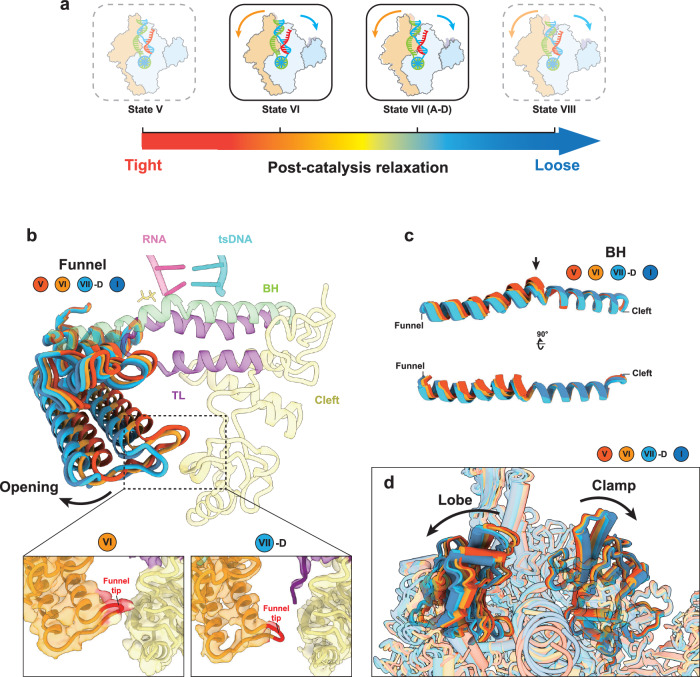
Post-catalysis RNAPII EC relaxation revealed by Sample 3. **a** Schematic representation of the conformational spectrum of RNAPII EC in Sample 3. Four states (states V–VIII) are arranged along a linear spectrum, transitioning from a tight conformation (red, state V) to a loose conformation (blue, state VIII). Cartoon representations of RNAPII EC are shown for each state, with module 1 and module 2 colored in light blue and light orange, respectively. Arrows indicate the relative rotational movement between the two modules. States that were not resolved in Sample 3 are shown as transparent and outlined by dashed lines, whereas states classified from Sample 3 (states VI and VII) are enclosed by solid outlines. **b–d** Structural comparison of local conformational changes across different states. Superposition of selected structures highlights conformational dynamics in the funnel helix (**b**), bridge helix (**c**), and lobe/clamp domain (**d**). Two states derived from the Sample 3 (states VI and VII) are compared together with reference states representing the extremes of the conformational spectrum from Sample 2 (frame 1 at state I, loosest; frame 20 at state V, tightest). Color coding is consistent with conformational spectrum in (**a**). Abbreviations and full names of all RNAPII domains used throughout this figure are provided in Supplementary Table 6.

Coupled with PPi generation and TL opening in the active site, the RNAPII EC undergoes a coordinated, global transition toward a more relaxed state. This process represents the reverse of the tightening transition observed during the pre-catalysis phase (Sample 2 and 4) and is therefore defined as post-catalysis EC relaxation. In Sample 3, such a transition is captured through a series of structural intermediates spanning State VI to State VII (A–D) (Fig. 6a).

Structural comparisons across samples illustrate this relaxation process. Specifically, State VI and State VII-D from Sample 3 were analyzed alongside the reference states State V (tight) and State I (open) from Sample 2 (Fig. 6b–d). State VI represents an immediate post-catalysis intermediate that remains in a tightly engaged conformation, closely resembling the pre-catalysis closed state (State V). In contrast, the State VII ensemble adopts progressively more open conformations, indicating a transition away from the catalytically competent state. This comparison reveals a coordinated global relaxation of the EC, accompanied by local structural rearrangements in multiple regions, including funnel opening, BH unbending, and clamp opening. These regional changes together define the transition of the EC from a compact, catalytically poised configuration toward a more relaxed and translocation-ready state (Fig. 6b–d). Notably, this conformational progression mirrors, in reverse, the tightening transition observed during substrate binding in Sample 2 and 4, supporting a unified framework of catalysis-dependent conformational cycling.

## Discussion

### The complete NAC of RNAPII

**Fig. 7:**
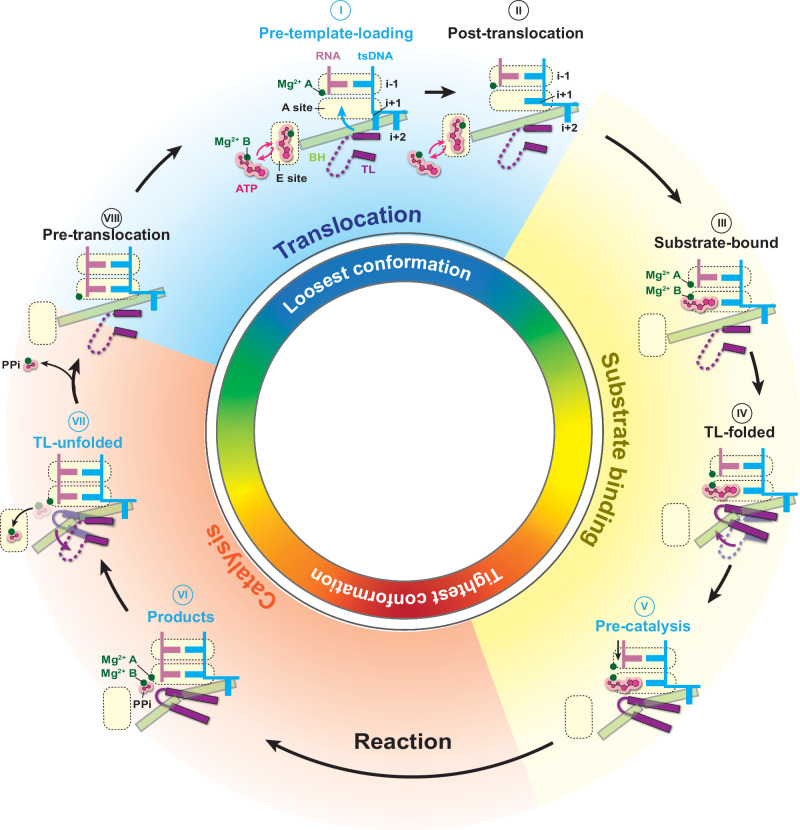
Comprehensive cycle of substrate incorporation by RNAPII EC. Schematic depicting the sequential transition through eight distinct conformational states (states I to VIII) within the active center of RNAPII during the nucleotide addition cycle. States resolved in this study are highlighted with blue annotations. The complete nucleotide incorporation cycle is divided into three main phases, each denoted by a distinct background color: translocation (blue), substrate binding (yellow), and catalysis (orange). The inner cycle displays a color gradient representing clamp conformations ranging from loose (blue) to tight (red). Abbreviations, full names, and color assignments of all RNAPII domains used throughout this figure are provided in Supplementary Table 6.

We propose a complete NAC model for the RNAPII EC, comprising States I–VIII and including four previously uncharacterized intermediates: States I, V, VI, and VII (Fig. 7). These states delineate three major functional stages: translocation (shaded blue), substrate binding (shaded yellow), and catalysis (shaded orange), followed by post-catalysis reset.

The translocation stage (Fig. 7, shaded blue) comprises the pre-translocation state (State VIII), pre-template-loading state (State I), and post-translocation state (State II), which exist in dynamic equilibrium. During this phase, RNAPII adopts a relatively open and flexible conformation with an unfolded TL. The active site remains accessible, allowing incoming NTPs to diffuse nonspecifically into the E site in both States I and II.

The substrate-binding stage (Fig. 7, shaded yellow) begins when a cognate NTP base-pairs with the template at the *i* + 1 position in the A site. In State III, the substrate is bound while the TL remains open. Substrate recognition initiates progressive TL folding (State IV), triggering conformational changes throughout the EC that culminate in the pre-catalysis state (State V). In this state, the TL is fully closed and the RNA 3′-OH is precisely aligned with the NTP α-phosphate, positioning the active site for catalysis.

Following phosphodiester bond formation, the complex enters the post-catalysis stage (Fig. 7, shaded orange). In the immediate product state (State VI), the TL remains closed, stabilizing the EC while metal-coordinated pyrophosphate (PPi) remains bound at the A site. The TL then unfolds (States VIIA–D), clearing space for PPi release. The cycle concludes with the pre-translocation state (State VIII), in which the TL is open, PPi has dissociated, and the active site is reset for the next round of nucleotide addition.

### Dynamic coordination of template loading during translocation

The pre-template-loading state (State I) identified here provides insight into the temporal coordination of template loading and substrate binding within the NAC. This previously uncharacterized intermediate precedes the canonical post-translocation state (State II) and represents the earliest initiation step of the next cycle. Importantly, this state provides direct structural evidence that an NTP can associate with the E-site before the template base is loaded, offering a mechanistic explanation for kinetic observations that nucleotide binding to RNAPII can occur both before and after downstream DNA translocation^15,18^.

This pre-template-loading state differs from the half-translocation intermediate observed in a paused RNAPII EC by Cramer and colleagues^28^. In the paused structure, the RNA has shifted upstream, but the upstream tsDNA remains in a pre-translocation position. In contrast, our structure shows that both the RNA and upstream tsDNA have translocated, whereas the downstream template base has not yet entered the active site. These findings suggest that upstream and downstream translocation could occur in a partially unsynchronized manner, rather than as a concerted movement (at least in the early elongation phase). We note that State I may be transient and difficult to capture, and that our experimental conditions, such as the use of a short RNA scaffold and/or a specific nucleic acid sequence, may have stabilized this state. Further studies will be needed to elucidate the dynamic properties of State I and its potential roles in initiating versus mature elongation complexes.

### A catalysis-associated conformational dynamic linking the active site to global RNAPII architecture

Our study reveals a previously unrecognized catalysis-associated conformational dynamic that extends from the active site to the entire RNAPII EC (Supplementary Movie 1; Supplementary Fig. 9). While earlier studies established that NTP binding promotes TL folding and BH bending^12,29^, the accompanying global architectural changes in RNAPII have remained unclear. Here, we observe large-scale conformational dynamics consistently across multiple samples, including short- and long-RNA scaffolds as well as native NTP-containing conditions, suggesting that this motion represents a general feature of RNAPII.

The global conformational dynamics of RNAPII can be divided into two coordinated phases: a substrate-induced EC tightening phase and a post-catalysis EC relaxation phase. During substrate engagement, conformational changes are initiated within the active site, where TL folding coordinates with funnel rotation and BH bending to form a dynamic core. This core is integrated with the rest of RNAPII through an interaction network, thereby spreading local active-site rearrangements into global structural changes across the complex. At the global level, RNAPII behaves as a pincer-like assembly, with module 1 and module 2 forming two opposing arms that enclose the nucleic acid scaffold at the base of the cleft. Upon substrate loading, these modules undergo coordinated rotations toward the downstream DNA channel, resulting in clamp closure and narrowing of the nucleic acid tunnel. This global tightening is accompanied by repositioning of the nucleic acid scaffold, improving alignment of the RNA primer 3′ terminus with the incoming substrate and establishing a catalytically competent configuration.

This RNAPII tightening is distinct from the swiveling motion described in bacterial RNA polymerase (RNAP) (Supplementary Fig. 12). Bacterial swiveling is primarily associated with transcriptional pausing and translocation-related intermediates^30,31^, where the swivel module rotates approximately parallel to the nucleic acid scaffold (Supplementary Fig. 12a, b). By contrast, the tightening observed here is reproducibly detected upon substrate binding across Samples 2–4, but not in the substrate-free Sample 1, supporting its interpretation as substrate-induced EC tightening. Structurally, this motion involves coordinated movement of RNAPII modules 1 and 2 toward the nucleic acid scaffold, narrowing the cleft in a pincer-like manner (Supplementary Fig. 12c).

Following phosphodiester bond formation, the TL unfolds, accompanied by reopening of the funnel domain, straightening of the BH, and reversal of clamp closure. These changes widen the nucleic acid tunnel and restore RNAPII to a more open, relaxed conformation. We refer to this process as post-catalysis relaxation.

Together, these two phases define a cyclic conformational mechanism in which RNAPII transits between a tightly engaged, reaction-ready state (red/orange) and a relaxed, translocation-competent state (blue/green) (Fig. 7 inner cycle; Supplementary Movie 1). This switching behavior, resembling a molecular spring, links active-site chemistry to global structural transitions and may represent a general mechanism for allosteric regulation in multi-subunit polymerases.

## Methods

### RNAPII purification and elongation complex assembly

*Saccharomyces cerevisiae* 12-subunit RNA polymerase II (RNAPII) was purified and assembled in vitro following procedures described previously^12,32^ with minor modifications. Briefly, a 10-subunit Pol II complex was purified from an in-house-generated *S. cerevisiae* strain carrying a C-terminal TAP tag on the endogenous Rpb3 subunit. Cells were grown in 2×YPD medium at 30 °C for 48 h, harvested by centrifugation, lysed, and clarified by centrifugation. The 10-subunit RNAPII complex was isolated by IgG affinity chromatography followed by heparin and anion-exchange (Q) chromatography. Rpb4 and Rpb7 were co-expressed from an in-house-generated expression plasmid in *Escherichia coli* BL21 (DE3) cells and purified by Ni-NTA affinity chromatography through an N-terminal 6×His tag. Purified Rpb4/7 was mixed with the purified 10-subunit RNAPII complex at a molar ratio of 3:1 and incubated at 4 °C for 1 h. The assembly mixture was further purified by size-exclusion chromatography, and fractions containing fully assembled 12-subunit RNAPII were collected for structural studies.

The 12-subunit RNAPII was further assembled with a biotinylated nucleic acid scaffold to generate the RNAPII elongation complex (EC) as previously reported^16^. All RNA and DNA oligonucleotides used for nucleic acid scaffolds were purchased from Integrated DNA Technologies (IDT). The nucleic acid sequences are as follows: Biotinylated template strand DNA (tsDNA): 5′-/BiotinTEG/ TTT TTT GAT ATT TTT GGA TCC CGC TCT GCT CCT TCT CCC ATC CTC TCG ATG GCT ATG AGA TCA ACT AGG AAT TC-3′; Biotinylated non-template strand DNA (ntsDNA): 5′-/BiotinTEG/ TTT TTA TGT ATT AAT GAA TTC CTA GTT GAT CTC ATA GCC CAT TCC TAC TTG GGA GAA GGA GCA GAG CGG GAT CC-3′; RNA (9-mer): 5′-AUC GAG AGG-3′; RNA (19-mer): 5′-UUU UUU UUU CAU CGA GAG G-3′. Notably, a 3´-deoxy RNA, instead of a regular RNA, was used in the samples of Samples 1, 2, and 4.

### Cryo-EM sample preparation using mspSA affinity grids

We employed mspSA affinity grids for cryo-EM data collection, which effectively overcame the orientation bias and air-water interface issue of RNAPII and improved resolution^16^. The optimized protocol for preparing mspSA affinity grids is outlined as follows: Mix DOPC (18:1 (Δ9-Cis) PC, Avanti) and biotin-labeled lipid (16:0 Biotinyl Cap PE, Avanti) in a 9:1 ratio to reach a final concentration of 1 mg/mL. Prepare a streptavidin solution (streptavidin from Streptomyces avidinii, Sigma) at a concentration of 0.01 mg/mL in a buffer consisting of 20 mM HEPES (pH 7.5) and 150 mM NaCl. Add 30 µL of the streptavidin solution to a Teflon well (on ice), and then carefully deposit 1 µL of the lipid mixture onto the surface of the streptavidin solution. Enclose the Teflon block in a humidity chamber containing a small amount of Milli-Q water at the bottom of the chamber. Seal the lid of the chamber with Vaseline and incubate overnight at room temperature. On the following day, carefully place the carbon side of holy carbon grids (Quantifoil copper 2/1, 300 mesh) onto the surface of the Teflon well (without prior glow discharge) for 2 minutes to allow for lipid monolayer transfer. Wash the grid with three drops (120 µL) of sample buffer to remove unbound streptavidin. Add 4 µL of the RNAPII EC samples to affinity grids and incubate for approximately 5 minutes. Subsequently, plunge-freeze the grids using the Thermo Fisher Vitrobot IV, applying the following parameters: 4 s blotting time, −10 blotting force, at 4 °C and 100% humidity with adding 4 µL of sample buffer. The blotting force should be optimized to achieve the optimal vitrification results.

Four samples with different ECs and substrates were prepared for cryo-EM data collection, including the apo EC containing 9-mer 3´-deoxy RNA (Sample 1), EC containing 3´-deoxy 9-mer RNA with substrate ATP (Sample 2), EC containing native 9-mer RNA with substrate NTP (Sample 3), and EC containing 3´-deoxy 19-mer RNA with substrate ATP (Sample 4) (Supplementary Figs. 1, 2, 3, and 5). For Sample 1, no substrate was added, and RNAPII EC was directly applied to the affinity grids. For Samples 2 and 4, 2 mM substrate ATP and RNAPII EC were pre-mixed for 5 min at room temperature before application onto the affinity grids.

For Sample 3, to capture transient elongation states during active nucleotide addition, we employed a substrate-gradient affinity grid (SGAG) strategy. Specifically, biotin-tagged RNAPII ECs were applied to streptavidin-coated affinity grids and incubated for 5 min to allow stable immobilization. Unbound ECs were subsequently removed by washing twice gently with 50 μL of fresh buffer, and excess buffer was gently absorbed from the edge of the grid using filter paper. The grid was then transferred to a Thermo Fisher Vitrobot IV equilibrated at 4 °C and 100% humidity. Before blotting, 3 μL of fresh buffer was added to the grid surface, followed by application of a small droplet (0.2 μL) of a highly concentrated NTP mixture (10 mM total, containing all four NTPs: ATP, UTP, CTP, and GTP) from the upper edge of the grid. The grid was immediately blotted for 1 s and plunge frozen. During this brief interval, NTPs diffuse and generate a transient concentration gradient across the grid (Supplementary Fig. 3). Sampling across this NTP concentration gradient enables capture of a range of potentially transient intermediate transcriptional states of ECs within the time frame of vitrification (Supplementary Fig. 3).

### Data collection, processing and 3D reconstruction

Initial screening to obtain grids with well-formed monolayers and particles was conducted using a 200 kV Thermo Scientific™ Glacios™ transmission electron microscope. Cryo-EM data were collected using a FEI Titan Krios operating at 300 kV, equipped with a Gatan K3 with GIF Quantum camera (at eBIC, Diamond). Data acquisition was performed automatically using EPU software, with defocus values ranging from −0.5 to −2.5 µm. The imaging pixel size for data collection was 0.829 Å (Bio-Quantum K3) for Sample 1 and Sample 2, 0.825 Å for Sample 3, and 0.932 Å for Sample 4. The total dose applied was 50 electrons per Å² for all samples distributed across 50 frames and Sample 4 was recorded in EER format. A total of 15,165, 12,739, 18,913, and 2000 images were collected for Sample 1–4, respectively.

Data were processed via CryoSPARC (v4.5.3)^33^. Samples 1–4 were analyzed using a similar pipeline, as described below. Raw micrographs were imported, followed by motion correction and calculation of the contrast transfer function (CTF). The resulting micrographs were manually curated to exclude images with poor quality, (such as CTF fit resolution worse than 5 Å; Relative ice thickness: 1<values < 1.1; Total full-frame motion distance (pixels): <60 and astigmatism: <1000). All micrographs were then subjected to automatic particle picking (Blob picking), with particle diameters ranging from 150 to 190 Å. Following several rounds of 2D classification, particles displaying high-quality 2D class averages were selected for ab initio reconstruction, followed by heterogeneous refinement. Particles from well-resolved 3D classes obtained after heterogeneous refinement were used for Topaz training for picking^34,35^. After one or more additional rounds of Topaz training, the resulting high-quality particles were subjected to ab initio reconstruction and further heterogeneous refinement. Particles from well-defined 3D classes, together with high-quality particles obtained through blob picking, were combined, and duplicate entries were removed. The refined particle set was then used for another round of ab initio reconstruction and heterogeneous refinement, followed by homogeneous refinement with defocus refinement and global CTF refinement, enabling the best 3D class obtained from heterogeneous refinement. To separate distinct conformational states across all three samples, 3D classification with a solvent mask was performed without alignment. For Sample 2, 3D Variability Analysis (3DVA)^25^ was conducted in intermediate mode, in which all particles were subdivided into 20 groups and further refined using homogeneous refinement. In all cases, the final resolution was assessed using the gold-standard Fourier shell correlation (FSC) criterion. Local resolution estimation was performed in UCSF Chimera^36^ and ChimeraX^37^ using the output maps generated by CryoSPARC.

In parallel, Sample 2 was subjected to conventional 3D classification employing a solvent mask and omitting alignment procedures. This analysis yielded structural ensembles that delineate a coherent trajectory of temporal conformational transitions—progressing from an open TL to a fully closed state with substrate bound—remarkably concordant with those resolved via 3D Variability Analysis (3DVA) under both simple and intermediate modes (Supplementary Fig. 6). The convergence of these independent analytical modalities not only reinforces the robustness of our findings but also affirms the reproducibility and fidelity of the captured intermediate conformational states.

### Atomic model building, refinement, and validation

We generated a total of 31 atomic models of RNAPII EC from four cryo-EM samples. Specifically, Sample 1 yielded two distinct structures representing the pre-template-loading state (State I) and the post-translocation state (State II). From Sample 2, we resolved 20 structural intermediates of RNAPII EC, covering dynamic transitions corresponding to State I through V, aligned with the principal component analysis (PCA) results of 3DVA. From Sample 3, seven structures were determined, comprising one product state (State VI), four sequential TL opening intermediates (States VII-A to VII-D), one post-translocation state (State II), and one substrate-bound state (State III). For Sample 4, two structures were determined at State III (TL-open) and V (TL-closed).

For all these models, we used the same pipeline for model building, refinement, and validation. The previously published RNAPII EC structure (PDB: 2E2H^12^ and 8U9R^13^) was used as the initial model. The resulting models were rigid-body fitted into the cryo-EM density maps using ChimeraX UCSF (v1.8)^36^. Model refinement was performed in PHENIX (v1.21)^38^ using the phenix.real_space_refine program, followed by manual adjustments in COOT (v0.9.8)^39^. Model validation was conducted with MolProbity^40^. All structural figures were rendered using ChimeraX UCSF (v1.8)^36^.

### MPA sensitivity assay

The funnel tip deletion (Rpb1 H706–M708 deletion) strain was generated in the BJ5465 background (*MAT*a *ura3-52 trp-1 leu2*_*1 his3*_*200 pep4*::*HIS3 prb1*_*1.6 R can1*)^41^ using CRISPR/Cas9. The CRISPR/Cas9 plasmid targeted a 20-nucleotide protospacer sequence (5′-AACTTATTGACTGCTAAAGC-3′) within the yeast RPB1 gene. The donor DNA template for the deletion was generated by annealing the following complementary oligonucleotides:

Forward: 5′-ATGTTACGAAAGAAGCCCAGGCAAACTTATTGACTGCTAAAACTCTCCGTGAGTCTTTTGAGGATAACGTTGTTCGGTTCC-3′

Reverse: 5′-GGAACCGAACAAGCGTTATCCTCAAAAACTCACGGAGAGTTTTAGCAGTCAATAAGTTTGCCTGGGCTTCTTTCGTAACAT-3′.

The successful deletion was verified by Sanger sequencing.

For the MPA sensitivity assay, wild-type and Rpb1 funnel tip-deleted strains were grown in yeast extract peptone dextrose (YPD) medium at 30 °C to stationary phase. Cultures were then serially diluted 10-fold, and 5 µl of each dilution was spotted onto YPD plates supplemented with the indicated concentrations of MPA. Plates were incubated at 30 °C for 4 days before imaging.

### In vitro transcription assay

RNAPII EC samples were assembled using the nucleic acid scaffold (containing regular 9-mer RNA) identical to the one used for cryo-EM sample preparation, with slight modifications in the complex assembly process. Initially, the radioactively labeled RNA primer (regular 9-mer) was annealed with the template DNA (tsDNA). Subsequently, the 12-subunit RNAPII was incubated with the RNA-tsDNA hybrid, followed by the addition of non-template strand DNA (ntsDNA) to complete the assembly of the RNAPII EC. The final ratio of components in the RNAPII EC was as follows: RNAPII: RNA: tsDNA: ntsDNA = 6:1:2:3. RNAPII EC samples were prepared at a final concentration of 40 nM in elongation complex (EC) buffer containing 20 mM Tris-HCl (pH 7.5), 5 mM MgCl_2_, 40 mM KCl, and 5 mM DTT. In vitro transcription was initiated by the addition of an NTP mixture. Three substrate NTP concentrations (5 mM, 1 mM, and 200 μM) were tested here. For each concentration, transcription reactions were terminated at multiple time points: 5 s, 10 s, 30 s, and 10 min. Reactions were quenched by the addition of an equal volume of quench-loading buffer (90% formamide, 50 mM EDTA, 0.05% xylene cyanol, and 0.05% bromophenol blue), followed by heating at 95 °C for 10 min. Reaction products were analyzed by denaturing polyacrylamide gel electrophoresis (PAGE) containing 6 M urea. The gel was visualized by phosphorimaging, and bands were quantified using Image Laboratory software (Bio-Rad).

### Reporting summary

Further information on research design is available in the Nature Portfolio Reporting Summary linked to this article.

## Supplementary information

Supplementary Information

Description of Additional Supplementary Files

Supplementary Movie 1

Supplementary Movie 2

Supplementary Movie 3

Supplementary Movie 4

Supplementary Movie 5

Supplementary Movie 6

Supplementary Movie 7

Supplementary Movie 8

Reporting Summary

Transparent Peer Review file

## Source data

Source Data

## Data Availability

All data needed to evaluate the conclusions in the paper are present in the paper and/or the supplementary information. Source Data are provided with this paper. Cryo-EM density maps and corresponding atomic coordinates have been deposited in the Electron Microscopy Data Bank (EMDB) and Protein Data Bank (PDB), respectively. Accession codes are as follows: Maps and/or atomic models of RNAPII EC-Apo (Sample 1) have been deposited under accession code for State-I (EMD-54704 /PDB 9SAY), State - I-A (EMD-54705), State-I-B (EMD-54706) and State-II (EMD-54707 /PDB 9SAZ). Accession codes of maps and atomic models of RNAPII EC in complex with ATP (Sample 2) generated from 3D variability and 3D display (Intermediate model, Window=0) are EMD-54730/ PDB 9SBL, EMD-54731/ PDB 9SBM, EMD-54732/ PDB 9SBN, EMD-54733/ PDB 9SBO, EMD-54734/ PDB 9SBP, EMD-54735/ PDB 9SBQ, EMD-54736/ PDB 9SBR, EMD-54737/ PDB 9SBS, EMD-54738/ PDB 9SBT, EMD-54739/ PDB 9SBU, EMD-54740/ PDB 9SBV, EMD-54741/ PDB 9SBW, EMD-54742/ PDB 9SBX, EMD-54743/ PDB 9SBY, EMD-54744 / PDB 9SBZ, EMD-54745 / PDB 9SC0, EMD-54746 / PDB 9SC1, EMD-54747 / PDB 9SC2, EMD-54748 / PDB 9SC3 and EMD-54749 / PDB 9SC4 (ordered from Frame1 to Frame 20). Additional maps obtained from 3D classification (with solvent mask) have been deposited separately: 3D Class A (Frame 1) (EMD-54677), 3D Class B (Frame 4) (EMD-54682), 3D Class C (Frame 7) (EMD-54681), 3D Class D (Frame 8) (EMD-54680), 3D Class E (Frame 9) (EMD-54679), 3D Class F (Frame 10) (EMD-54683), 3D Class G (Frame 13) (EMD-54684), 3D Class H (Frame 15) (EMD-54685), 3D Class I (Frame 17) (EMD-54686), and 3D Class J (Frame 20) (EMD-54687). Maps and atomic models of RNAPII EC in complex with NTP (Sample 3) have been deposited under accession code for State-II (EMD-54713 /9SB5), State-III (EMD-57322/29RF), State-VI (EMD-54708 / PDB 9SB0), State-VII-A (EMD-54709 / PDB 9SB1), State-VII-B (EMD-54710 / PDB 9SB2), State-VII-C (EMD-54711 / PDB 9SB3), and State-VII-D (EMD-54712 / PDB 9SB4). Accession codes of maps and atomic models of long-RNA EC in complex with ATP (Sample 4) are State III (TL-open) (EMD-57673 /PDB 30EP) and State-V (TL-closed) (EMD-57676/PDB 30ES). See Supplementary Table 7 for detailed descriptions of each deposited dataset, along with the corresponding EMDB entry URLs and PDB entry DOIs. Source data are provided with this paper.
