# Supplementary material for: Structural dynamics of RNA polymerase II throughout the nucleotide addition cycle
Source: Nat Commun. 2026 Jul 8;17:8440. doi: 10.1038/s41467-026-75257-5 (PMC13478619; doi:10.1038/s41467-026-75257-5)
Supplement: Supplementary file 1 [file 41467_2026_75257_MOESM11_ESM.pdf]

nature portfolio

Corresponding author(s): Peijun Zhang, Dong Wang

Last updated by author(s): Jun 16, 2026

Reporting Summary

Nature Portfolio wishes to improve the reproducibility of the work that we publish. This form provides structure for consistency and transparency in reporting. For further information on Nature Portfolio policies, see our Editorial Policies and the Editorial Policy Checklist.

Statistics

For all statistical analyses, confirm that the following items are present in the figure legend, table legend, main text, or Methods section.

n/a	Confirmed	
☐	☒	The exact sample size (*n*) for each experimental group/condition, given as a discrete number and unit of measurement
☐	☒	A statement on whether measurements were taken from distinct samples or whether the same sample was measured repeatedly
☒	☐	The statistical test(s) used AND whether they are one- or two-sided<br>*Only common tests should be described solely by name; describe more complex techniques in the Methods section.*
☒	☐	A description of all covariates tested
☒	☐	A description of any assumptions or corrections, such as tests of normality and adjustment for multiple comparisons
☒	☐	A full description of the statistical parameters including central tendency (e.g. means) or other basic estimates (e.g. regression coefficient) AND variation (e.g. standard deviation) or associated estimates of uncertainty (e.g. confidence intervals)
☒	☐	For null hypothesis testing, the test statistic (e.g. *F*, *t*, *r*) with confidence intervals, effect sizes, degrees of freedom and *P* value noted<br>*Give P values as exact values whenever suitable.*
☒	☐	For Bayesian analysis, information on the choice of priors and Markov chain Monte Carlo settings
☒	☐	For hierarchical and complex designs, identification of the appropriate level for tests and full reporting of outcomes
☒	☐	Estimates of effect sizes (e.g. Cohen's *d*, Pearson's *r*), indicating how they were calculated

Our web collection on statistics for biologists contains articles on many of the points above.

Software and code

Policy information about availability of computer code

Data collection	EPU software (Thermo Fisher Scientific, version 2.7)
Data analysis	cryoSPARC (v4.5.3), phenix (v1.21), ChimeraX UCSF (v1.8) , COOT (v0.9.8), Python(v3.9.0)

For manuscripts utilizing custom algorithms or software that are central to the research but not yet described in published literature, software must be made available to editors and reviewers. We strongly encourage code deposition in a community repository (e.g. GitHub). See the Nature Portfolio guidelines for submitting code & software for further information.

Data

Policy information about availability of data

All manuscripts must include a data availability statement. This statement should provide the following information, where applicable:

- Accession codes, unique identifiers, or web links for publicly available datasets
- A description of any restrictions on data availability
- For clinical datasets or third party data, please ensure that the statement adheres to our policy

All data needed to evaluate the conclusions in the paper are present in the paper and/or the supplementary information. Source Data are provided with this paper. Cryo-EM density maps and corresponding atomic coordinates have been deposited in the Electron Microscopy Data Bank (EMDB) and Protein Data Bank (PDB), respectively. Accession codes are as follows: Maps and/or atomic models of RNAPII EC-Apo (Dataset 1) have been deposited under accession code for State-I (EMD-54704/PDB 9SAY), State-I-A (EMD-54705), State-I-B (EMD-54706) and State-II (EMD-54707/PDB 9SAZ). Accession codes of maps and atomic models of RNAPII EC in complex with ATP (Dataset 2) generated from 3D variability and 3D display (Intermediate model, Window=0) are EMD-54730/ PDB 9SBL, EMD-54731/ PDB

9SBM, EMD-54732/ PDB 9SBN, EMD-54733/ PDB 9SBO, EMD-54734/ PDB 9SBP, EMD-54735/ PDB 9SBQ, EMD-54736/ PDB 9SBR, EMD-54737/ PDB 9SBS, EMD-54738/ PDB 9SBT, EMD-54739/ PDB 9SBU, EMD-54740/ PDB 9SBV, EMD-54741/ PDB 9SBW, EMD-54742/ PDB 9SBX, EMD-54743/ PDB 9SBY, EMD-54744/ PDB 9SBZ, EMD-54745/ PDB 9SC0, EMD-54746/ PDB 9SC1, EMD-54747/ PDB 9SC2, EMD-54748/ PDB 9SC3 and EMD-54749/ PDB 9SC4 (ordered from Frame1 to Frame 20). Additional maps obtained from 3D classification (with solvent mask) have been deposited separately: 3D Class A (Frame 1) (EMD-54677), 3D Class B (Frame 4) (EMD-54682), 3D Class C (Frame 7) (EMD-54681), 3D Class D (Frame 8) (EMD-54680), 3D Class E (Frame 9) (EMD-54679), 3D Class F (Frame 10) (EMD-54683), 3D Class G (Frame 13) (EMD-54684), 3D Class H (Frame 15) (EMD-54685), 3D Class I (Frame 17) (EMD-54686), and 3D Class J (Frame 20) (EMD-54687). Maps and atomic models of RNAPII EC in complex with NTP (Dataset 3) have been deposited under accession code for State-II (EMD-54713/9SB5), State-III (EMD-57322/29RF), State-VI (EMD-54708/ PDB 9SB0), State-VII-A (EMD-54709/ PDB 9SB1), State-VII-B (EMD-54710/ PDB 9SB2), State-VII-C (EMD-54711/ PDB 9SB3), and State-VII-D (EMD-54712/ PDB 9SB4). Accession codes of maps and atomic models of long-RNA EC in complex with ATP (Dataset 4) are State III (TL-open) (EMD-57673/PDB 30EP) and State-V (TL-closed) (EMD-57676/PDB 30ES).

Research involving human participants, their data, or biological material

Policy information about studies with human participants or human data. See also policy information about sex, gender (identity/presentation), and sexual orientation and race, ethnicity and racism.

Reporting on sex and gender	N/A
Reporting on race, ethnicity, or other socially relevant groupings	N/A
Population characteristics	N/A
Recruitment	N/A
Ethics oversight	N/A

Note that full information on the approval of the study protocol must also be provided in the manuscript.

Field-specific reporting

Please select the one below that is the best fit for your research. If you are not sure, read the appropriate sections before making your selection.

[x] Life sciences [ ] Behavioural & social sciences [ ] Ecological, evolutionary & environmental sciences

For a reference copy of the document with all sections, see nature.com/documents/nr-reporting-summary-flat.pdf

Life sciences study design

All studies must disclose on these points even when the disclosure is negative.

Sample size	For example, in the context of EM data collection, we acquired over 10,000 images across sample-1/2/3 and an additional 2,000 images for sample4 for data processing. We consider this number sufficient for our analysis.
Data exclusions	In the cryo-EM process, particles were excluded during 2D classification and 3D reconstruction following standard protocols in the field.
Replication	To ensure reproducibility, similar raw cryo-EM micrographs were captured over three separate occasions, and the RNAPII elongation assay yielded consistent results on three independent trials. Final cryo-EM data collection, however, was conducted using a single representative sample for each condition.
Randomization	For the dataset of Cryo-EM SPA study, the dataset was randomly divided into several sets for different particle picking strategies, as a standard approach implemented in CryoSPARC.
Blinding	Blinding was not relevant for this study, structure of this RNA Polymerase II was known to all researcher involved in solving this structure.

Reporting for specific materials, systems and methods

We require information from authors about some types of materials, experimental systems and methods used in many studies. Here, indicate whether each material, system or method listed is relevant to your study. If you are not sure if a list item applies to your research, read the appropriate section before selecting a response.

Materials & experimental systems

n/a	Involved in the study
☒	☐ Antibodies
☒	☐ Eukaryotic cell lines
☒	☐ Palaeontology and archaeology
☒	☐ Animals and other organisms
☒	☐ Clinical data
☒	☐ Dual use research of concern
☒	☐ Plants

Methods

n/a	Involved in the study
☒	☐ ChIP-seq
☒	☐ Flow cytometry
☒	☐ MRI-based neuroimaging

Plants

Seed stocks	N/A
Novel plant genotypes	N/A
Authentication	N/A
