# Supplementary material for: Structural dynamics of RNA polymerase II throughout the nucleotide addition cycle
Source: Nat Commun. 2026 Jul 8;17:8440. doi: 10.1038/s41467-026-75257-5 (PMC13478619; doi:10.1038/s41467-026-75257-5)
Supplement: Supplementary file 2 [file 41467_2026_75257_MOESM12_ESM.pdf]

nature portfolio

Peer Review File

Structural Dynamics of RNA Polymerase II Throughout the Nucleotide Addition Cycle

Corresponding Author: Professor Peijun Zhang

This file contains all reviewer reports in order by version, followed by all author rebuttals in order by version.

Version 0:

Reviewer comments:

Reviewer #1

(Remarks to the Author)
Yi et al. report an analysis of the conformational changes that occur during the nucleotide addition cycle (NAC) of yeast RNA polymerase II (RNAPII). Their findings are made possible by recent advances in computational methods that sort cryo-EM particles into different conformational states and a clever approach to tether RNAPII transcription elongation complexes (ECs) to a cryo-EM grid in a way that enables diverse particle orientations. The authors use three different EC configurations to obtain ECs in different stages of the NAC: (1) a substrate free EC with an 8mer 3'deoxy RNA (5'–AUCGAGAG); (2) the same 3'deoxy EC incubated with ATP; and (3) the same EC containing 3'OH 8mer RNA allowed to react with NTPs. The authors assemble states observed in these three different populations into eight conformations that they connect as intermediates in the NAC, as well as sub-conformations like different stages of trigger loop folding. These eight states include some that have not been clearly observed previously, including the post-catalytic complex with bound pyrophosphate and an NTP-bound state that precedes NTP positioning in the catalytic pose. In addition they define movements of two previously undefined structural modules in RNAPII in concert with steps in the NAC. Overall the results will be of broad interest to researchers interested in transcription. The cryo-EM work appears to be expertly conducted and the method of tethering complexes to grids is an important advance. The work merits publication. However, there are a couple major limitations to the conclusions that currently are not acknowledged in the manuscript as well as points that require additional information or that require additional analysis. These concerns will need to be addressed in a revised manuscript before the manuscript can be judged to be appropriate for publication.

1. A major limitation of the results is that they are obtained with ECs lacking upstream RNA. A true EC contains a 10–11 bp RNA–DNA hybrid and single-stranded RNA in its RNA exit channel. The 8mer RNA used here will generate complexes that are more analogous to initially transcribing complexes (ITCs) that form when RNAPII begins synthesis from a promoter but has not yet transitioned to a fully formed EC. However, they are not true ITC mimics because they lack a 5'-triphosphate (as well as initiation factors like TFIIB). The lack of initiation factors is not a concern, but the use of an incomplete EC is a concern. First, it is unclear if the all of the motions described by the authors would occur in a mature EC. Second, the use of an RNA that does not fill the RNAPII cleft means that the authors cannot visualize the configuration of the upstream fork junction where RNA separates from DNA. The upstream fork junction functions in concert with the downstream fork junction where DNA is opened to insert the template DNA into the active site.
Thus, the scaffold chosen for the authors study introduces major limitations to their conclusions. It is possible that the clamp (module 1) and module 2 motions observed are specific to the pseudo-ITC used and would not occur in an EC that makes all upstream RNA contacts. Similarly, the upstream shift of the RNA–DNA hybrid observed in 'state I' may occur when the hybrid-binding cleft is not completely filled but may not be integral to the NAC in a mature EC. Finally, how do we know that the conformations reported for datasets 1 and 2 might be seen only when an artifactual 3'-deoxyRNA is present in the EC? These limitation must be clearly explained and addressed in the manuscript.

2. It's possible that the authors observe some of the movements and states described for the static complexes in their dataset of EC+NTPs (dataset 3), but this question does not appear to be addressed specifically in the manuscript. Further, what is the state of these ECs under the conditions the data are collected? How many rounds of nucleotide addition have occurred? What are the positions of modules 1 and 2 in complexes VI–VIII and are motions of modules 1 and 2 seen in this dataset? Were complexes I–V seen in this dataset? If multiple rounds of nucleotide addition have occurred in dataset 3, what configurations of the upstream fork junction are seen? All of these questions need to be considered and addressed in a revised manuscript.

3. The authors describe the conformational changes they detect using a causal framework. For example, they suggest funnel closure drives the rotation of module 2 and that bridge helix bending drive movement of module 1 (lines 271–272). These statements imply that the funnel or bridge helix movements occur first and then cause the movements of modules 1 and 2. It's unclear that causal relationships can be discerned from the authors datasets. In general, thermal fluctuations cause RNAPII to assume different conformational states but these effects of thermal energy don't necessarily operate specifically on one component of RNAPII that transmits an effect to another component. Rather, thermal energy can cause conformational changes via impacts on fluctuations of any component, which are linked to movements of other components through a complex network of interactions. Unless the authors can describe a rationale for the causal relationships they suggest, they should reframe these descriptions of movement as being linked rather than being driven in one direction.

4. What is the relationship between the module 1 – module 2 rotations observed in RNAPII and the swiveling motions observed in bacterial RNAP (e.g., Zhu et al. 2022 Nat Commun 13:1546 doi:10.1038/s41467-022-29148-0). I'm pretty sure they are not similar, although rotations of the clamp domain appear to be involved in both. The point of comparing the motions in RNAPII and bacterial RNAP is not to suggest that they are the same, although similarities should be noted. The point is to put the results in a broader evolutionary context. How generalizable are the module 1 – module 2 motions likely to be? Are they also likely to occur in archaeal RNAP, RNAPI, or RNAPIII?

Minor points

1. The authors use Pol II to refer to RNAPII throughout the manuscript. Pol II is ambiguous because it does not distinguish DNA and RNA polymerases. RNAPII is a preferable abbreviation.

2. The scaffold sequence(s) need to be shown in the figures (either in Figure 1 or in Extended Data Figures S1–S3) drawn in a way to show the upstream and downstream DNA duplexes, the transcription bubble, and the RNA–DNA hybrid.

3. The manuscript must identify S. cerevisiae as the source of RNAPII either in the abstract or the first para of the introduction. As currently presented, the reader is left in the dark about whether the paper addresses mammalian or yeast RNAPII, even though specific Rpb1 residues are mentioned without context in the first paragraph of the introduction.

4. line 130. The authors state that spontaneous oscillation of RNAPII is known, but the citation given is to bacterial RNAP.

5. line 391 This conclusion that upstream and downstream translocation are not coupled is an example of a conclusion that may incorrect if it depends on 3'deoxyECs with less than complete RNA–DNA hybrids. Unless these states have been observed in dataset 3 with complete hybrids, this caveat should be stated explicitly in the revised manuscript.

6. What are the conditions used to generate complex 3 (active NTP addition)? In the results, the authors mention a 'substrate-gradient strategy' but don't explain what the strategy consists of (line 314). It's unclear which NTPs are present (all 4?), for how long, and at what temperature for the formation of the complex. The exact conditions used to generate dataset 3 must be given in the methods. The 'substrate-gradient strategy' must be explained in the results and the state of the ECs in control reactions must be documented in a figure. What is the distribution of RNA lengths in reactions that precisely mimic the cryo-EM data collection?

7. If I am interpreting the figures correctly, the +1 DNA nucleotide is colored inconsistently in Figure 1 panels C and D. Why not color it yellow in all the panels to avoid confusion?

8. Are the template nucleotides in Figure 1 panel E misnumbered? At a minimum the use of colors and numbering is confusing. The yellow highlighted nucleotide in panel C is labeled +1 whereas the yellow highlighted nucleotide in panel E is labeled –1. The authors should check the numbering carefully and also think about ways to make the figures easier to follow.

Reviewer #2

(Remarks to the Author)
Yi, Li et al. report new cryo-EM structures that elucidate additional steps of the RNA polymerase II nucleotide addition. Overall, this is an important and exciting contribution to the field and facilitates our understanding of one of the most basic processes in molecular biology. The manuscript is well-written and the figures are especially commendable. These structures will significantly improve our understanding of the nucleotide addition cycle. This reviewer only has a few points that should be addressed by the authors.

Major points
1) The title "A complete structural pathway…" is misleading. The authors simply cannot know if the observed states COMPLETELY capture the structural pathway for nucleotide addition. Perhaps "An expanded structural pathway…" is a better choice for the title.
2) The authors use terms like "near-atomic" or "high" resolution throughout the manuscript. This terminology is highly misleading as the provided structures are only in the realm of 3-4 Å. These resolutions are not even close to being "near atomic"/"high" in nature. Therefore, the authors must refrain from using these terms as they suggest a higher map quality than

is available from the here provided data.
3) With their limited resolution, how are the authors certain about metal assignments?
4) FSC curves and angular distribution plots are lacking.

Minor points
1) Resolution is incorrectly reported to two significant digits beyond the decimal point. I doubt that resolution can be reported this accurately. I suggest the authors only report to a single digit, e.g. 3.0 Å, instead of 2.98 Å.

Reviewer #3

(Remarks to the Author)
Yi, Li et al., present cryo-EM structures of RNA polymerase II over the course of its nucleotide addition cycle. Modern cryo-EM classification approaches as well as clever biochemistry allow for a complete trajectory of the NAC that was previously not possible with X-ray crystallography. This heroic effort captures multiple states that have not been perviously observed, and shows how active site conformations influence the overall conformation of the enzyme. This paper will serve as a critical reference to anyone studying RNA polymerase dynamics and elongation, and will be a model for how to follow a complete enzymatic process using cryo-EM. I have a few comments that the authors should address prior to publication.

-Figure 4 shows many of the changes in RNA polymerase II over the course of the nucleotide addition cycle. The upstream DNA is typically quite flexible, and it would be helpful if the upstream DNA would be discussed/shown in this figure or in the extended data. The Rpb4/7 stalk is also typically quite flexible (and this appears to be the case here too, given the provided 2D classes). It would be helpful to more explicitly describe any potential Rpb4/7 fixed state associated with a particular stage of the nucleotide addition cycle.

-Increase font size of the scale bars in Extended Data Figures 1-3 for the 2D classes.

-2D classes do not show lots of high resolution features. Is this a limitation of the datasets or how it rendered in the figure? There also appears to be contamination in some of the images (eg Extended Figure 1c top left class has a very bright spot and the particles do not appear to be well-aligned). Given the limited number of 2D class images provided, I would recommend that the authors select ones that show high resolution features and were presumably used for downstream map generation.

-It would help if the authors provided a graphical representation of the nucleic acid scaffold used for structure determination in an Extended Data figure.

-For all deposited cryo-EM maps, FSC plots (ideally 3D FSC) and 3D angular distribution plots need to be provided. I do not see these in the extended data.

Version 1:

Reviewer comments:

Reviewer #1

(Remarks to the Author)
The revised manuscript from Yi et al. addresses all the key concerns noted in the initial reviews. The authors have added a new set of cryo-EM structures using a fully mature elongation complex scaffold with a 19-nt RNA to ensure their findings are robust. They also have revised interpretations to be consistent with the stochastic fluctuations of conformations expected to operate at molecular scale. The authors should be commended for such a positive response to peer review, which has substantially improved their manuscript. The manuscript is appropriate for publication with only minor revisions that can easily be accomplished in preparation of a final manuscript. I have only two scientific points to suggest for minor rewording to more accurately state the interpretation of the results and a few minor grammatical or presentation issues that could be improved.

Scientific points

1. The suggestion that NTP associates with the E-site prior to transition to the A-site (lines 160-161) is appropriate but the results don't distinguish between an ordered binding of NTP to the E-site then to the A-site versus random order initial binding to the A- or E-sites with NTPs shuttling between them prior to translocation and complementary NTP selection. I suggest revising this sentence to read "Together, these structures (States I–III) suggest that the substrate may initially bind non-specifically at the E-site." The authors may wish to consider whether to suggest to random-order binding model as an alternative interpretation.

2. In the discussion of whether state I occurs only when the RNA–DNA hybrid is not yet fully established or also occurs in mature elongation complexes (lines 438-439), I think readers would benefit from a more explicit statement of the uncertainty. I suggest just modifying the last sentence of the paragraph to read "Further studies will be needed to elucidate the dynamic properties of State I and its potential roles in initiating versus mature elongation complexes."

Minor points. The manuscript is easy to follow as written but a few grammatical glitches in the intro could be fixed easily. Two issues in Fig 1 still need to be addressed.

1. Line 60 Should be “The NAC….”

2. Line 80, It would suffice to write instead “This product state is short-lived and challenging to capture….”

3. Line 86 would read better as “How does *the* TL fold to a closed conformation upon substrate binding and then unfold after catalysis?”

4. Line 88 would read better as “Does substrate binding lead to allosteric conformational changes in structural motifs beyond the TL, and how are these motions communicated?”

5. Line 96 would read better as “Substrate binding initiates long-range allosteric conformational changes….”

6. In fig. 1 panel C, color i+1 tDNA base yellow as in panels D and E

7. In fig. 1 panel E, move the label i+2 so that it is next to the i_2 base instead of next to the i+3 base where it is in the submitted figure.

Reviewer #2

(Remarks to the Author)
The authors have addressed all my concerns sufficiently.

Reviewer #3

(Remarks to the Author)
The authors have addressed all of my concerns.

Open Access This Peer Review File is licensed under a Creative Commons Attribution 4.0 International License, which permits use, sharing, adaptation, distribution and reproduction in any medium or format, as long as you give appropriate credit to the original author(s) and the source, provide a link to the Creative Commons license, and indicate if changes were made.
In cases where reviewers are anonymous, credit should be given to 'Anonymous Referee' and the source.
The images or other third party material in this Peer Review File are included in the article’s Creative Commons license, unless indicated otherwise in a credit line to the material. If material is not included in the article’s Creative Commons license and your intended use is not permitted by statutory regulation or exceeds the permitted use, you will need to obtain permission directly from the copyright holder.
To view a copy of this license, visit https://creativecommons.org/licenses/by/4.0/

Point-by-point Response to reviewers' comments

Reviewer #1 (Remarks to the Author):

Yi et al. report an analysis of the conformational changes that occur during the nucleotide addition cycle (NAC) of yeast RNA polymerase II (RNAPII). Their findings are made possible by recent advances in computational methods that sort cryo-EM particles into different conformational states and a clever approach to tether RNAPII transcription elongation complexes (ECs) to a cryo-EM grid in a way that enables diverse particle orientations. The authors use three different EC configurations to obtain ECs in different stages of the NAC: (1) a substrate free EC with an 8mer 3'deoxy RNA (5'–AUCGAGAG); (2) the same 3'deoxy EC incubated with ATP; and (3) the same EC containing 3'OH 8mer RNA allowed to react with NTPs. The authors assemble states observed in these three different populations into eight conformations that they connect as intermediates in the NAC, as well as sub-conformations like different stages of trigger loop folding. These eight states include some that have not been clearly observed previously, including the post-catalytic complex with bound pyrophosphate and an NTP-bound state that precedes NTP positioning in the catalytic pose. In addition, they define movements of two previously undefined structural modules in RNAPII in concert with steps in the NAC. Overall, the results will be of broad interest to researchers interested in transcription. The cryo-EM work appears to be expertly conducted and the method of tethering complexes to grids is an important advance. The work merits publication. However, there are a couple major limitations to the conclusions that currently are not acknowledged in the manuscript as well as points that require additional information or that require additional analysis. These concerns will need to be addressed in a revised manuscript before the manuscript can be judged to be appropriate for publication.

We thank the reviewer for positive and constructive comments. We have addressed reviewers' concerns below.

1. A major limitation of the results is that they are obtained with ECs lacking upstream RNA. A true EC contains a 10–11 bp RNA–DNA hybrid and single-stranded RNA in its RNA exit channel. The 8mer RNA used here will generate complexes that are more analogous to initially transcribing complexes (ITCs) that form when RNAPII begins synthesis from a promoter but has not yet transitioned to a fully formed EC. However, they are not true ITC mimics because they lack a 5'-triphosphate (as well as initiation factors like TFIIB). The lack of initiation factors is not a concern, but the use of an incomplete EC is a concern. First, it is unclear if the all of the motions described by the authors would occur in a mature EC. Second, the use of an RNA that does not fill the RNAPII cleft means that the authors cannot visualize the configuration of the upstream fork junction where RNA separates from DNA. The upstream fork junction functions in concert with the downstream fork junction where DNA is opened to insert the template DNA into the active site.

Thus, the scaffold chosen for the authors study introduces major limitations to their conclusions. It is possible that the clamp (module 1) and module 2 motions observed are specific to the pseudo-ITC used and would not occur in an EC that makes all upstream RNA contacts. Similarly, the upstream shift of the RNA–DNA hybrid observed in 'state I' may occur when the hybrid-binding cleft is not completely filled but may not be integral to the NAC in a mature EC. Finally, how do we know that the conformations reported for datasets 1 and 2 might be seen only when an artifactual 3'-deoxyRNA is present in the EC? These limitation must be clearly explained and addressed in the manuscript.

We thank the reviewer for raising this important point and apologize for the confusion caused by a typo error in the RNA sequence listed in the original Methods section. In fact, all complexes in this study were assembled using a **9-mer RNA** (instead of an 8-mer RNA in the submitted manuscript). The correct RNA sequence is as follows: [5′-AUC GAG AGG-3′]. We have corrected this information in the Methods section accordingly. To further clarify the experimental design, we have now included a schematic representation of the nucleic acid scaffolds used in all four cryo-EM datasets in **Fig. R1**.

For Dataset 2, the complex contains a 9-mer RNA primer together with an incoming ATP substrate, resulting in a nucleic acid configuration that approaches a 10-bp RNA-DNA hybrid region. Nevertheless, as correctly pointed out by the reviewer, the relatively short RNA in this scaffold does not fully recapitulate a complete elongation complex. In particular, the upstream fork junction—where the RNA strand separates from the DNA template strand—is not formed in our system.

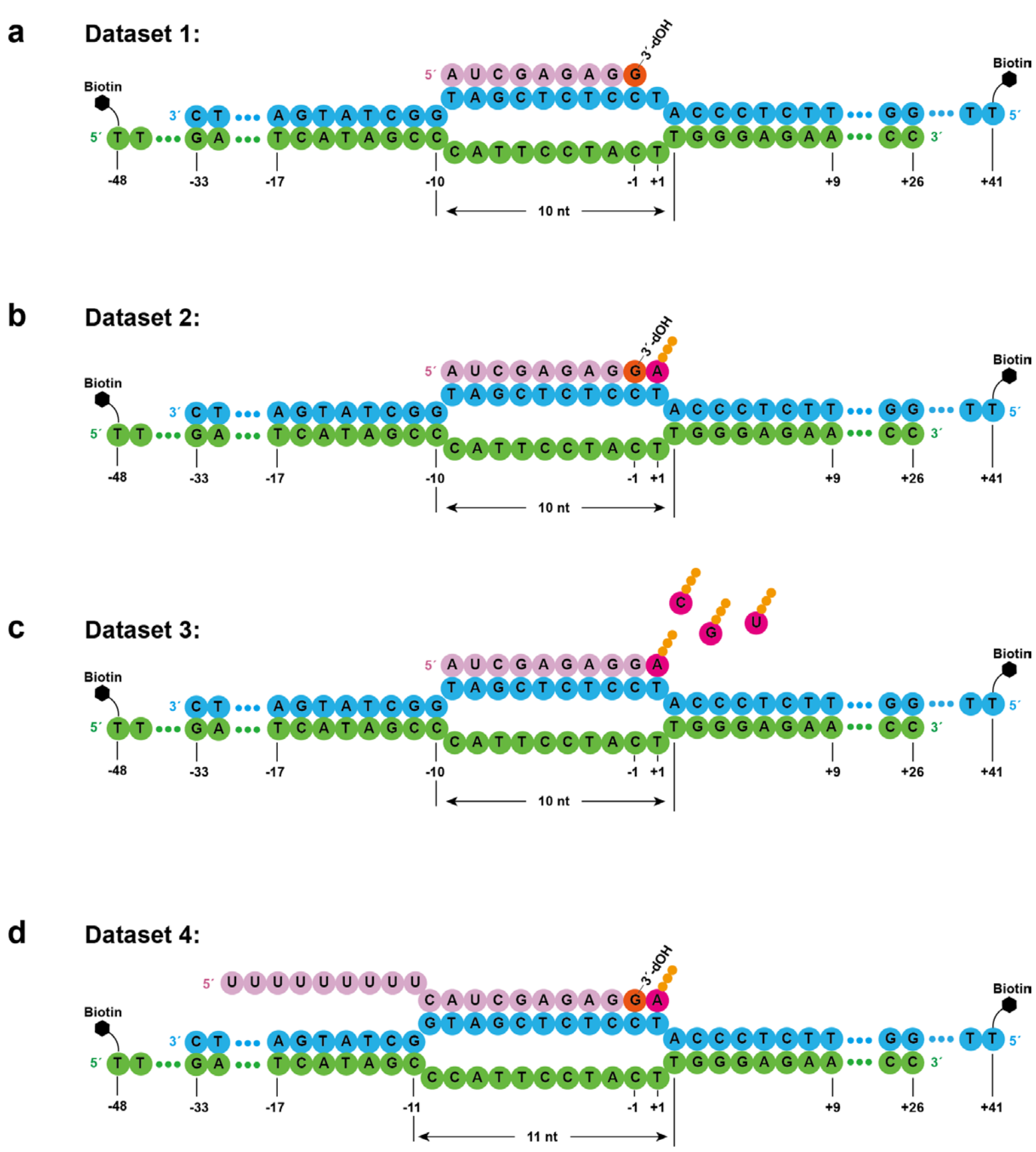

Figure R1. Nucleic acid scaffold design across four datasets

(a–d) Schematic representation of the nucleic acid scaffolds used in Dataset 1 (a), Dataset 2 (b), Dataset 3 (c), and Dataset 4 (d). RNA is shown in hot pink, template DNA in blue, and non-template DNA in green. In Datasets 1, 2 and 4, the 3′-terminal nucleotide of the RNA is a 3′-deoxy guanosine, highlighted in orange. Substrate ATP was included in Dataset 2 and 4, whereas all four NTPs (ATP, UTP, CTP, and GTP) were added in Dataset 3. Biotin was labeled at both ends of the scaffold for affinity grid capture during cryo-EM sample preparation.

To address the reviewer's concern regarding the use of a short RNA scaffold, we designed a mature EC with an extended 19-mer RNA (containing upstream RNA) and collected an additional cryo-EM dataset (New **Dataset 4**) (New **Extended Data Fig. 5**). This new scaffold supports formation of a complete RNA-DNA hybrid and allows visualization of the upstream RNA exit path. The new EC complex was assembled using the same strategy as in Dataset 2, including a 3′-dOH RNA primer and incorporation of an incoming ATP substrate. The detailed scaffold sequence for the new Dataset 4 is provided in **Fig. R1**. From this dataset, we determined two new structures corresponding to TL-closed and TL-open states. In the catalytic core, the TL-closed state closely resembles state V (frame 20) from Dataset 2, representing the fully closed, catalytically competent conformation, whereas the TL-open state aligns well with the substrate-bound state (frame 7) observed in Dataset 2.

We fully agree with the reviewer that the upstream and downstream fork junction function in a coordinated manner during nucleotide incorporation. Our new Dataset 4 directly addresses this point. We observe clear density for RNA extension beyond the hybrid region, with strand separation between RNA and tsDNA. The extended RNA is guided into the RNA exit channel. Notably, densities corresponding to four nucleotides of RNA extension (from i–10 to i–14) can be resolved in both structures (**Fig. R2a)**. The remaining upstream RNA is not well resolved, likely due to its exposure to bulk solvent and associated conformational flexibility.

Analysis of this new dataset reveals conformational dynamics that are highly consistent with those observed in Dataset 2 (**Fig. R2b–e**). Specifically, we performed structural comparisons of key dynamic elements across four RNAPII EC structures, including two long-RNA structures from Dataset 4 and two short-RNA structures from Dataset 2 **(Fig. R2b–d)**. Comparable conformational transitions are observed for multiple local elements, including funnel closure, BH bending, and clamp closure (i.e., coordinated rotation of module 1 and module 2) (**Fig. R2b–d**). To address the reviewer's concern regarding rotation of module 1 and module 2, we selected two representative domains that show the most pronounced local displacements, the clamp domain from module 1 and the lobe domain from module 2. These two domains are positioned on opposite sides of the downstream DNA tunnel and move toward each other during the transition from the TL-open to TL-closed state. The clamp closure in the long-RNA EC (Dataset 4) is fully consistent with that observed in Dataset 2 (**Fig. R2d**). In addition, similar reposition process of nucleic acid scaffold was observed in the long-RNA EC (**Fig. R2e; Fig. 4)**. Together, these results indicate that the conformational dynamics described in our study are consistent between a shortened RNA scaffold and an extended 19-mer RNA EC complexes. These new analyses have been incorporated into **Extended Data Fig. 10** and are described in the revised manuscript.

In addition, we obtained further supporting evidence by re-analyzing Dataset 3, which was assembled using a native RNA EC and all four substrate NTPs (ATP, UTP, CTP, and GTP). The results show that the RNAPII EC in Dataset 3 represents a mature elongation complex, with RNA extension, and undergoes conformational transitions comparable to those observed in Datasets 2 and 4 (**Fig. R3**). To verify RNA extension in

Dataset 3, we performed focused refinement with a local mask and visualized the density of the nascent RNA strand in the upstream region, where the RNA separates from the template DNA strand (**Fig. R3b**).

We have reprocessed Dataset 3, which contains a native RNA substrate, and resolved four distinct conformational states: II, III, VI, and VII (**Fig. R3a**). Thus, states II and III were observed in both ECs containing artifactual 3′-deoxyRNA and those containing native RNA. Notably, state III captured a substrate-bound intermediate, with the incoming NTP positioned in the active site. We also observed comparable conformational dynamics in Dataset 3, including the relative rotation between module 1 and module 2, as well as coordinated rearrangements of the funnel helix and folding of the trigger loop, consistent with the conformational dynamics observed in Dataset 2 (**Fig. R3**).

We appreciate the reviewer's insightful concern that state I identified in our study may arise from the use of a short RNA scaffold. Reprocessing of Dataset 3 did not yield a distinct state I population. For the new Dataset 4, which was limited in size, we could not reliably identify state I either. It is therefore possible that our experimental setup, including the short RNA scaffold and/or specific sequence context, stabilizes this transient intermediate state. We have revised the manuscript accordingly to include additional discussion of state I.

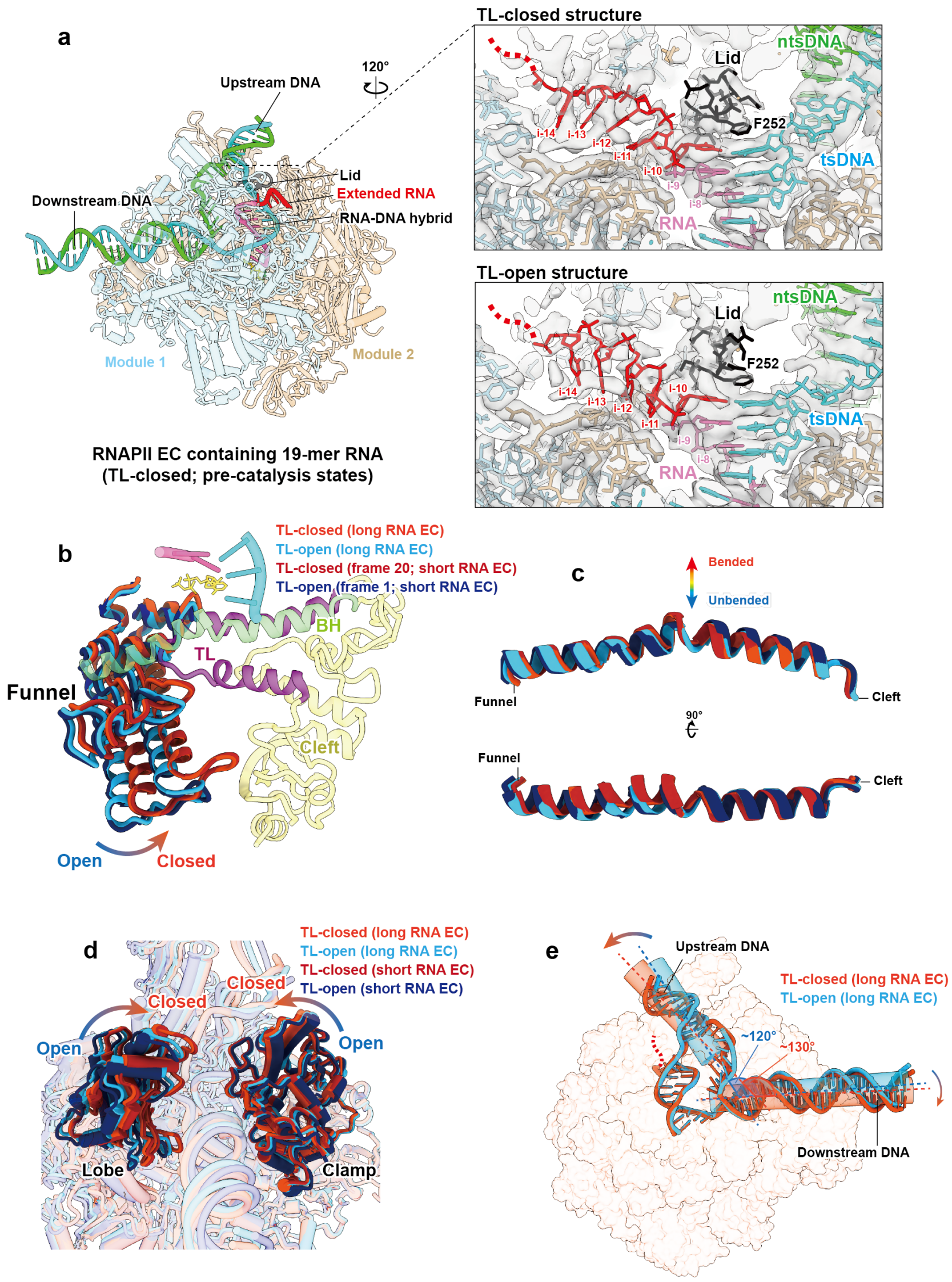

Figure R2. New cryo-EM structures of RNAPII EC containing long RNA (19-mer)

(a) Overall structure of the RNAPII EC assembled with an extended 19-mer RNA in the TL-closed state, representing a more complete elongation state compared to short-RNA complexes. The left panel shows the global architecture of the EC, with module 1 colored light blue and module 2 in tan. The nucleic acid scaffold includes tsDNA (cyan), ntsDNA (lime), and RNA (hot pink), with the extended upstream RNA highlighted in red. The right panel shows zoom-in views of the upstream RNA junction in two conformational states: TL-closed (upper) and TL-open (lower). Cryo-EM densities and corresponding atomic models illustrate RNA extension and strand separation from the tsDNA. The lid domain is colored black, with residue F252 highlighted.

(b–d) Structural comparisons of key dynamic elements across four RNAPII EC structures. The funnel domain (b), bridge helix (c), and lobe/clamp domain (d) are compared between long-RNA Dataset 4 (TL-closed, orange red; TL-open, sky blue) and short-RNA Dataset 2 (frame 20, firebrick; frame 1, navy). A continuous color gradient from blue (open) to red (closed) illustrates domain movements.

(e) Nucleic acid scaffold rearrangements accompanying EC tightening in the long-RNA dataset. Comparison of TL-open (sky blue) and TL-closed (orange red) states reveals coordinated clamp closure and global nucleic acid repositioning. Arrows indicate directional movements of upstream and downstream DNA. The axes of upstream and downstream DNA are represented by shaded cylinders (blue and red, respectively), highlighting changes in DNA trajectory.

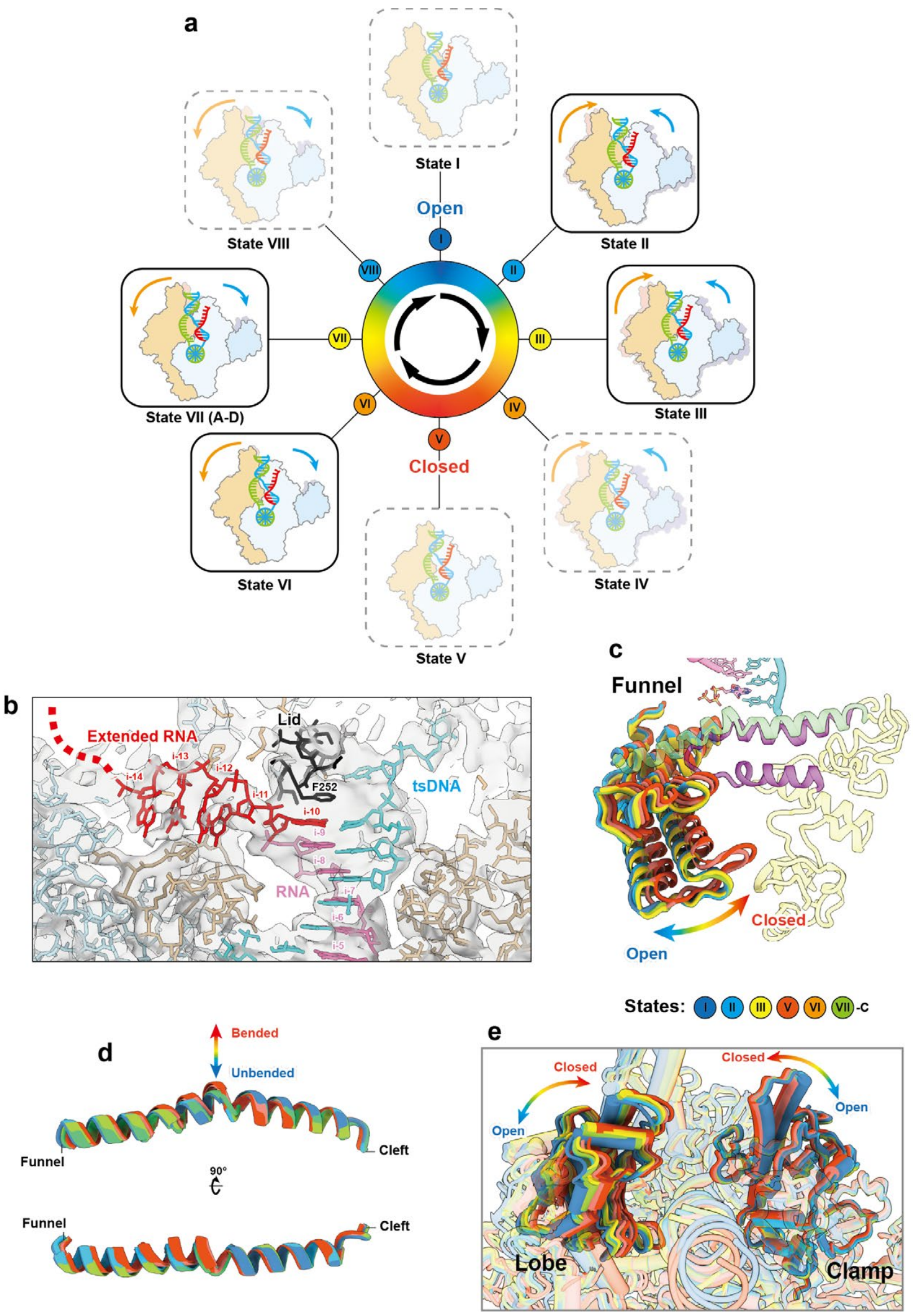

Figure R3. Conformational dynamics of RNAPII EC in the NTP dataset (Dataset 3)

(a) Schematic representation of the conformational spectrum of RNAPII elongation complex in Dataset 3. Eight states (state I–VIII) are arranged along a circular spectrum, transitioning from a clamp open conformation (blue) to a clamp closed conformation (red). Cartoon representations of RNAPII EC are shown for each state, with module 1 and module 2 colored in light blue and light orange, respectively. Arrows indicate the relative rotational movement between the two modules. States that were not resolved in the Dataset 3 are shown as transparent and outlined

by dashed lines, whereas states classified from Dataset 3 (state II, III, VI, and VII) are enclosed by solid outlines.

(b) Focused refinement of the upstream RNA-DNA hybrid junction from the Dataset 3. Cryo-EM density is shown as a transparent silver map overlaid with the atomic model. RNA is colored in hot pink, and template DNA (tsDNA) is shown in cyan. The lid domain, which separates the RNA strand from the DNA strand, is highlighted in black. The extended RNA from i–10 to i–14 was highlighted in red. A dashed red line extending from the RNA 5′ end denotes the continuation of the nascent RNA that is not resolved in the density, likely due to increased flexibility in the distal region.

(c–e) Structural comparison of local conformational changes across different states. Superposition of selected structures highlights conformational dynamics in the funnel helix (c), bridge helix (d), and lobe/clamp domain (e). Four states derived from the Dataset 3 (state II, III, VI, and VII) are compared together with reference states representing the extremes of the conformational spectrum from Dataset 2 (frame 1 at state I, most open; frame 20 at state V, most closed). Color coding is consistent with circular spectrum in panel a.

2. It's possible that the authors observe some of the movements and states described for the static complexes in their dataset of EC+NTPs (Dataset 3), but this question does not appear to be addressed specifically in the manuscript. Further, what is the state of these ECs under the conditions the data are collected? How many rounds of nucleotide addition have occurred? What are the positions of modules 1 and 2 in complexes VI–VIII and are motions of modules 1 and 2 seen in this dataset? Were complexes I–V seen in this dataset? If multiple rounds of nucleotide addition have occurred in Dataset 3, what configurations of the upstream fork junction are seen? All of these questions need to be considered and addressed in a revised manuscript.

We thank the reviewer for these insightful questions regarding the conformational states and biochemical context of the NTP dataset (Dataset 3). We agree that a detailed analysis of these states, and their relationship to those observed in ATP dataset (Dataset 2), are important.

First, we confirm that comparable conformational states and motions are indeed observed in Dataset 3. Specifically, we classified four distinct states, including state II, III, VI, and VII (**Fig. R3**). Notably, both states II and III correspond closely to states identified in Dataset 2. While the post-catalytic states (state VI and VII) are unique in Dataset 3. Furthermore, the relative rotation between module 1 and module 2 observed in Dataset 3 follows the same trend as in Dataset 2, supporting consistent conformational dynamics across the two datasets (**Fig. R3**).

We further include more detailed description of both the substrate-gradient grid strategy (**Extended Data Fig. 3**) and the transcription assay used to analyze these conditions (**Extended Data Fig. 4**). For the substrate-gradient grid approach, biotin-tagged ECs were immobilized on streptavidin-coated grids, and unbound complexes were washed away. Subsequently, a small droplet (0.2 µL) of highly concentrated NTP mixture (10 mM) was then applied to the top surface of the sample. NTP diffusion generated a transient concentration gradient immediately before blotting and plunge-freezing, enabling capture

of a broader range of potentially transient intermediate transcriptional states of ECs (**Extended Data Fig. 3**). See response to Minor Point 6 for more details.

To estimate the extent of nucleotide addition under these conditions, we performed transcription assays using a range of NTP concentrations and reaction times to model the substrate-gradient setup. The results indicate that the majority of ECs extend by approximately 10 nucleotides (major pausing site within the time frame of vitrification) (**Extended Data Fig. 4**). We reprocessed the Dataset 3, focusing on local refinement of the RNA-DNA junction. The local-refined map reveals a clear density for the nascent RNA extending beyond the RNA-DNA junction (**Fig. R3b**). Density within the RNA exit tunnel corresponds to at least five nucleotides (from i–10 to i–14) (**Fig. R3b**), while further upstream RNA becomes increasingly flexible and is not well resolved. We have revised the manuscript accordingly.

3. The authors describe the conformational changes they detect using a causal framework. For example, they suggest funnel closure drives the rotation of module 2 and that bridge helix bending drive movement of module 1 (lines 271–272). These statements imply that the funnel or bridge helix movements occur first and then cause the movements of modules 1 and 2. It's unclear that causal relationships can be discerned from the authors datasets. In general, thermal fluctuations cause RNAPII to assume different conformational states but these effects of thermal energy don't necessarily operate specifically on one component of RNAPII that transmits an effect to another component. Rather, thermal energy can cause conformational changes via impacts on fluctuations of any component, which are linked to movements of other components through a complex network of interactions. Unless the authors can describe a rationale for the causal relationships they suggest, they should reframe these descriptions of movement as being linked rather than being driven in one direction.

We thank the reviewer for this insightful comment. We fully agree that our previous wording may have implied a directional, mechanically propagated sequence of movements (e.g., a cascade-like model in the original version of **Extended Data Fig. 11**), which lacks direct support from our cryo-EM data.

Considering the reviewer's suggestion, we carefully re-examined our structural results. Our analysis suggests that conformational transitions among individual structural elements occur in a coordinated manner. We have revised the related section, removed original **Extended Data Fig. 11,** and added a new panel shown in **Fig. R4a** to the revised **Extended Data Fig. 9**, in which conformational changes of individual RNAPII elements are mapped onto the structure to emphasize their spatial organization.

We agree with the reviewer that these conformational transitions could be governed by thermal fluctuations within an interaction network of RNAPII. We have thus revised the manuscript throughout to replace causal terms such as "drives," "induces," and "cascade" with more appropriate descriptions, including "coupled," "coordinated," and "associated."

We have also refined our description of the conformational dynamics observed in this study. We now describe this process as the **"catalysis-associated conformational**

dynamics of the RNAPII EC", referring to the global conformational behavior of the elongation complex. This process can be broadly divided into two phases: "**substrate-induced RNAPII tightening"** and **"post-catalysis RNAPII relaxation"**. Upon substrate binding at the active site, multiple structural elements—including TL folding, funnel closure, and BH bending—are observed in conformations associated with clamp closure (module 1/2 rotation). These changes span from the active-site region to the overall architecture of the elongation complex. We now describe these changes as coordinated conformational states.

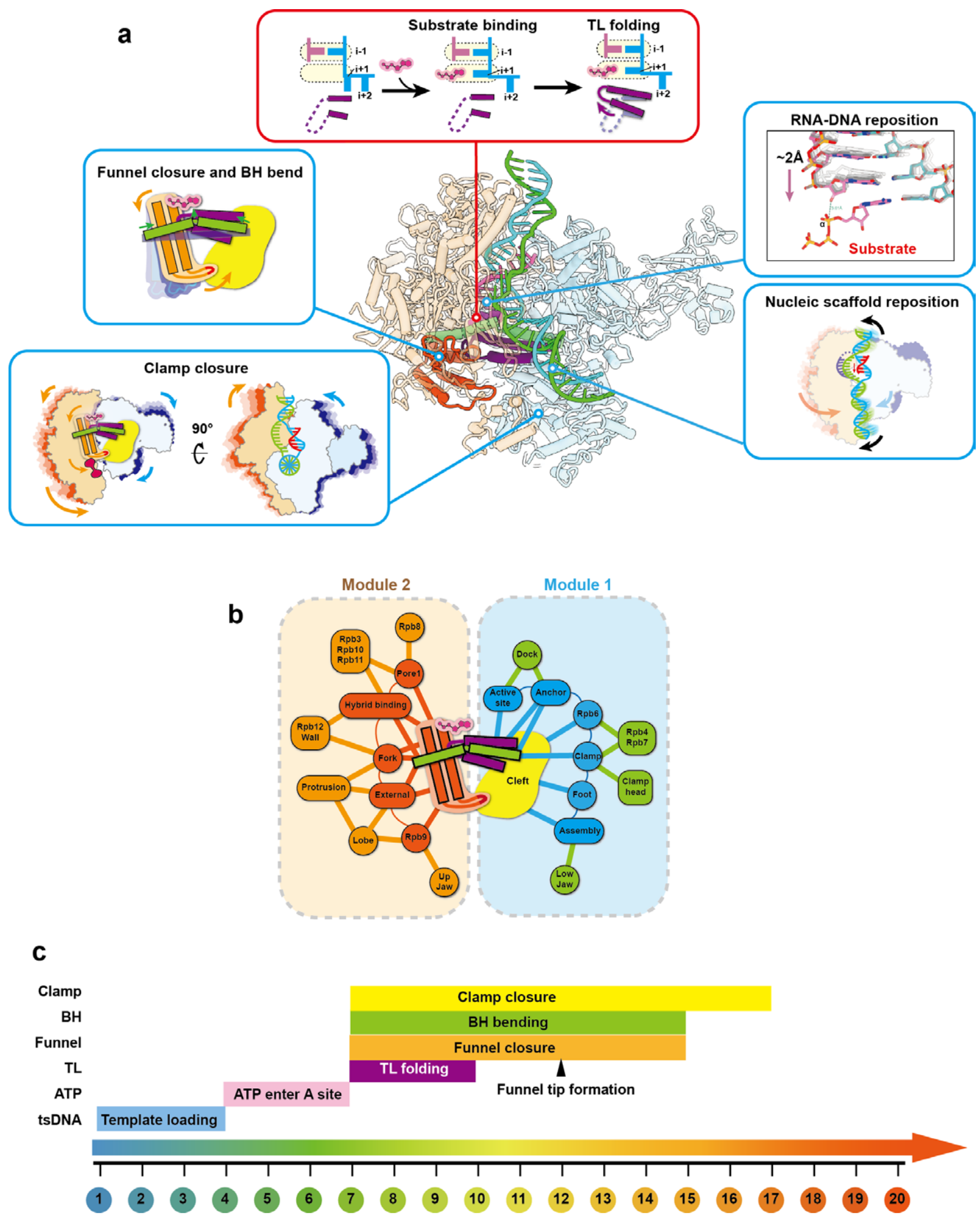

Figure R4. Catalysis-associated conformational dynamics of RNAPII EC

(a) Schematic representation of coordinated conformational changes in RNAPII during catalysis. A central RNAPII structure is shown with key structural elements highlighted, including the substrate (magenta), TL (purple), BH (light green), funnel (orange red), module 1 (light blue) and module 2 (tan). Surrounding cartoon diagrams illustrate the conformational changes of individual elements. Each schematic is connected to its corresponding structural location by lines, emphasizing spatial mapping rather than causal relationships. Previously reported conformational changes are indicated by red boxes, whereas newly identified changes in this study are highlighted in blue boxes.

(b) Interaction network underlying RNAPII conformational dynamics. The network is centered on the catalytic region, including the substrate, TL, funnel, BH and Cleft. RNAPII is divided into two modules, module 1 and 2. First-layer interactions are highlighted within module 2 (dark orange) and module 1 (sky blue), representing elements directly connected to the core region. Peripheral interactions are further indicated in module 2 (light orange) and module 1 (light green), illustrating an extended interaction network.

(c) Conformational dynamics spectrum derived from the Dataset 2. A series of 20 structural frames (states I–V and intermediates) are arranged to represent the continuous transition from an open to a tightened RNAPII conformation. Key conformational dynamic events are mapped onto this spectrum. The intervals highlighted for each structural element correspond to the frames in which the most pronounced conformational changes occur. These assignments are based on comparative analyses of local structural deviations between frames. Importantly, the indicated intervals represent regions of dominant structural rearrangement rather than the full extent of conformational variation. Structural elements may continue to undergo more subtle adjustments outside these intervals, including side-chain rearrangements or local density differences, which are not explicitly highlighted in this representation.

4. What is the relationship between the module 1 – module 2 rotations observed in RNAPII and the swiveling motions observed in bacterial RNAP (e.g., Zhu et al. 2022 Nat Commun 13:1546 doi:10.1038/s41467-022-29148-0). I'm pretty sure they are not similar, although rotations of the clamp domain appear to be involved in both. The point of comparing the motions in RNAPII and bacterial RNAP is not to suggest that they are the same, although similarities should be noted. The point is to put the results in a broader evolutionary context. How generalizable are the module 1 – module 2 motions likely to be? Are they also likely to occur in archaeal RNAP, RNAPI, or RNAPIII?

We thank the reviewer for raising this important point. We have made comparison between the module 1 – module 2 rotations observed in RNAPII and the swiveling motions observed in bacterial RNAP. In our view, these represent mechanistically distinct conformational dynamics, although both involve movements of clamp-associated regions.

In bacterial RNAP, swiveling has been reported in the context of transcriptional pausing and translocation-related intermediates. Structural studies suggest that swiveling is an intrinsically accessible conformational mode of bacterial RNAP that becomes preferentially stabilized under pause-associated conditions[1,2]. Accordingly, the swiveled conformation is best understood as a translocation-coupled, pause-associated state rather than a substrate- or catalysis-induced motion (**Figure R5a**). Previous cryo-EM studies have captured swiveled *E. coli* elongation complexes under pausing conditions induced by specific sequences or elongation factors. For example, an upstream RNA hairpin (*his*PEC) traps the elongation complex in a half-translocated state and is accompanied by a swiveled conformation[1]. In addition, Zhu et al. showed that NusA shifts the equilibrium toward more extensively swiveled states[2].

By contrast, the clamp closure that we observe in eukaryotic RNAPII is detected specifically upon substrate binding (**Fig. R5a**). In our cryo-EM datasets, this motion is reproducibly observed in the presence of substrate (Datasets 2 to 4), but not in its absence (Dataset 1). We therefore interpret this movement as a substrate-induced clamp

closure. In this process, substrate binding in the active site promotes TL folding, which is coordinated with a global conformational rearrangement of the polymerase. As the substrate is engaged, modules 1 and 2 move toward the downstream DNA channel, tightening the grip on the nucleic-acid scaffold and promoting a pre-catalytic configuration (state V). Following nucleotide incorporation, the polymerase undergoes a reverse transition from a closed to a more open conformation, coordinated with TL unfolding. This transition reaches the most open conformation at state I, in which the interaction with the nucleic-acid scaffold is relatively loosened, thereby facilitating translocation (**Fig. R5a**). Thus, the clamp closure described here is mechanistically distinct from the swiveling observed in bacterial RNAP.

From a structural perspective, the swivel module described in bacterial RNAP shows substantial spatial overlap with module 1 in RNAPII. Despite this spatial similarity, the modes of motion are distinct. In bacterial RNAP, swiveling involves a rotation of the swivel module approximately parallel to the plane of the nucleic acid scaffold (**Fig. R5b**). In contrast, the motions we observe in RNAPII involve coordinated movements of module 1 and module 2 toward the nucleic acid scaffold, analogous to the closing of a clamp. In this process, module 1 and module 2, which can be viewed as the two halves of a pincer-like architecture, move toward each other and toward the downstream DNA, thereby tightening the nucleic acid cleft (**Fig. R5c**). Thus, whereas bacterial swiveling reflects a planar rotational motion, the RNAPII closure (module 1/2 rotation) represents a convergent, cleft-narrowing movement that directly modulates engagement with the nucleic acid scaffold. We have incorporated the comparison in revised Discussion Section and new **Extended Data Fig. 12**.

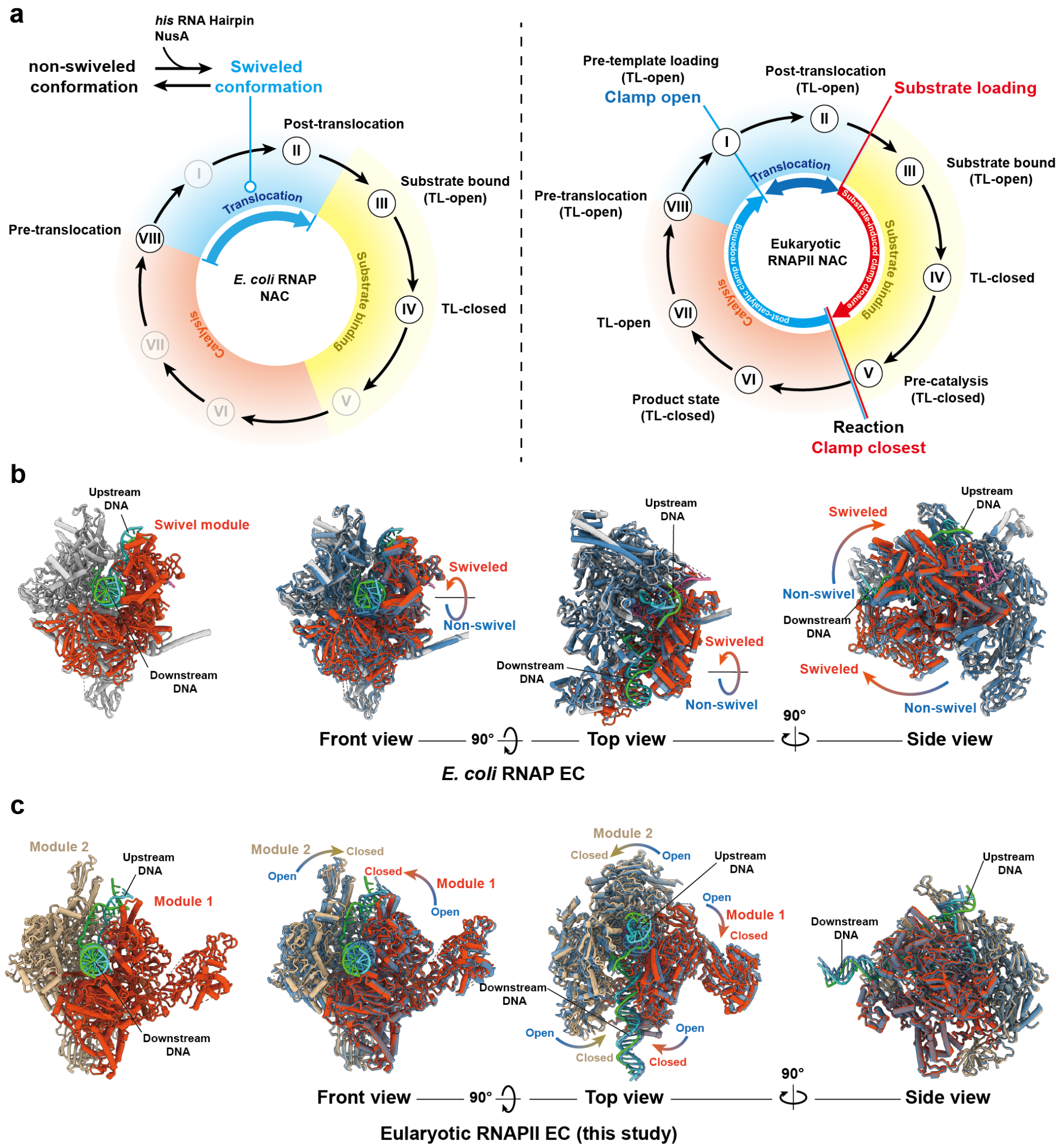

Figure R5. Comparison between bacterial RNAP swiveling and eukaryotic RNAPII catalysis-dependent clamp dynamics

(a) Schematic representation of the nucleotide addition cycle (NAC) in bacterial RNA polymerase (left) and eukaryotic RNA polymerase II (right), with conformational transitions mapped onto distinct stages of the cycle. The NAC is divided into three major phases: translocation (blue), substrate binding (yellow), and catalysis (orange). Roman numerals indicate structurally defined intermediate states. Eight states are resolved for RNAPII, whereas only a subset of states has been structurally characterized in bacterial RNAP; unresolved states are depicted in semi-transparent shading. In bacterial RNAP (left), swiveling is coordinated with the translocation phase of the NAC. This conformational equilibrium can be modulated by transcription factors (e.g., NusA[2]) or pausing signals such as *his* RNA hairpins[1], which bias the enzyme between non-swiveled and swiveled states. In contrast, eukaryotic RNAPII (right) exhibits a distinct mode. Substrate binding promotes clamp closure (red arrow), progressing toward a maximally closed state near the catalytic step. Following catalysis, the clamp reopens (blue arrow), returning toward a more open conformation. The timing of substrate loading, as well as the most open and most closed clamp conformations, are indicated along the cycle.

(b–c) Structural comparison of bacterial RNAP swiveling and eukaryotic RNAPII clamp dynamics.

(b) Bacterial RNAP swiveling. The leftmost panel shows the cryo-EM structure of *E. coli* RNAP in the swiveled state (PDB: 6ASX[1]). The swiveling module (orange red) partially overlaps with module 1 in eukaryotic RNAPII. Three orthogonal views (front, top, and side) depict the reversible transition between the non-swiveled (PDB: 6BJS[1]) and swiveled states, with arrows indicating the direction of motion. (c) Eukaryotic RNAPII clamp closure. By contrast, the leftmost panel shows the cryo-EM structure of the RNAPII elongation complex (EC) in the pre-catalytic state (state V) determined in this study. Two major structural modules are highlighted: module 1 (orange red) and module 2 (light blue). Three orthogonal views (front, top, and side) illustrate the coordinated, progressive movement of these modules during clamp dynamics, with gradient arrows indicating the transition from the open (state I; pre-template loading) to the closed (state V; pre-catalysis) conformation.

Minor points

1. The authors use Pol II to refer to RNAPII throughout the manuscript. Pol II is ambiguous because it does not distinguish DNA and RNA polymerases. RNAPII is a preferable abbreviation.

Thanks for reviewer's suggestion. We have replaced the "Pol II" with "RNAPII".

2. The scaffold sequence(s) need to be shown in the figures (either in Figure 1 or in Extended Data Figures S1–S3) drawn in a way to show the upstream and downstream DNA duplexes, the transcription bubble, and the RNA–DNA hybrid.

Thanks for reviewer's suggestion. New panels for the scaffold design were added in the **Extended Data Fig. 1, 2, 3, and 5**.

3. The manuscript must identify S. cerevisiae as the source of RNAPII either in the abstract or the first para of the introduction. As currently presented, the reader is left in the dark about whether the paper addresses mammalian or yeast RNAPII, even though specific Rpb1 residues are mentioned without context in the first paragraph of the introduction.

Thanks reviewer's suggestion, source species of "*S. cerevisiae*" has added in both abstract and introduction.

4. line 130. The authors state that spontaneous oscillation of RNAPII is known, but the citation given is to bacterial RNAP.

We thank the reviewer for this careful examination of our citations. We have now included additional references from studies on both *E.coli* RNAP and eukaryotic RNAPII[3–10] that support the view that translocation is primarily driven by thermal fluctuations and can be

described by a Brownian ratchet mechanism. These references have been incorporated into the revised manuscript.

5. line 391 This conclusion that upstream and downstream translocation are not coupled is an example of a conclusion that may incorrect if it depends on 3'deoxyECs with less than complete RNA–DNA hybrids. Unless these states have been observed in Dataset 3 with complete hybrids, this caveat should be stated explicitly in the revised manuscript.

We thank the reviewer for this insightful comment. We re-analyzed Dataset 3 and further identified states II and III, which are consistent with Dataset 1 and 2. We note that state I represents a highly transient intermediate conformation, which is intrinsically difficult to capture. Analysis of Dataset 3 did not yield a distinct state I population. This is likely due to the transient nature of this state, which makes it challenging to capture in the dataset containing more native RNA scaffolds. It is possible that our experimental setting (such as a short RNA and/or specific sequence) stabilizes this short-life state. We have revised the manuscript accordingly to include new discussion about state I.

6. What are the conditions used to generate complex 3 (active NTP addition)? In the results, the authors mention a 'substrate-gradient strategy' but don't explain what the strategy consists of (line 314). It's unclear which NTPs are present (all 4?), for how long, and at what temperature for the formation of the complex. The exact conditions used to generate Dataset 3 must be given in the methods. The 'substrate-gradient strategy' must be explained in the results and the state of the ECs in control reactions must be documented in a figure. What is the distribution of RNA lengths in reactions that precisely mimic the cryo-EM data collection?

We thank the reviewer for the careful evaluation. We now include more detailed description in Method Section (below).

Biotin-tagged RNAPII ECs were first applied to streptavidin-coated affinity grids and incubated for 5 minutes to allow stable immobilization. Unbound ECs were subsequently removed by washing twice gently with 50 µL of fresh buffer, and excess buffer was gently absorbed from the edge of the grid using filter paper. The grid was then transferred to a Thermo Fisher Vitrobot IV equilibrated at 4 °C and 100% humidity. Before blotting, 3 µL of fresh buffer was added to the grid surface, followed by application of a small droplet (0.2 µL) of a highly concentrated NTP mixture (10 mM total, containing all four NTPs: ATP, UTP, CTP, and GTP) from the upper edge of grid. The grid was immediately blotted for 1 second and plunge frozen. During this brief interval, NTPs diffuse and generate a transient concentration gradient across the grid (**Extended Data Fig. 3**). Sampling across this NTP concentration gradient enables capture of a range of potentially transient intermediate transcriptional states of ECs within the time frame of vitrification (**Extended Data Fig. 3**). We also performed transcription assays to model this substrate-gradient setup by varying NTP concentrations and reaction times (**Extended Data Fig. 4**). These experiments show that the majority of ECs extend by approximately ~10 nucleotides (major pausing site within seconds time scale), providing an estimate of RNA length for

nucleic acid modeling. Cryo-EM analysis of Dataset 3 reveals density corresponding to partially extended nascent RNA in the exit tunnel (**Fig. R3b**), supporting ongoing RNAPII elongation.

7. If I am interpreting the figures correctly, the +1 DNA nucleotide is colored inconsistently in Figure 1 panels C and D. Why not color it yellow in all the panels to avoid confusion?

Thanks for reviewer's suggestion, we have revised the color code to make it consistent.

8. Are the template nucleotides in Figure 1 panel E misnumbered? At a minimum the use of colors and numbering is confusing. The yellow highlighted nucleotide in panel C is labeled +1 whereas the yellow highlighted nucleotide in panel E is labeled –1. The authors should check the numbering carefully and also think about ways to make the figures easier to follow.

We thank the reviewer for carefully noting this issue. This labeling error has now been corrected in the revised figure. In addition, we have revised the color scheme and labeling to improve clarity and ensure consistent assignment of nucleotide positions across panels.

Reviewer #2 (Remarks to the Author):

Yi, Li et al. report new cryo-EM structures that elucidate additional steps of the RNA polymerase II nucleotide addition. Overall, this is an important and exciting contribution to the field and facilitates our understanding of one of the most basic processes in molecular biology. The manuscript is well-written and the figures are especially commendable. These structures will significantly improve our understanding of the nucleotide addition cycle. This reviewer only has a few points that should be addressed by the authors.

Major points

1) The title "A complete structural pathway…" is misleading. The authors simply cannot know if the observed states COMPLETELY capture the structural pathway for nucleotide addition. Perhaps "An expanded structural pathway…" is a better choice for the title.

Reviewer's comment regarding to "complete" is well-taken. We revise our title to emphasize structural dynamics of NAC.

2) The authors use terms like "near-atomic" or "high" resolution throughout the manuscript. This terminology is highly misleading as the provided structures are only in the realm of 3-4 Å. These resolutions are not even close to being "near atomic"/"high" in nature. Therefore, the authors must refrain from using these terms as they suggest a higher map quality than is available from the here provided data.

We thank the reviewer for this comment. We have removed these terms throughout the manuscript.

3) With their limited resolution, how are the authors certain about metal assignments?

We thank the reviewer for this important question regarding metal ion assignment. In our analysis, assignments of metal are primarily based on the presence of corresponding cryo-EM densities at the well-established metal-binding sites in the RNAPII EC, including the two catalytic Mg^{2+} ions in the active site and eight conserved Zn^{2+} ion clusters. In cases where the local density is less well defined, our assignments were further guided by our ~2 Å RNAPII EC structures, including a pre-catalytic state (1.96 Å; PDB: 9SV6) and a post-catalytic state (2.33 Å: PDB: 9RYB), in which metal coordination is clearly resolved[11]. These structures (collected at the same condition) provide a reliable framework for validating metal positions in the current dataset.

4) FSC curves and angular distribution plots are lacking.

We thank the reviewer for the reminder. The FSC curves and angular distribution plots have now been included in **Extended Data Figures 1, 2, 3, and 5**, and additionally provided as source data.

Minor points

1) Resolution is incorrectly reported to two significant digits beyond the decimal point. I doubt that resolution can be reported this accurately. I suggest the authors only report to a single digit, e.g. 3.0 Å, instead of 2.98 Å.

We thank the reviewer for the suggestion. We now report resolutions to a single digit.

Reviewer #3 (Remarks to the Author):

Yi, Li et al., present cryo-EM structures of RNA polymerase II over the course of its nucleotide addition cycle. Modern cryo-EM classification approaches as well as clever biochemistry allow for a complete trajectory of the NAC that was previously not possible with X-ray crystallography. This heroic effort captures multiple states that have not been perviously observed, and shows how active site conformations influence the overall conformation of the enzyme. This paper will serve as a critical reference to anyone studying RNA polymerase dynamics and elongation, and will be a model for how to follow a complete enzymatic process using cryo-EM. I have a few comments that the authors should address prior to publication.

-Figure 4 shows many of the changes in RNA polymerase II over the course of the nucleotide addition cycle. The upstream DNA is typically quite flexible, and it would be helpful if the upstream DNA would be discussed/shown in this figure or in the extended data. The Rpb4/7 stalk is also typically quite flexible (and this appears to be the case here too, given the provided 2D classes). It would be helpful to more explicitly describe any potential Rpb4/7 fixed state associated with a particular stage of the nucleotide addition cycle.

We thank the reviewer for this helpful suggestion. We have performed additional analyses on the upstream DNA and the Rpb4/7 stalk with results shown in **Fig. R6** below (also included new panels in **Fig. 4** and **Extended Data Fig. 8)** to better illustrate their conformational dynamics across the nucleotide addition cycle.

For the Rpb4/7 stalk, our analysis indicates that its motion is coordinated with that of module 1. Examination of the Dataset 2 shows that module 1 undergoes a gradual movement toward the downstream DNA. The Rpb4/7 stalk follows this motion in a coordinated manner (**Fig. R6a–b**). Quantitatively, the observed rotation is approximately ~2°, with an overall displacement of ~4 Å, consistent with the motion of module 1. We also note in a few intermediate states, the Rpb4/7 stalk is too flexible to be modeled. These observations suggest that Rpb4/7 does not adopt a distinct fixed conformation at a specific stage, but instead exhibits continuous, coordinated movement with module 1.

For the upstream DNA, we note that only the most open and most closed states were shown in the original manuscript (**Fig. 4**). During revision, we re-examined intermediate frames across the conformational spectrum. The upstream DNA exhibits substantial positional and orientational changes that are coordinated with movements of module 2. Across the trajectory, the upstream DNA shows an overall displacement of approximately ~16 Å and a rotation of ~40°, indicating a large motion (**Fig. R6c; Fig. 4e**).

In the interaction context, the upstream DNA is directly contacted by the protrusion domain, wall domain, and Rpb12 subunit, all of which display coordinated movements during the conformational dynamics. Importantly, the motion of the upstream DNA should not be considered in isolation, but rather from a global perspective of the entire nucleic acid scaffold. In this context, the observed rearrangements reflect a coordinated, system-

wide conformational transition of the RNAPII EC. Upon substrate loading, RNAPII undergoes a global conformational change characterized by rotation of module 1 and module 2, which we define as clamp closure (**Fig. R6d**). These two modules, analogous to the two arms of a pincer, move toward each other along the downstream DNA tunnel, thereby tightening their grip on the nucleic acid scaffold. As a result, the nucleic acid scaffold undergoes a global rearrangement (**Fig. R6d**). A representative feature of this transition is illustrated in the left panel of **Fig. R6d**, where key structural elements lining the nucleic acid tunnel—including the protrusion, lobe, and clamp regions—move inward, collectively narrowing the tunnel. Functionally, this "tight" state is likely favorable for nucleotide addition, as it enhances substrate positioning and promotes catalytic accuracy.

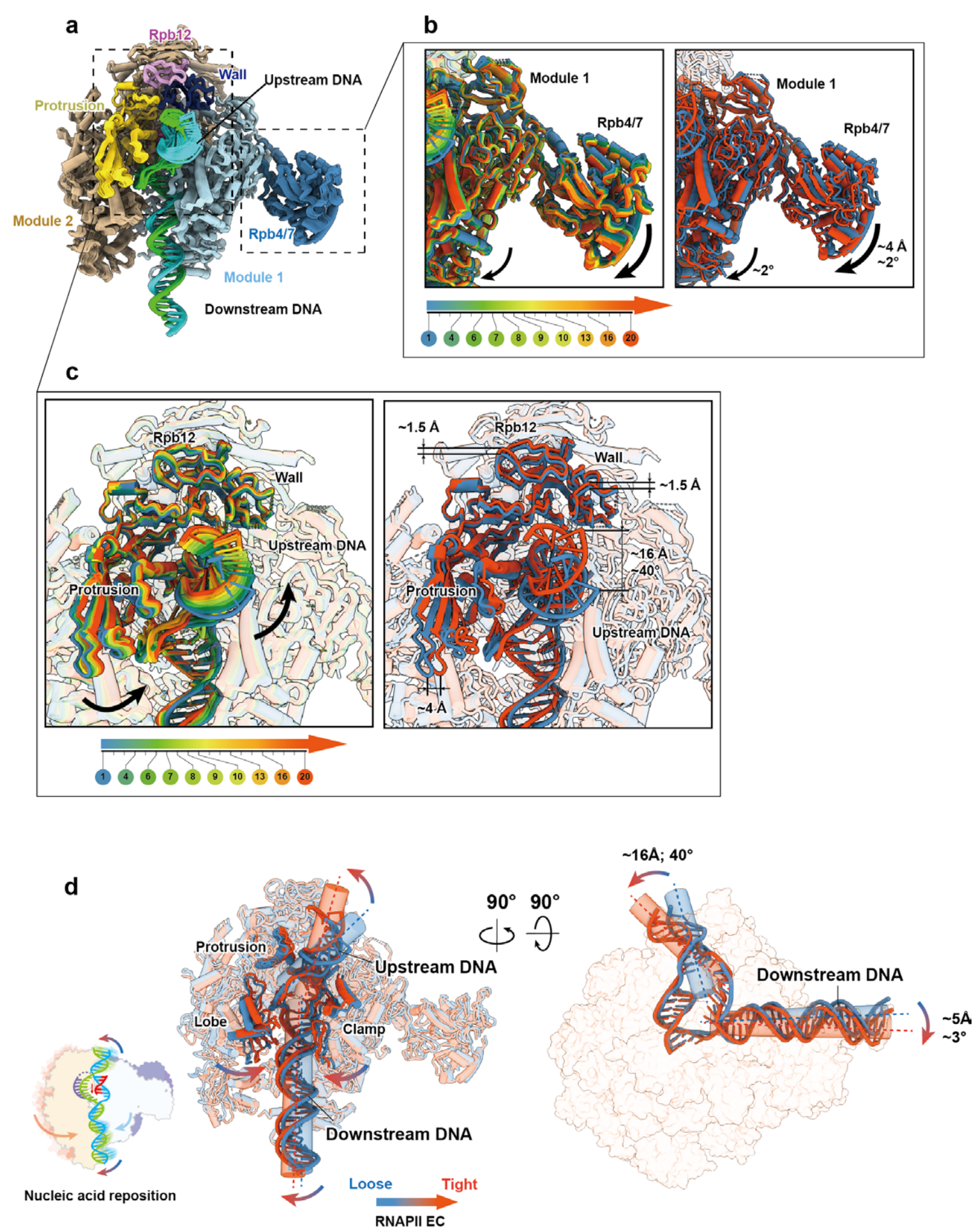

Figure R6. Conformational dynamics of the Rpb4/7 stalk and upstream DNA

(a) Top view of RNAPII showing conformational dynamics across selected frames from the Dataset 2. Multiple structural frames (frames 1, 4, 6, 7, 8, 9, 10, 13, 16, and 20) are superimposed to illustrate the overall range of motion during the nucleotide addition cycle. Structural elements are color-coded as follows: module 1 (light blue), module 2 (tan), protrusion domain (gold), wall domain (navy), and Rpb12 (plum).

(b–c) Close-up views of the Rpb4/7 stalk (**b**) and upstream DNA (**c**) region. Each left panel shows the superposition of multiple frames, illustrating the gradual and continuous movement of the Rpb4/7 stalk or upstream DNA during the conformational transition. Each right panel compares the most open (frame 1, state I) and closest (frame 20, state V) states. The direction of motion is indicated by black arrows, and the corresponding displacement and rotation are annotated accordingly.

(d) Nucleic acid scaffold repositioning during RNAPII EC tightening. Structures of state I (frame 1; steel blue) and state V (frame 20; orange red) from Dataset 2 are compared. Selected elements lining the nucleic acid tunnel, including the protrusion, lobe, and clamp domains, are highlighted to illustrate tunnel narrowing. Upstream and downstream DNA trajectories are represented by shaded cylinders, with gradient-colored arrows (blue to red) indicating the direction of movement from the loose to tight state. In the left corner, the cartoon schematic summarizes nucleic acid scaffold repositioning.

-Increase font size of the scale bars in Extended Data Figures 1-3 for the 2D classes.

We thank the reviewer for this observation. We have revised **Extended Data Figures 1-3** accordingly.

-2D classes do not show lots of high resolution features. Is this a limitation of the datasets or how it rendered in the figure? There also appears to be contamination in some of the images (eg Extended Figure 1c top left class has a very bright spot and the particles do not appear to be well-aligned). Given the limited number of 2D class images provided, I would recommend that the authors select ones that show high resolution features and were presumably used for downstream map generation.

We thank the reviewer for the careful examination of the 2D class averages. In the revised manuscript, we have replaced these images with a new set of representative 2D class averages that better illustrate structural details. Regarding the bright feature observed in some classes, this does not arise from contamination. Instead, it corresponds to the nucleic acid scaffold within the RNAPII elongation complex. Due to the relatively strong electron scattering of the nucleic acid backbone, this region can appear as a high-intensity signal in 2D class averages, consistent with a top-down view of the downstream dsDNA.

-It would help if the authors provided a graphical representation of the nucleic acid scaffold used for structure determination in an Extended Data figure.

We thank the reviewer for this helpful suggestion. In response, we have generated new figure panels illustrating the nucleic acid scaffold design in **Extended Data Figs. 1, 2, 3, and 5** (also see **Fig. R1**).

-For all deposited cryo-EM maps, FSC plots (ideally 3D FSC) and 3D angular distribution plots need to be provided. I do not see these in the extended data.

We thank the reviewer for the reminder. The FSC curves and angular distribution plots are now provided in **Extended Data Figures 1, 2, 3, and 5**, and included in source data.

Reference

1. Kang, J. Y. *et al.* RNA Polymerase Accommodates a Pause RNA Hairpin by Global Conformational Rearrangements that Prolong Pausing. *Molecular Cell* **69**, 802-815.e5 (2018).
2. Zhu, C. *et al.* Transcription factors modulate RNA polymerase conformational equilibrium. *Nat Commun* **13**, 1546 (2022).
3. Komissarova, N. & Kashlev, M. RNA polymerase switches between inactivated and activated states By translocating back and forth along the DNA and the RNA. *J Biol Chem* **272**, 15329–15338 (1997).
4. Dangkulwanich, M. *et al.* Complete dissection of transcription elongation reveals slow translocation of RNA polymerase II in a linear ratchet mechanism. *eLife* **2**, e00971 (2013).
5. Brueckner, F. & Cramer, P. Structural basis of transcription inhibition by α-amanitin and implications for RNA polymerase II translocation. *Nat Struct Mol Biol* **15**, 811–818 (2008).
6. Shaevitz, J. W., Abbondanzieri, E. A., Landick, R. & Block, S. M. Backtracking by single RNA polymerase molecules observed at near-base-pair resolution. *Nature* **426**, 684–7 (2003).
7. Palangat, M. & Landick, R. Roles of RNA:DNA hybrid stability, RNA structure, and active site conformation in pausing by human RNA polymerase II. *Journal of molecular biology* **311**, 265–82 (2001).
8. Malinen, A. M. *et al.* Active site opening and closure control translocation of multisubunit RNA polymerase. *Nucleic acids research* **40**, 7442–51 (2012).
9. Galburt, E. A. *et al.* Backtracking determines the force sensitivity of RNAP II in a factor-dependent manner. *Nature* **446**, 820–3 (2007).

10. Bar-Nahum, G. *et al.* A ratchet mechanism of transcription elongation and its control. *Cell* **120**, 183–93 (2005).

11. Li, Q. *et al.* Sub-2 Å cryo-EM structures of transcribing RNA polymerase II reveal critical roles of water molecules in catalysis. *Molecular Cell* https://doi.org/10.1016/j.molcel.2026.04.007 (2026) doi:10.1016/j.molcel.2026.04.007.

REVIEWERS' COMMENTS

Reviewer #1 (Remarks to the Author)

The revised manuscript from Yi et al. addresses all the key concerns noted in the initial reviews. The authors have added a new set of cryo-EM structures using a fully mature elongation complex scaffold with a 19-nt RNA to ensure their findings are robust. They also have revised interpretations to be consistent with the stochastic fluctuations of conformations expected to operate at molecular scale. The authors should be commended for such a positive response to peer review, which has substantially improved their manuscript. The manuscript is appropriate for publication with only minor revisions that can easily be accomplished in preparation of a final manuscript. I have only two scientific points to suggest for minor rewording to more accurately state the interpretation of the results and a few minor grammatical or presentation issues that could be improved.

Scientific points

1. The suggestion that NTP associates with the E-site prior to transition to the A-site (lines 160-161) is appropriate but the results don't distinguish between an ordered binding of NTP to the E-site then to the A-site versus random order initial binding to the A- or E-sites with NTPs shuttling between them prior to translocation and complementary NTP selection. I suggest revising this sentence to read "Together, these structures (States I–III) suggest that the substrate may initially bind non-specifically at the E-site." The authors may wish to consider whether to suggest to random-order binding model as an alternative interpretation.

We thank the reviewer for this helpful comment. The sentence has been revised accordingly.

2. In the discussion of whether state I occurs only when the RNA–DNA hybrid is not yet fully established or also occurs in mature elongation complexes (lines 438-439), I think readers would benefit from a more explicit statement of the uncertainty. I suggest just modifying the last sentence of the paragraph to read "Further studies will be needed to elucidate the dynamic properties of State I and its potential roles in initiating versus mature elongation complexes."

We thank the reviewer for this valuable suggestion. The text has been added to provide a clearer and more explicit statement.

Minor points. The manuscript is easy to follow as written but a few grammatical glitches in the intro could be fixed easily. Two issues in Fig 1 still need to be addressed.

1. Line 60 Should be “The NAC....”
We thank the reviewer for this suggestion. The phrase has now been revised.

2. Line 80, It would suffice to write instead “This product state is short-lived and challenging to capture....”
We thank the reviewer for this comment. The sentence has been revised to read as suggested.

3. Line 86 would read better as “How does *the* TL fold to a closed conformation upon substrate binding and then unfold after catalysis?”
We thank the reviewer for this suggestion. The sentence has been revised to read as suggested.

4. Line 88 would read better as “Does substrate binding lead to allosteric conformational changes in structural motifs beyond the TL, and how are these motions communicated?”
We thank the reviewer for raising this point. The sentence has been revised to read as suggested.

5. Line 96 would read better as “Substrate binding initiates long-range allosteric conformational changes....”
We thank the reviewer for this suggestion. The sentence has been revised to read as suggested.

6. In fig. 1 panel C, color i+1 tDNA base yellow as in panels D and E
We thank the reviewer for pointing out this inconsistency. The formatting has been corrected and standardized in yellow.

7. In fig. 1 panel E, move the label i+2 so that it is next to the i_2 base instead of next to the i+3 base where it is in the submitted figure.
We thank the reviewer for this comment. The layout and typesetting have been revised accordingly.

Reviewer #2 (Remarks to the Author)

The authors have addressed all my concerns sufficiently.
We thank the reviewer for the positive feedback.

Reviewer #3 (Remarks to the Author)

The authors have addressed all of my concerns.
We thank the reviewer for the supportive comment.
