# Supplementary material for: Structural dynamics of RNA polymerase II throughout the nucleotide addition cycle
Source: Nat Commun. 2026 Jul 8;17:8440. doi: 10.1038/s41467-026-75257-5 (PMC13478619; doi:10.1038/s41467-026-75257-5)
Supplement: Supplementary file 3 [file 41467_2026_75257_MOESM1_ESM.pdf]

Supplementary Information

Structural Dynamics of RNA Polymerase II Throughout the Nucleotide Addition Cycle

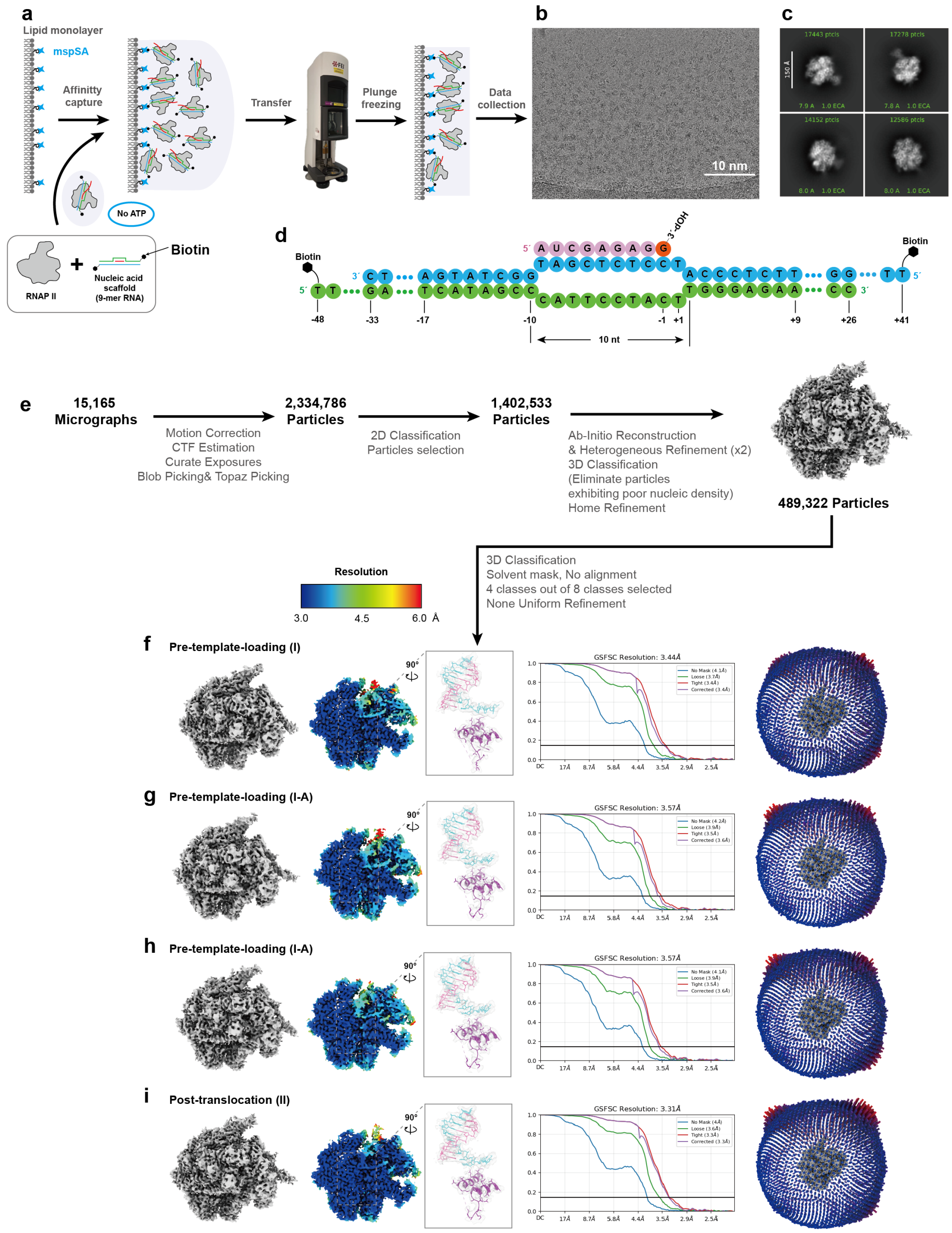

Supplementary Figure 1 | Data processing of cryo-EM structures of RNAPII EC without substrate (Sample 1).

(a) Schematic of mspSA affinity grid preparation and cryo-EM data collection for Sample 1. The nucleic acid scaffold was biotinylated to immobilize the RNAPII EC on the grid, thereby reducing particle orientation bias.

(b) Representative raw micrographs of the RNAPII EC. Similar images were captured on over three separate occasions.

(c) Gallery of reference-free 2D class averages of the RNAPII EC.
(d) Cartoon scheme for nucleic acid scaffold used in the Sample 1.
(e) Data processing workflow for Sample 1.
(f–i) RNAPII EC structures were determined in this Sample. Each structure comprises five representations arranged from left to right: the cryo-EM density map, a slice view colored by local resolution, a close-up of the active center, the Fourier shell correlation (FSC) curve, and the angular distribution plot. In each close-up view, it contains the TL (purple), RNA primer and ATP substrate (hot pink), and template DNA (cyan).

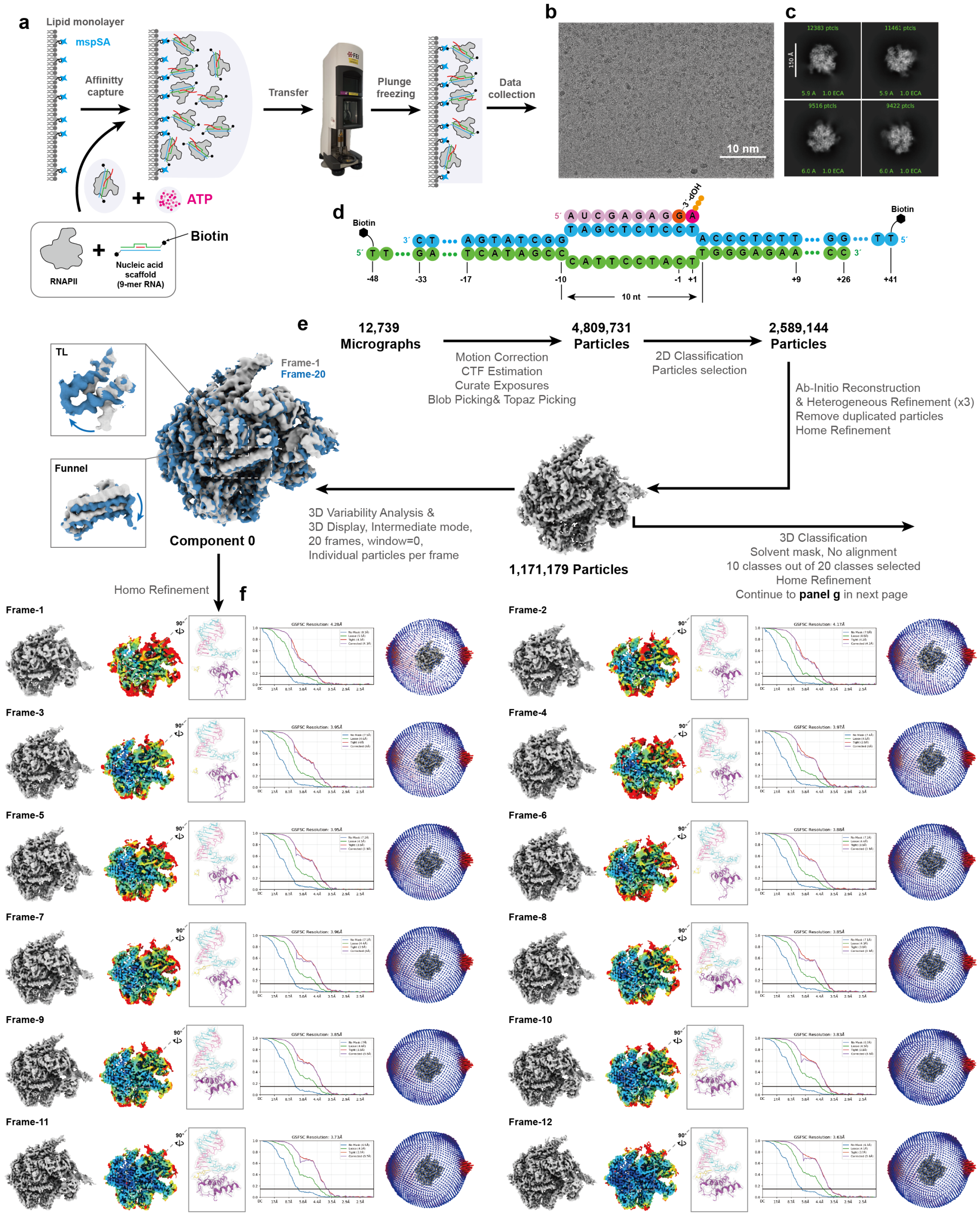

a
Lipid monolayer
mspSA
Affinitty capture
Transfer
Plunge freezing
Data collection
ATP
Biotin
RNAPII
Nucleic acid scaffold (9-mer RNA)
b
10 nm
c
d
Biotin
5´ A U C G A G A G G A
3´-OH
-48
-33
-17
-10
-1
+1
+9
+26
+41
10 nt
e
12,739 Micrographs
Motion Correction
CTF Estimation
Curate Exposures
Blob Picking& Topaz Picking
4,809,731 Particles
2D Classification
Particles selection
2,589,144 Particles
Ab-Initio Reconstruction
& Heterogeneous Refinement (x3)
Remove duplicated particles
Home Refinement
1,171,179 Particles
3D Variability Analysis &
3D Display, Intermediate mode,
20 frames, window=0,
Individual particles per frame
TL
Funnel
Frame-1
Frame-20
Component 0
3D Classification
Solvent mask, No alignment
10 classes out of 20 classes selected
Home Refinement
Continue to panel g in next page
Homo Refinement
f
Frame-1
Frame-2
Frame-3
Frame-4
Frame-5
Frame-6
Frame-7
Frame-8
Frame-9
Frame-10
Frame-11
Frame-12

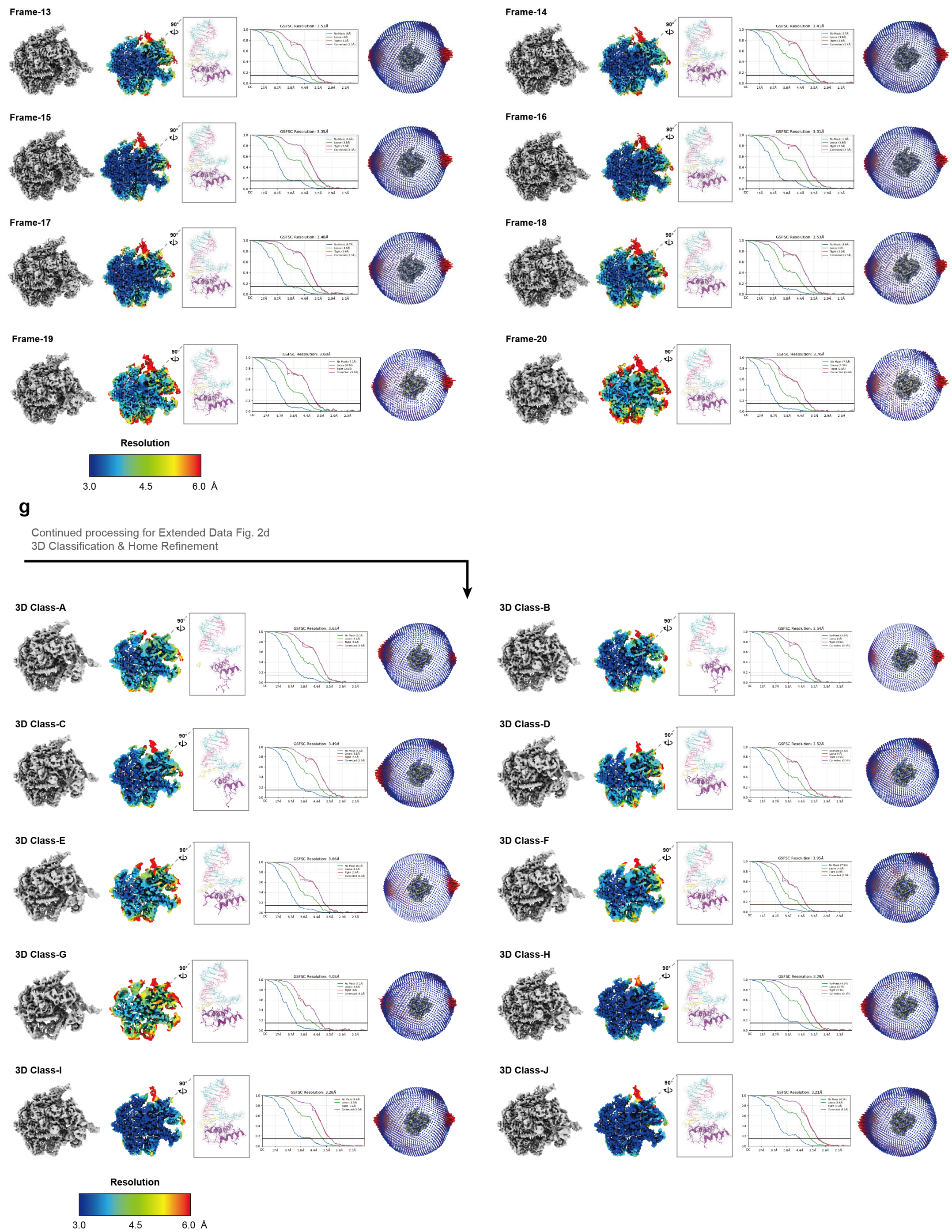

Supplementary Figure 2 | Data processing of cryo-EM structures of RNAPII EC with substrate ATP (Sample 2)

(a) Schematic of mspSA affinity grid preparation and cryo-EM data collection for Sample 2. Substrate ATP was included to capture conformational dynamics in the pre-catalytic phase.

(b) Representative raw micrographs of the RNAPII EC with ATP. Similar images were

captured on over three separate occasions.
(c) Gallery of reference-free 2D class averages of the RNAPII EC containing substrate ATP.
(d) Cartoon scheme for nucleic acid scaffold used in the cryo-EM Sample of RNAPII EC with substrate ATP.
(e) Data processing workflow for the RNAPII EC substrate binding Sample.
(f and g) RNAPII EC structures were determined from 3D Variability Analysis (f, from Frame 1–20) and 3D classification with a solvent mask (g, from class A–J). Each structure comprises five representations arranged from left to right: the cryo-EM density map, a slice view colored by local resolution, a close-up of the active center, the Fourier shell correlation (FSC) curve, and the angular distribution plot. In each close-up view, it contains the TL (purple), RNA primer and ATP substrate (hot pink), and template DNA (cyan).

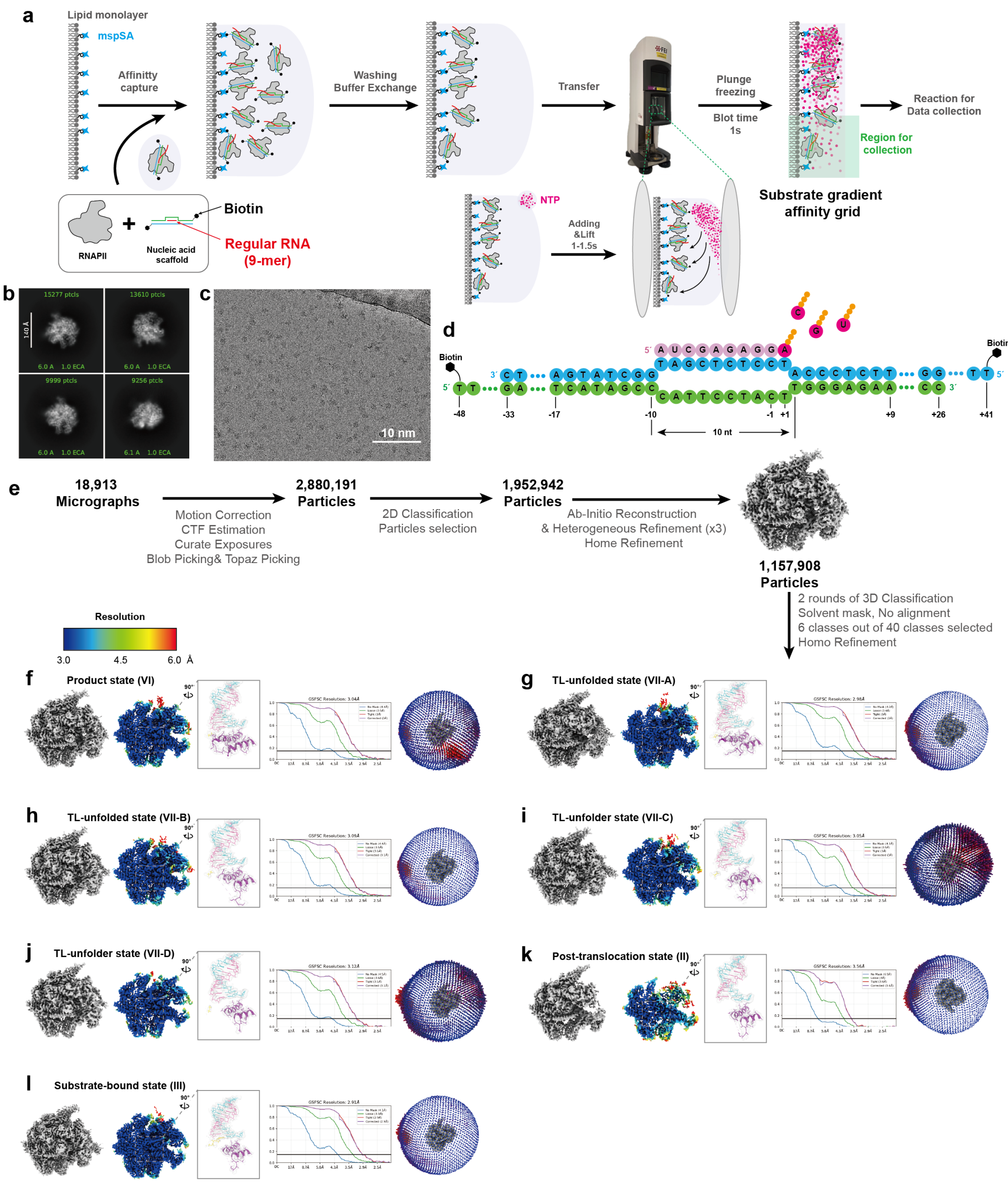

Supplementary Figure 3 | Data processing of cryo-EM structures of RNAPII EC with substrate NTP (Sample 3)

(a) Schematic of substrate-gradient grid (SGG) preparation and cryo-EM data collection for Sample 3. A regular 9-mer RNA scaffold and substrate NTPs (ATP, UTP, CTP, and GTP) were used to enable RNA extension and capture conformational states in the post-catalytic phase. (See Methods for details of substrate-gradient grid preparation.)

(b) Representative raw micrographs. Similar images were captured on over three separate

occasions.
(c) Gallery of reference-free 2D class averages.
(d) Cartoon scheme for nucleic acid scaffold used in Sample 3.
(e) Data processing workflow.
(f–l) RNAPII EC structures were determined in Sample 3. Each structure comprises five representations arranged from left to right: the cryo-EM density map, a slice view colored by local resolution, a close-up of the active center, the Fourier shell correlation (FSC) curve, and the angular distribution plot. In each close-up view, it contains the TL (purple), RNA primer and ATP substrate (hot pink), and template DNA (cyan).

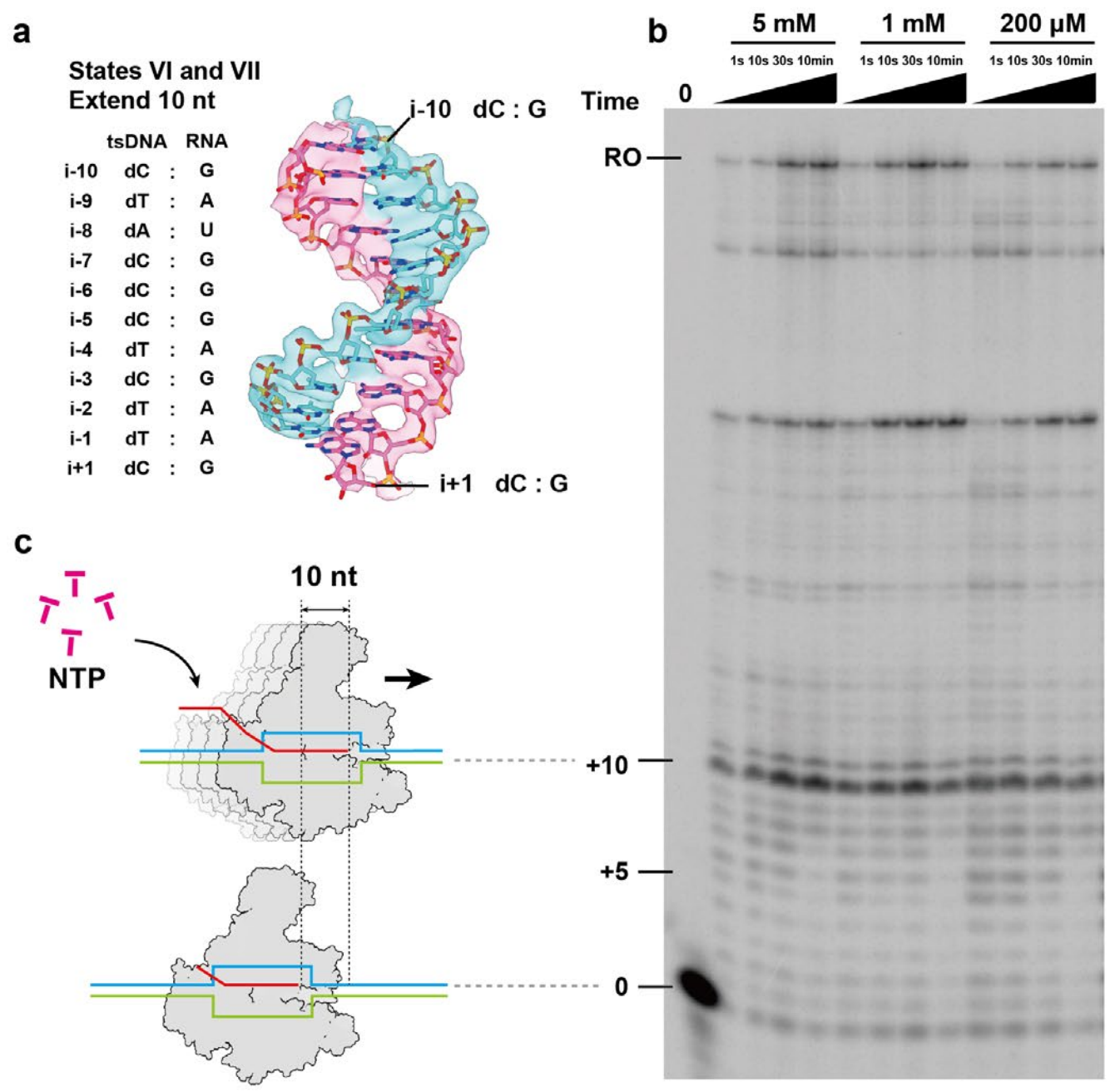

Supplementary Figure 4 | Transcription assays of RNAPII EC incorporating NTP

(a) RNAPII EC extends RNA by 10 nucleotides (nt) upon NTP addition, as observed in the post-catalysis cryo-EM Sample. The corresponding RNA-DNA hybrid sequence for states VI and VII is shown on the left. The cryo-EM structure of the RNA-DNA hybrid in RNAPII EC (State VI) is presented on the right, visualized as densities and stick models. Template DNA and RNA are colored cyan and hot pink, respectively.

(b) Transcription assays of RNAPII EC conducted with substrate NTP at three different concentrations: 5 mM, 1 mM, and 200 µM. For each concentration, reactions were quenched at various time points: 5 s, 10 s, 30 s, and 10 min. Markers indicating nucleotide addition are annotated on the side of the gel, and the run-off product is labeled as RO. Consistent results were obtained across three independent trials.

(c) Cartoon schematics depicting the elongation of RNAPII EC, illustrating that the elongation complex predominantly pauses after extending by 10 nucleotides.

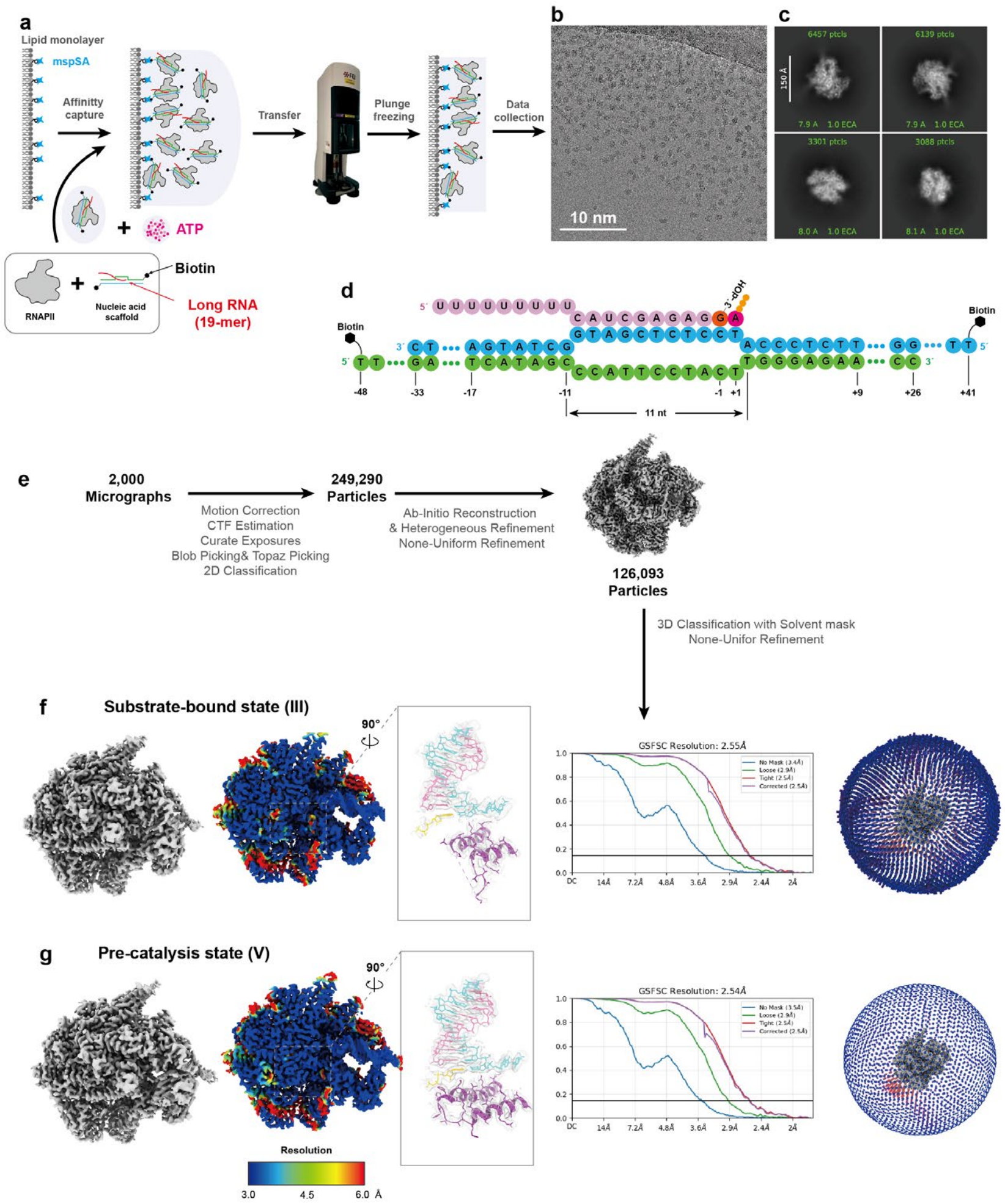

Supplementary Figure 5 | Data processing of cryo-EM structures of long-RNA EC with substrate ATP (Sample 4)

(a) Schematic of mspSA affinity grid preparation and cryo-EM data collection for Sample 4. A long RNA (19-mer) was used to assemble the EC, mimicking a mature EC and enabling cross-validation with Sample 2.

(b) Representative raw micrographs. Similar images were captured on over three separate occasions.

(c) Gallery of reference-free 2D class averages.

(d) Cartoon scheme for nucleic acid scaffold used in the Sample 4.

(e) Data processing workflow.

(f and g) Two RNAPII EC structures were determined in Sample 4. Each structure comprises five representations arranged from left to right: the cryo-EM density map, a slice view colored by local resolution, a close-up of the active center, the Fourier shell

correlation (FSC) curve, and the angular distribution plot. In each close-up view, it contains the TL (purple), RNA primer and ATP substrate (hot pink), and template DNA (cyan).

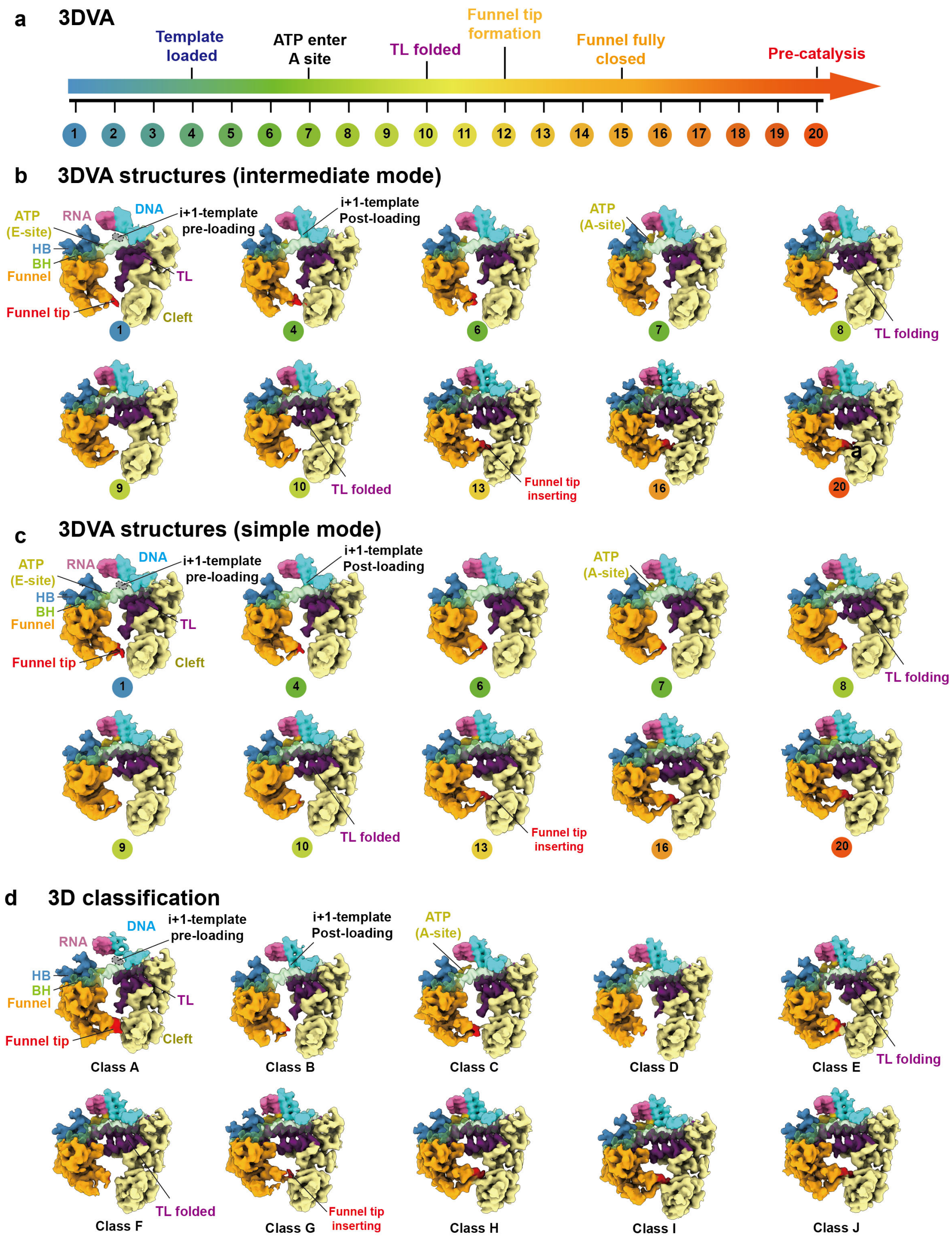

Supplementary Figure 6 | Validation of RNAPII EC conformational dynamic cascade in the presence of ATP

(a) Spectrum illustrating the conformational transitions captured by 3D variability analysis (3DVA) structures from Frame 1 (blue) to Frame 20 (red). Key conformational events associated with specific frames are annotated.

(b–c) Cryo-EM structures of the active center of RNAPII EC, captured at various 3DVA frames derived from two distinct data processing approaches: intermediate mode (b) and

simple mode (c). Structures are displayed as cryo-EM densities and colored according to RNAPII EC components: RNA (hot pink), template DNA (cyan), bridge helix (BH, light green), hybrid binding domain (HB, steel blue), TL (purple), funnel (orange), ATP (gold), and funnel tip (red). Frame numbers corresponding to each structure are indicated below. Key conformational events are annotated alongside the structures for clarity.
(d) Cryo-EM structures generated through 3D classification. These structures illustrate conformational events similar to those captured by 3DVA structures. The 3D classes A-J correspond to respective frame numbers in the 3DVA (b, c).

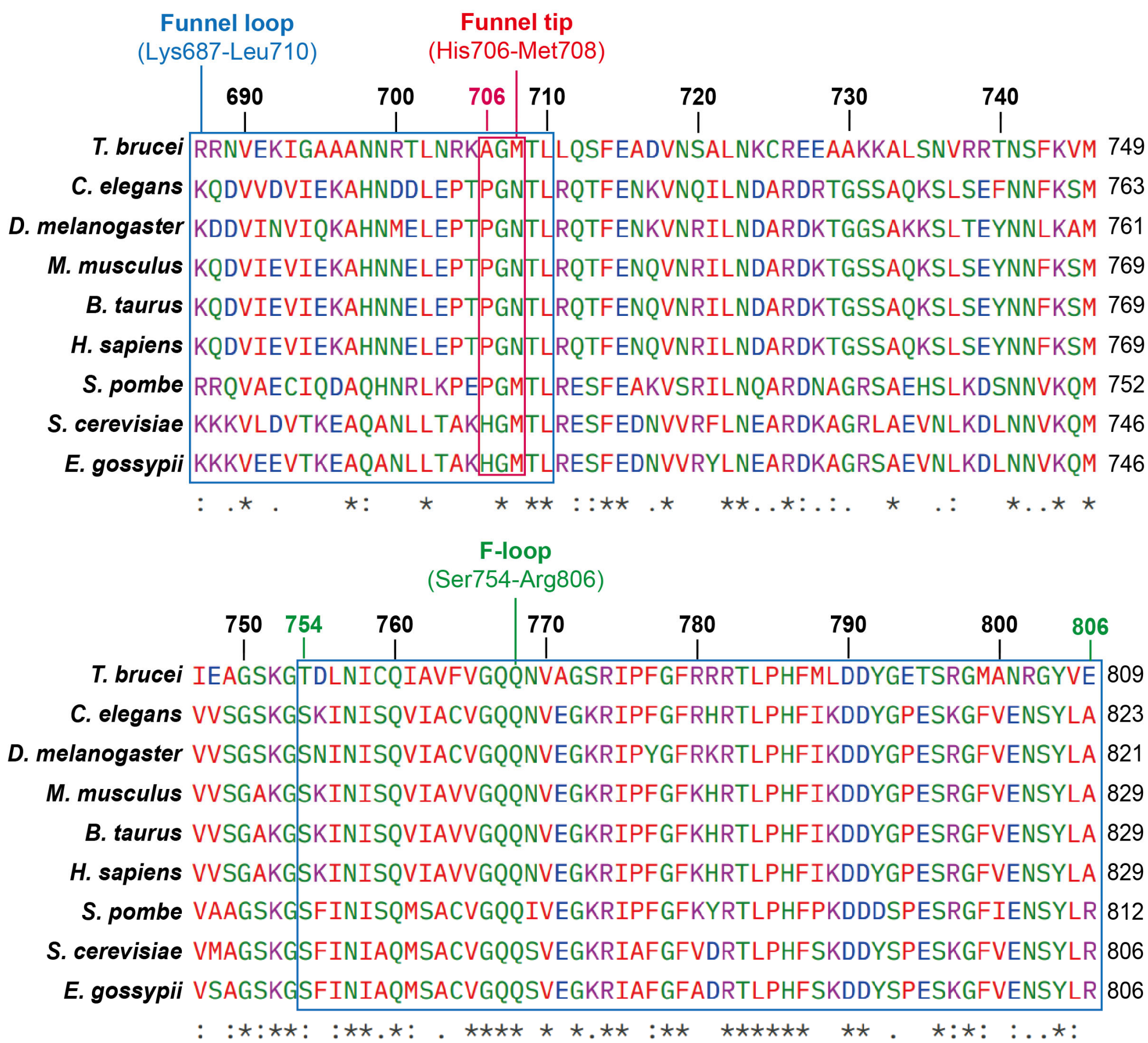

Supplementary Figure 7 | Multi-sequence alignment of funnel tip

Protein sequences of Rpb1 are aligned by the program of Clustal Omega (1.2.4)[1]. There are nine Rpb1 sequences compared here, including *Saccharomyces cerevisiae*, *Schizosaccharomyces pombe*, *Eremothecium gossypii*, *Trypanosoma brucei*, *Caenorhabditis elegans*, *Drosophila melanogaster*, *Bos taurus*, *Mus musculus*, and *Homo sapiens*. The color codes for amino acid letters are based on their polarity and electrostatics. The funnel tip and loop regions are highlighted with red and blue circlens, respectively.

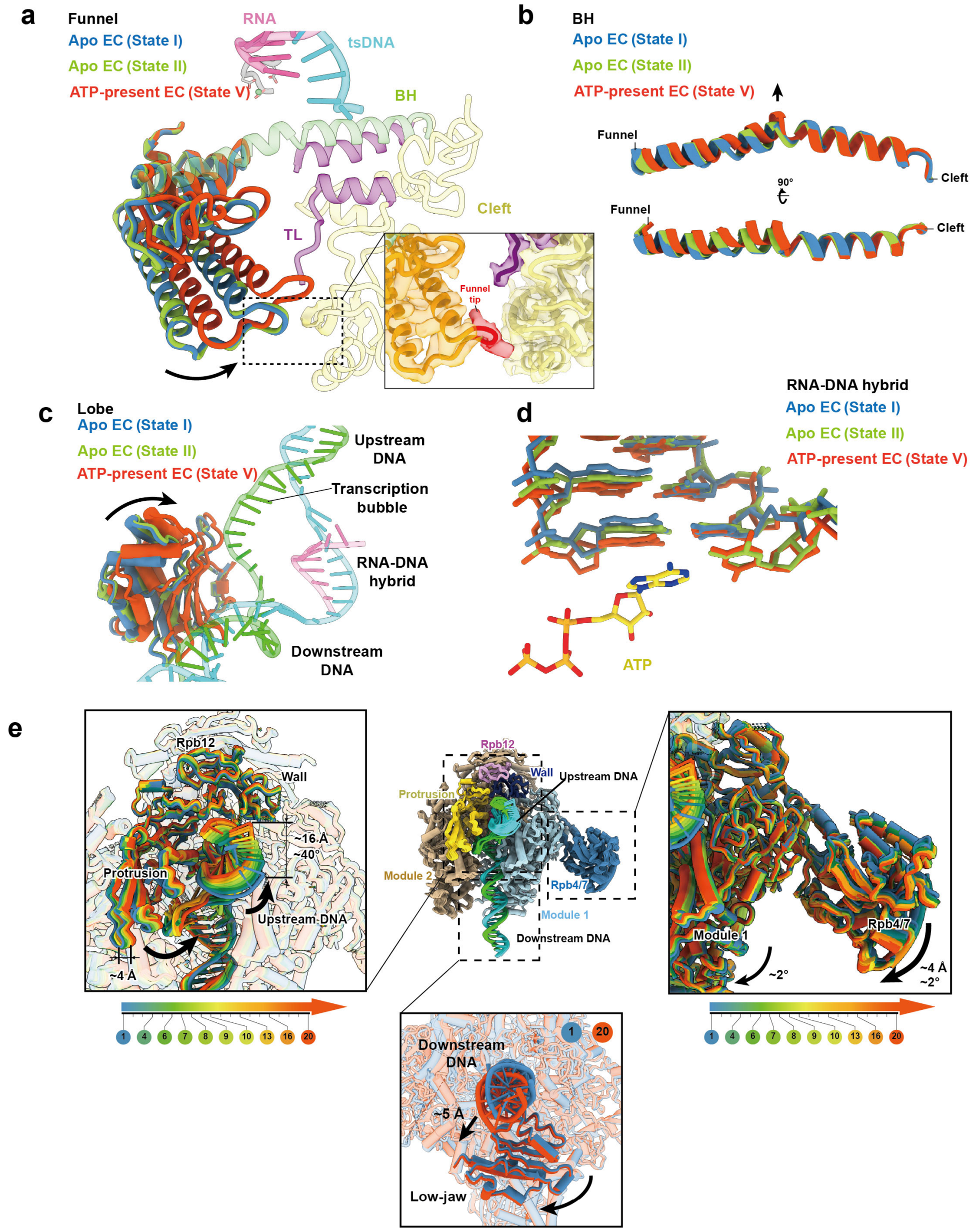

Supplementary Figure 8 | Structural insights of substrate-induced EC tightening

(a–d) Superposition of funnel domain (a), bridge helix (b), lobe (c), and RNA-DNA hybrid (d) across three structures: the pre-template-loading state (state I, blue) and the post-translocation state (state II, light green) from the apo EC Sample (Sample 1), and the pre-catalysis state structure (state V, red, Frame 20) from the substrate ATP-bound EC Sample (Sample 2). The zoom-in panel in panel (a) shows cryo-EM densities of the funnel domain

with an emphasis on the funnel tip region highlighted in red. Other RNAPII domains are depicted as transparent ribbons, with domain name annotated in matching colors for clarity. (e) Conformational dynamics of Rpb4/7 stalk, upstream DNA and downstream DNA. The middle panel shows the top view of RNAPII EC, demonstrating conformational dynamics across selected frames from the Sample 2. Multiple structural frames (frames 1, 4, 6, 7, 8, 9, 10, 13, 16, and 20) are superimposed to illustrate the overall range of motion. Structural elements are color-coded as follows: module 1 (light blue), module 2 (tan), protrusion domain (gold), wall domain (navy), and Rpb12 (plum). Three close-up views of the Rpb4/7 stalk (right) downstream DNA, and upstream DNA (left) correspond to the local region indicated in middle panel. Each close-up view shows the superposition of selected frames as indicated, which are colored according to the dynamics spectrum from blue (open) to red (closed). They illustrate the gradual and continuous movement of the Rpb4/7 stalk or upstream DNA during the conformational transition. The direction of motion is indicated by black arrows, and the corresponding displacement and rotation are annotated accordingly.

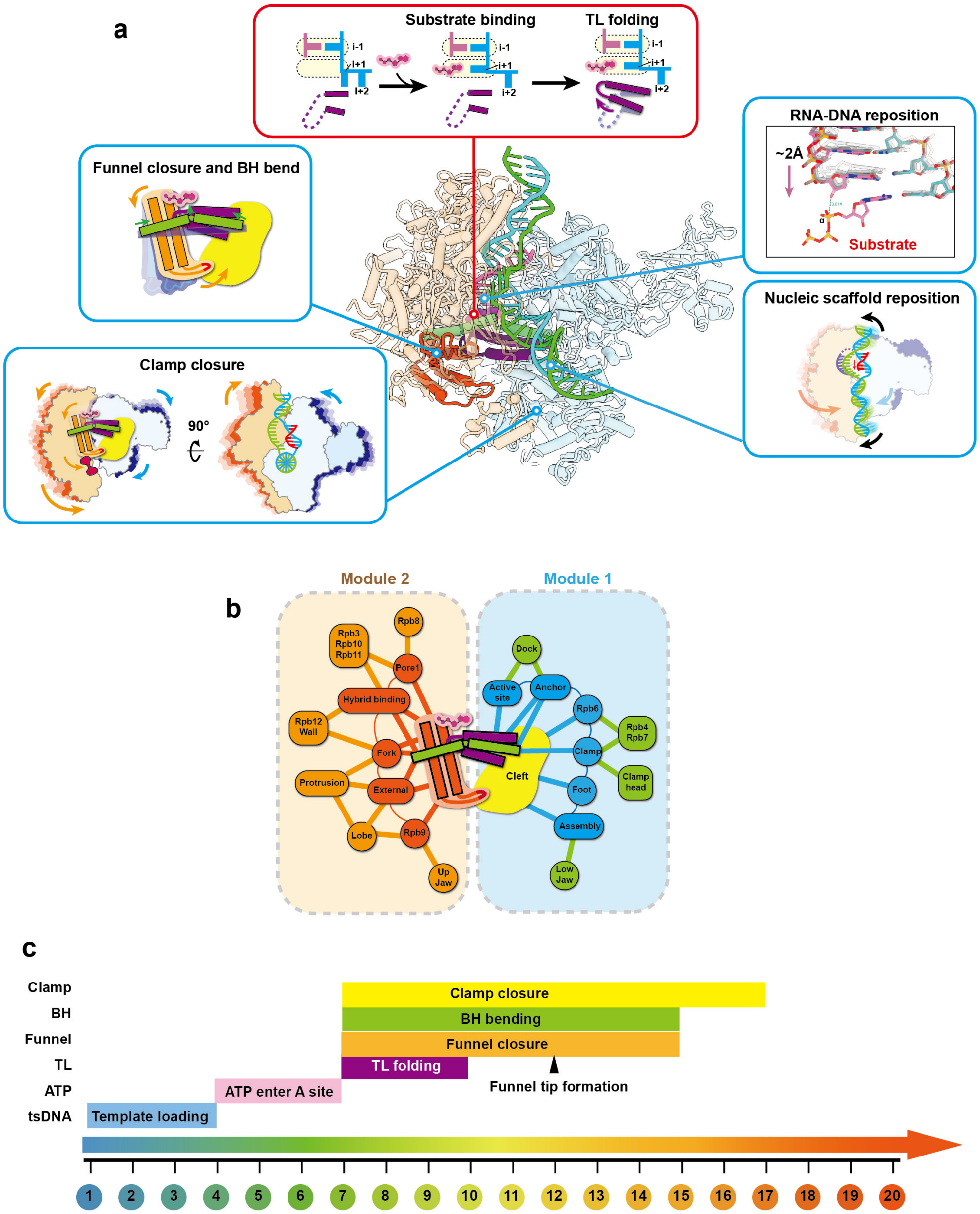

Supplementary Figure 9 | Additional analysis of substrate-induced EC tightening

(a) Schematic representation of coordinated conformational changes in RNAPII during catalysis. A central RNAPII structure is shown with key structural elements highlighted, including the substrate (magenta), TL (purple), BH (light green), funnel (orange red), module 1 (light blue) and module 2 (tan). Surrounding cartoon diagrams illustrate the conformational changes of individual elements. Each schematic is connected to its

corresponding structural location by lines, emphasizing spatial mapping rather than causal relationships. Previously reported conformational changes are indicated by red boxes, whereas newly identified changes in this study are highlighted in blue boxes.
(b) Interaction network underlying RNAPII conformational dynamics. The network is centered on the catalytic region, including the substrate, TL, funnel, BH and Cleft. RNAPII is divided into two modules, module 1 and 2. First-layer interactions are highlighted within module 2 (dark orange) and module 1 (sky blue), representing elements directly connected to the core region. Peripheral interactions are further indicated in module 2 (light orange) and module 1 (light green), illustrating an extended interaction network.
(c) Conformational dynamics spectrum derived from the Sample 2. A series of 20 structural frames (states I–V and intermediates) are arranged to represent the continuous transition from an open to a tightened RNAPII conformation. Key conformational dynamic events are mapped onto this spectrum. The intervals highlighted for each structural element correspond to the frames in which the most pronounced conformational changes occur. These assignments are based on comparative analyses of local structural deviations between frames. Importantly, the indicated intervals represent regions of dominant structural rearrangement rather than the full extent of conformational variation. Structural elements may continue to undergo more subtle adjustments outside these intervals, including side-chain rearrangements or local density differences, which are not explicitly highlighted in this representation.

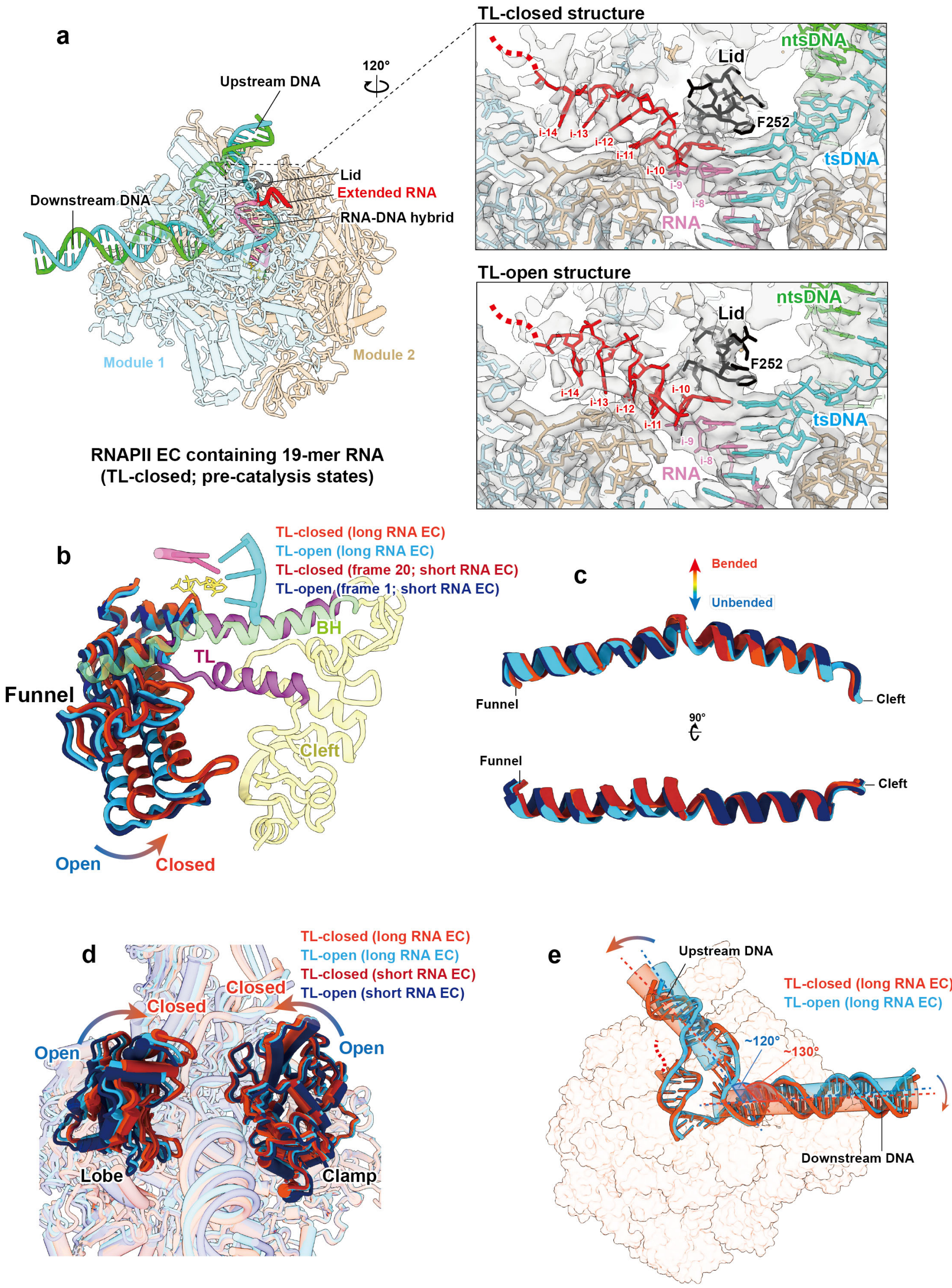

Supplementary Figure 10 | Cryo-EM structures of RNAPII EC containing long RNA (19-mer)

(a) Overall structure of the RNAPII elongation complex (EC) assembled with an extended 19-mer RNA in the TL-closed state, representing a more complete elongation state compared to short-RNA complexes. The left panel shows the global architecture of the EC, with module 1 colored light blue and module 2 in tan. The nucleic acid scaffold includes tsDNA (cyan), ntsDNA (lime), and RNA (hot pink), with the extended upstream RNA

highlighted in red. The right panel shows zoom-in views of the upstream RNA junction in two conformational states: TL-closed (upper) and TL-open (lower). Cryo-EM densities and corresponding atomic models illustrate RNA extension and strand separation from the tsDNA. The lid domain is colored black, with residue F252 highlighted.

(b–d) Structural comparisons of key dynamic elements across four RNAPII EC structures. The funnel domain (b), bridge helix (c), and lobe/clamp domain (d) are compared between long-RNA Sample 4 (TL-closed, orange red; TL-open, sky blue) and short-RNA Sample 2 (frame 20, firebrick; frame 1, navy). A continuous color gradient from blue (open) to red (closed) illustrates domain movements.

(e) Nucleic acid scaffold rearrangements accompanying EC tightening in the long-RNA Sample. Comparison of TL-open (sky blue) and TL-closed (orange red) states reveals coordinated clamp closure and global nucleic acid repositioning. Arrows indicate directional movements of upstream and downstream DNA. The axes of upstream and downstream DNA are represented by shaded cylinders (blue and red, respectively), highlighting changes in DNA trajectory.

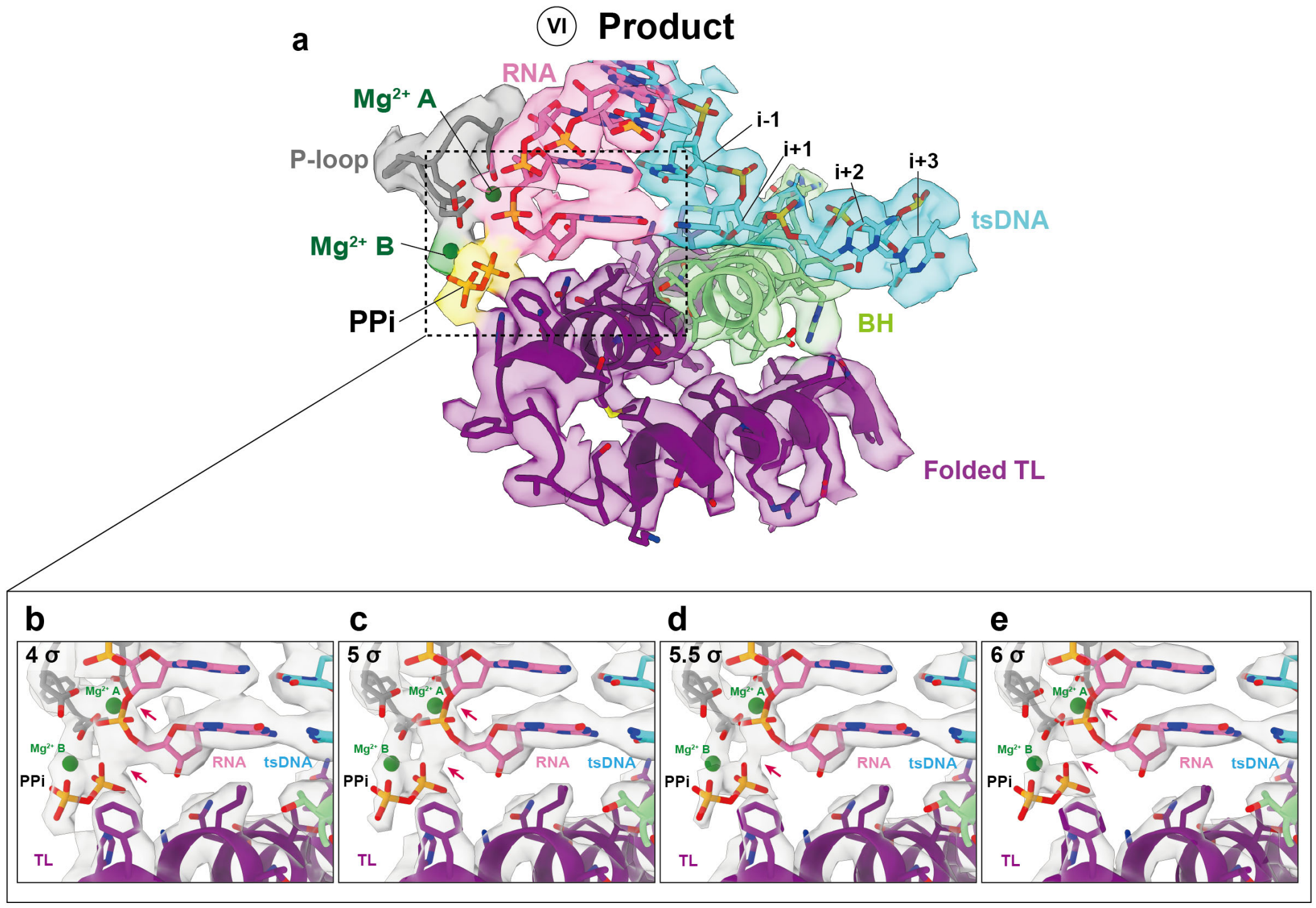

Supplementary Figure 11 | Validation of the product state by cryo-EM density maps. (a) Cryo-EM structure of the RNA polymerase II elongation complex (EC) captured in the product state (VI). The density map is shown at a contour level of 0.132 (3-σ). Color coding of RNAPII elements: trigger loop (TL, purple), bridge helix (BH, light green), template DNA (tsDNA, cyan), and RNA primer (hot pink).

(b-e) Cryo-EM densities highlighting the newly formed phosphodiester bond and the released pyrophosphate (PPi), displayed at different contour levels: 0.176 (4-σ, panel b), 0.220 (5-σ, panel (c)), 0.242 (5.5-σ, panel (d)), and 0.264 (6-σ, panel (e)). Red arrows indicate the positions of bond formation and cleavage.

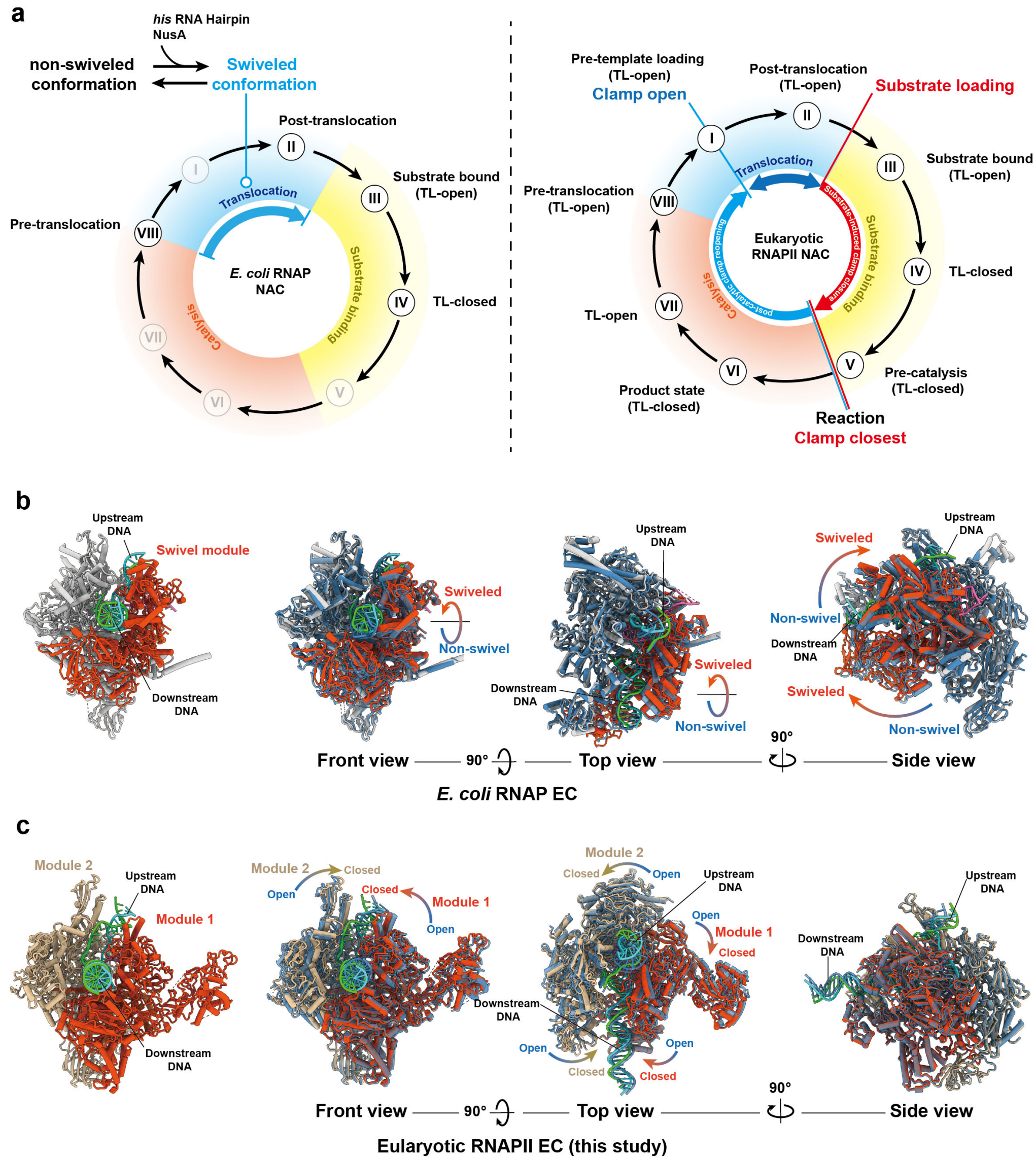

Supplementary Figure 12 | Comparison between bacterial RNAP swiveling and eukaryotic RNAPII tightening

(a) Schematic representation of the nucleotide addition cycle (NAC) in bacterial RNA polymerase (left) and eukaryotic RNA polymerase II (right), with conformational transitions mapped onto distinct stages of the cycle. The NAC is divided into three major phases: translocation (blue), substrate binding (yellow), and catalysis (orange). Roman numerals indicate structurally defined intermediate states. Eight states are resolved for RNAPII, whereas only a subset of states has been structurally characterized in bacterial RNAP; unresolved states are depicted in semi-transparent shading. In bacterial RNAP (left), swiveling is coordinated with the translocation phase of the NAC. This conformational equilibrium can be modulated by transcription factors (e.g., NusA[2]) or pausing signals

such as his RNA hairpins[3], which bias the enzyme between non-swiveled and swiveled states. In contrast, eukaryotic RNAPII (right) exhibits a distinct mode. Substrate binding promotes clamp closure (red arrow), progressing toward a maximally closed state near the catalytic step. Following catalysis, the clamp reopens (blue arrow), returning toward a more open conformation. The timing of substrate loading, as well as the most open and most closed clamp conformations, are indicated along the cycle.

(b–c) Structural comparison of bacterial RNAP swiveling and eukaryotic RNAPII clamp dynamics.

(b) Bacterial RNAP swiveling. The leftmost panel shows the cryo-EM structure of *E. coli* RNAP in the swiveled state (PDB: 6ASX). The swiveling module (orange-red) partially overlaps with module 1 in eukaryotic RNAPII. Three orthogonal views (front, top, and side) depict the reversible transition between the non-swiveled (PDB: 6BJS) and swiveled states, with arrows indicating the direction of motion. (c) Eukaryotic RNAPII clamp closure. By contrast, the leftmost panel shows the cryo-EM structure of the RNAPII elongation complex (EC) in the pre-catalytic state (state V) determined in this study. Two major structural modules are highlighted: module 1 (orange red) and module 2 (light blue). Three orthogonal views (front, top, and side) illustrate the coordinated, progressive movement of these modules during clamp dynamics, with gradient arrows indicating the transition from the open (state I; pre-template loading) to the closed (state V; pre-catalysis) conformation.

Supplementary Table 1 | Cryo-EM data collection and structure determination of RNAPII EC without substrate (Sample 1)

Map	State-I	State-I-A	State-I-B	State-II
**PDB**	9SAY	NA	NA	9SAZ
**EMDB**	EMD-54704	EMD-54705	EMD-54706	EMD-54707
**Data collection**				
Microscope	Titan Krios Gatan			
Detector	K3 with GIF Quantum			
Cs (mm)	2.7	2.7	2.7	2.7
Magnification	105k	105k	105k	105k
Pixel size (Å)	0.829	0.829	0.829	0.829
Electron dose (e-/Å2)	50	50	50	50
Defocus range (µm)	-0.5 to -2.5	-0.5 to -2.5	-0.5 to -2.5	-0.5 to -2.5
Micrograph Number	15165	15165	15165	15165
**Reconstruction**				
Software	cryoSPARC-4.5			
Particles refinement	64243	57585	56940	72426
Symmetry	C1	C1	C1	C1
Resolution (Å)	3.4	3.6	3.6	3.3
Sharpening B-factor (Å2)	85.3	79.2	78.8	82.7
**Refinement**				
**Model composition**				
Number of atoms	29640	NA	NA	32314
Protein residues	3557	NA	NA	3896
Nucleotides	65	NA	NA	65
Ligand	1 MG; 8 ZN	NA	NA	1 MG; 8 ZN
**Bonds RMSD**				
Bonds lengths (Å)	0.0027	NA	NA	0.0023
Bonds angles (°)	0.52	NA	NA	0.49
**Validation**				
MolProbity score	1.70	NA	NA	1.58
Clash score	8.16	NA	NA	7.01
Rotamer outliers	0.00%	NA	NA	0.15%
C-beta outliers	0.00%	NA	NA	0.00%
**Ramachandran plot**				
Favored (%)	96.20%	NA	NA	96.82%
Allowed (%)	3.69%	NA	NA	3.13%
Outlier (%)	0.11%	NA	NA	0.05%

Supplementary Table 2 | Cryo-EM data collection and structure determination of RNAPII EC with ATP substrate, and 3D Variability Analyses (Sample 2)

Map	Frame 1	Frame 2	Frame 3	Frame 4	Frame 5	Frame 6	Frame 7	Frame 8	Frame 9	Frame 10	Frame 11	Frame 12	Frame 13	Frame 14	Frame 15	Frame 16	Frame 17	Frame 18	Frame 19	Frame 20
**PDB**	9SBL	9SBM	9SBN	9SBO	9SBP	9SBQ	9SBR	9SBS	9SBT	9SBU	9SBV	9SBW	9SBX	9SBY	9SBZ	9SC0	9SC1	9SC2	9SC3	9SC4
**EMDB**	EMD-54730	EMD-54731	EMD-54732	EMD-54733	EMD-54734	EMD-54735	EMD-54736	EMD-54737	EMD-54738	EMD-54739	EMD-54740	EMD-54741	EMD-54742	EMD-54743	EMD-54744	EMD-54745	EMD-54746	EMD-54747	EMD-54748	EMD-54749
**Data collection**																				
Microscope										Titan Krios Gatan										
Detector										K3 with GIF Quantum										
Cs (mm)										2.7										
Magnification										105k										
Pixel size (Å)										0.825										
Electron dose (e-/Å2)										50										
Defocus range (μm)										-0.5 to -2.5										
Micrograph Number										12,739										
**Reconstruction**																				
Software										cryoSPARC-4.5										
Particles refinement	16,262	22,773	29,765	37,985	46,826	56,496	66,397	77,148	86,647	94,160	98,766	100,314	97,126	89,544	79,845	67,276	53,389	40,178	29,059	19,500
Symmetry	C1	C1	C1	C1	C1	C1	C1	C1	C1	C1	C1	C1	C1	C1	C1	C1	C1	C1	C1	C1
Resolution (Å)	4.3	4.2	4.0	4.0	4.0	3.9	4.0	3.9	3.9	3.8	3.7	3.6	3.5	3.4	3.4	3.3	3.5	3.5	3.7	3.8
Sharpening B-factor (Å2)	47.2	56.6	57.3	64.5	64.2	70.00	69.9	68.5	79.6	93.0	71.9	78.70	78.9	75.0	66.7	64.5	62.9	64.10	57.7	43.6
**Refinement**																				
**Model composition**																				
Number of atoms	32888	32901	32901	32901	32901	32895	32914	32965	32987	33083	33083	33083	33083	33083	33083	33083	33083	33083	33083	33083
Protein residues	3895	3897	3897	3897	3897	3896	3896	3902	3905	3908	3908	3908	3908	3908	3908	3908	3908	3908	3908	3908
Nucleotides	93	93	93	93	93	93	93	93	93	97	97	97	97	97	97	97	97	97	97	97
Ligand	8 ZN; 1 MG; 1 ATP	8 ZN; 1 MG; 1 ATP	8 ZN; 1 MG; 1 ATP	8 ZN; 1 MG; 1 ATP	8 ZN; 1 MG; 1 ATP	8 ZN; 1 MG; 1 ATP	8 ZN; 2 MG; 1 ATP	8 ZN; 2 MG; 1 ATP	8 ZN; 2 MG; 1 ATP	8 ZN; 2 MG; 1 ATP	8 ZN; 2 MG; 1 ATP	8 ZN; 2 MG; 1 ATP	8 ZN; 2 MG; 1 ATP	8 ZN; 2 MG; 1 ATP	8 ZN; 2 MG; 1 ATP	8 ZN; 2 MG; 1 ATP	8 ZN; 2 MG; 1 ATP	8 ZN; 2 MG; 1 ATP	8 ZN; 2 MG; 1 ATP	8 ZN; 2 MG; 1 ATP
**Bonds RMSD**																				
Bonds lengths (Å)	0.003	0.004	0.003	0.003	0.004	0.004	0.023	0.023	0.023	0.023	0.023	0.023	0.023	0.024	0.023	0.023	0.023	0.023	0.023	0.023
Bonds angles (°)	0.60	0.61	0.56	0.57	0.60	0.59	0.95	0.93	0.94	0.95	0.92	0.92	0.94	0.97	0.92	0.89	0.93	0.92	0.93	0.93
**Validation**																				
MolProbity score	1.90	1.95	1.80	1.71	1.86	1.89	1.84	1.77	1.81	1.89	1.68	1.63	1.75	1.70	1.63	1.63	1.65	1.62	1.71	1.74
Clash score	12.98	12.79	10.31	9.37	10.08	10.36	10.04	8.97	9.84	10.47	8.38	7.30	8.59	7.67	6.73	6.83	7.00	7.64	7.95	8.93
Rotamer outliers	0.03%	0.03%	0.00%	0.00%	0.03%	0.00%	0.00%	0.00%	0.00%	0.20%	0.00%	0.00%	0.00%	0.00%	0.00%	0.00%	0.00%	0.00%	0.00%	0.00%
C-beta outliers	0.00%	0.00%	0.00%	0.00%	0.00%	0.00%	0.00%	0.00%	0.00%	0.00%	0.00%	0.00%	0.00%	0.00%	0.00%	0.00%	0.00%	0.00%	0.00%	0.00%
**Ramachandran plot**																				
Favored (%)	95.98%	95.20%	96.07%	96.61%	95.23%	94.92%	95.41%	95.78%	95.81%	94.96%	96.52%	96.47%	95.85%	95.90%	96.24%	96.24%	96.08%	96.73%	95.93%	96.13%
Allowed (%)	3.97%	4.64%	3.88%	3.34%	4.72%	5.00%	4.49%	4.09%	4.14%	4.88%	3.38%	3.43%	3.99%	4.00%	3.66%	3.66%	3.79%	3.14%	3.55%	3.55%
Outlier (%)	0.05%	0.16%	0.05%	0.05%	0.05%	0.08%	0.10%	0.13%	0.05%	0.16%	0.10%	0.10%	0.16%	0.10%	0.10%	0.10%	0.13%	0.13%	0.10%	0.13%

Supplementary Table 3 | Cryo-EM data collection and structure determination of RNAPII EC with ATP substrate, and 3D Classification Analyses (Sample 2)

Map	3D-Class-A	3D-Class-B	3D-Class-C	3D-Class-D	3D-Class-E	3D-Class-F	3D-Class-G	3D-Class-H	3D-Class-I	3D-Class-J
**EMDB**	EMD-54677	EMD-54682	EMD-54681	EMD-54680	EMD-54679	EMD-54683	EMD-54684	EMD-54685	EMD-54686	EMD-54687
**Data collection**										
Microscope	Titan Krios Gatan									
Detector	K3 with GIF Quantum									
Cs (mm)	2.7	2.7	2.7	2.7	2.7	2.7	2.7	2.7	2.7	2.7
Magnification	105k	105k	105k	105k	105k	105k	105k	105k	105k	105k
Pixel size (Å)	0.825	0.825	0.825	0.825	0.825	0.825	0.825	0.825	0.825	0.825
Electron dose (e-/Å2)	50	50	50	50	50	50	50	50	50	50
Defocus range (μm)	-0.5 to -2.5	-0.5 to -2.5	-0.5 to -2.5	-0.5 to -2.5	-0.5 to -2.5	-0.5 to -2.5	-0.5 to -2.5	-0.5 to -2.5	-0.5 to -2.5	-0.5 to -2.5
Micrograph Number	12739	12739	12739	12739	12739	12739	12739	12739	12739	12739
**Reconstruction**										
Software	cryoSPARC-4.5	cryoSPARC-4.5	cryoSPARC-4.5	cryoSPARC-4.5	cryoSPARC-4.5	cryoSPARC-4.5	cryoSPARC-4.5	cryoSPARC-4.5	cryoSPARC-4.5	cryoSPARC-4.5
Particles refinement	65010	73900	66560	93475	86363	44115	36095	69690	65841	88302
Symmetry	C1	C1	C1	C1	C1	C1	C1	C1	C1	C1
Resolution (Å)	3.6	3.6	3.5	3.5	3.7	4.0	4.1	3.3	3.3	3.2
Sharpening B-factor (Å2)	72.9	67.7	73.3	80.3	77.6	62.9	57.4	68.4	64.5	68.2

Supplementary Table 4 | Cryo-EM data collection and structure determination of RNAPII EC with NTP substrate (Sample 3)

**Map**	State-II	State-III	State-VI	State-VII-A	State-VII-B	State-VII-C	State-VII-D
**PDB**	9SB5	29RF	9SB0	9SB1	9SB2	9SB3	9SB4
**EMDB**	EMD-54713	EMD-57322	EMD-54708	EMD-54709	EMD-54710	EMD-54711	EMD-54712
**Data collection**							
Microscope				Titan Krios Gatan			
Detector				K3 with GIF Quantum			
Cs (mm)	2.7	2.7	2.7	2.7	2.7	2.7	2.7
Magnification	105k	105k	105k	105k	105k	105k	105k
Pixel size (Å)	0.932	0.932	0.932	0.932	0.932	0.932	0.932
Electron dose (e-/Å^2)	50	50	50	50	50	50	50
Defocus range (μm)	-0.5 to -2.5	-0.5 to -2.5	-0.5 to -2.5	-0.5 to -2.5	-0.5 to -2.5	-0.5 to -2.5	-0.5 to -2.5
Micrograph Number	18913	18913	18913	18913	18913	18913	18913
**Reconstruction**							
Software				cryoSPARC-4.5			
Particles refinement	28966	39522	28752	30935	29809	28225	25539
Symmetry	C1	C1	C1	C1	C1	C1	C1
Resolution (Å)	3.6	2.9	3.0	3.0	3.1	3.1	3.1
Sharpening B-factor (Å^2)	42.5	50.0	43.6	46.0	43.7	45.4	41.6
**Refinement**							
**Model composition**							
Number of atoms	28743	29703	32469	31502	32402	29718	32361
Protein residues	3478	3592	3943	3823	3934	3594	3929
Nucleotides	53	53	54	54	54	54	54
Ligand	1 MG; 6 ZN	1 GTP; 1 MG; 8 ZN	2 MG; 1 PPI; 8 ZN	1 MG; 1 PPI; 6 ZN	1 MG; 1 PPI; 8 ZN	1 MG; 1 PPI; 8 ZN	1 MG; 1 PPI; 8 ZN
**Bonds RMSD**							
Bonds lengths (Å)	0.0032	0.0028	0.0120	0.0122	0.0118	0.0021	0.0121
Bonds angles (°)	0.55	0.54	0.54	0.55	0.49	0.49	0.57
**Validation**							
MolProbity score	1.74	1.57	1.70	1.58	1.53	1.46	1.77
Clash score	8.23	6.43	8.23	7.11	6.76	6.39	9.94
Rotamer outliers	0.00%	0.00%	0.00%	0.00%	0.00%	0.09%	0.00%
C-beta outliers	0.00%	0.00%	1.00%	1.00%	1.00%	0.00%	1.00%
**Ramachandran plot**							
Favored (%)	95.78%	96.67%	96.22%	96.85%	97.11%	97.44%	96.23%
Allowed (%)	4.10%	3.19%	3.70%	3.12%	2.84%	2.53%	3.74%
Outlier (%)	0.12%	0.14%	0.08%	0.03%	0.05%	0.03%	0.03%

Supplementary Table 5 | Cryo-EM data collection and structure determination of Long-RNA RNAPII EC with substrate ATP (Sample 4)

Map	State III (TL-open)	State V (TL-closed)
**PDB**	30EP	30ES
**EMDB**	EMD-57673	EMD-57676
**Data collection**		
Microscope	Titan Krios Gatan	
Detector	K3 with GIF Quantum	
Cs (mm)	2.7	2.7
Magnification	105k	105k
Pixel size (Å)	0.829	0.829
Electron dose (e-/Å2)	50	50
Defocus range (µm)	-0.5 to -2.5	-0.5 to -2.5
Micrograph Number	2000	2000
**Reconstruction**		
Software	cryoSPARC-4.5	
Particles refinement	59,360	66,728
Symmetry	C1	C1
Resolution (Å)	2.6	2.5
Sharpening B-factor (Å2)	45.8	47.4
**Refinement**		
**Model composition**		
Number of atoms	30347	30516
Protein residues	3558	3570
Nucleotides	98	102
Ligand	1 ATP; 2 MG; 8 ZN	1 ATP; 2 MG; 8 ZN
**Bonds RMSD**		
Bonds lengths (Å)	0.0237	0.0236
Bonds angles (°)	0.96	0.93
**Validation**		
MolProbity score	1.69	1.60
Clash score	8.09	7.43
Rotamer outliers	0.13%	0.16%
C-beta outliers	0.00%	0.00%
**Ramachandran plot**		
Favored (%)	96.32%	96.82%
Allowed (%)	3.54%	2.95%
Outlier (%)	0.14%	0.23%

Supplementary Table 6 | Summary of RNAPII EC elements with residue ranges and color codes

Domain & Motif	Subunits	Residue range	Color code (Default)
Module 1	Rpb1 (Major), Rpb2, Rpb4–7	Rpb1: 1–662, 809–1141, 1275–end; Rpb2: 1127–1224; Rpb4–7 (full-length)	Light blue
Module 2	Rpb1, Rpb2 (Major), Rpb3, Rpb9–12	Rpb1: 663–808, 1142–1274; Rpb2: 1–1126; Rpb3, Rpb9–12 (full-length)	Tan
Clamp (includes Clamp head and core)	Rpb1	Rpb1: 1–346, 1396–1436	Pale goldenrod
Lid	Rpb1	Rpb1: 250–260	Black
Funnel	Rpb1	Rpb1: 664–808	Orange or Orange red
Funnel tip	Rpb1	Rpb1: 706–708	Red
Cleft	Rpb1	Rpb1: 809–871, 1060–1141, 1276–1395	Yellow
Bridge helix (BH)	Rpb1	Rpb1: 809–848	Light green
Trigger loop (TL)	Rpb1	Rpb1: 1063–1107	Purple
Protrusion	Rpb2	Rpb2: 45–218, 406–465	Goldenrod
Lobe	Rpb2	Rpb2: 219–405	Sienna
Hybrid-binding (HB)	Rpb2	Rpb2: 751–852, 974–1127	Steel blue
Wall	Rpb2	Rpb2: 853–973	Navy
Lower jaw	Rpb5	Rpb5: 1–145	Slate blue
Upper jaw	Rpb1, Rpb9	Rpb1: 1142–1275; Rpb9: 1-39	Slate blue
Template strand DNA (tsDNA)	NA	NA	Cyan
Non-template strand DNA (ntsDNA)	NA	NA	Lime
RNA	NA	NA	Hot pink
ATP	NA	NA	Gold or hot pink
Mg^{2+} ions	NA	NA	Green

Supplementary Table 7 | Summary of Deposition Accession Codes and Permanent Hyperlinks

Sample / Dataset	State / Condition / Frame	EMDB Code & Hyperlink	PDB Code & Hyperlink
Sample 1: RNAPII EC-Apo	State-I	EMD-54704 [https://www.ebi.ac.uk/emdb/EMD-54704]	PDB 9SAY [http://doi.org/10.2210/pdb9SAY/pdb]
Sample 1: RNAPII EC-Apo	State-I-A	EMD-54705 [https://www.ebi.ac.uk/emdb/EMD-54705]	NA
Sample 1: RNAPII EC-Apo	State-I-B	EMD-54706 [https://www.ebi.ac.uk/emdb/EMD-54706]	NA
Sample 1: RNAPII EC-Apo	State-II	EMD-54707 [https://www.ebi.ac.uk/emdb/EMD-54707]	PDB 9SAZ [http://doi.org/10.2210/pdb9SAZ/pdb]
Sample 2: RNAPII EC + ATP (3D Variability)	Intermediate Model (Window=0, Frame 1)	EMD-54730 [https://www.ebi.ac.uk/emdb/EMD-54730]	PDB 9SBL [http://doi.org/10.2210/pdb9SBL/pdb]
Sample 2: RNAPII EC + ATP (3D Variability)	Intermediate Model (Window=0, Frame 2)	EMD-54731 [https://www.ebi.ac.uk/emdb/EMD-54731]	PDB 9SBM [http://doi.org/10.2210/pdb9SBM/pdb]
Sample 2: RNAPII EC + ATP (3D Variability)	Intermediate Model (Window=0, Frame 3)	EMD-54732 [https://www.ebi.ac.uk/emdb/EMD-54732]	PDB 9SBN [http://doi.org/10.2210/pdb9SBN/pdb]
Sample 2: RNAPII EC + ATP (3D Variability)	Intermediate Model (Window=0, Frame 4)	EMD-54733 [https://www.ebi.ac.uk/emdb/EMD-54733]	PDB 9SBO [http://doi.org/10.2210/pdb9SBO/pdb]

Sample 2: RNAPII EC + ATP (3D Variability)	Intermediate Model (Window=0, Frame 5)	EMD-54734 [https://www.ebi.ac.uk/emdb/EMD-54734]	PDB 9SBP [http://doi.org/10.2210/pdb9SBP/pdb]
Sample 2: RNAPII EC + ATP (3D Variability)	Intermediate Model (Window=0, Frame 6)	EMD-54735 [https://www.ebi.ac.uk/emdb/EMD-54735]	PDB 9SBQ [http://doi.org/10.2210/pdb9SBQ/pdb]
Sample 2: RNAPII EC + ATP (3D Variability)	Intermediate Model (Window=0, Frame 7)	EMD-54736 [https://www.ebi.ac.uk/emdb/EMD-54736]	PDB 9SBR [http://doi.org/10.2210/pdb9SBR/pdb]
Sample 2: RNAPII EC + ATP (3D Variability)	Intermediate Model (Window=0, Frame 8)	EMD-54737 [https://www.ebi.ac.uk/emdb/EMD-54737]	PDB 9SBS [http://doi.org/10.2210/pdb9SBS/pdb]
Sample 2: RNAPII EC + ATP (3D Variability)	Intermediate Model (Window=0, Frame 9)	EMD-54738 [https://www.ebi.ac.uk/emdb/EMD-54738]	PDB 9SBT [http://doi.org/10.2210/pdb9SBT/pdb]
Sample 2: RNAPII EC + ATP (3D Variability)	Intermediate Model (Window=0, Frame 10)	EMD-54739 [https://www.ebi.ac.uk/emdb/EMD-54739]	PDB 9SBU [http://doi.org/10.2210/pdb9SBU/pdb]
Sample 2: RNAPII EC + ATP (3D Variability)	Intermediate Model (Window=0, Frame 11)	EMD-54740 [https://www.ebi.ac.uk/emdb/EMD-54740]	PDB 9SBV [http://doi.org/10.2210/pdb9SBV/pdb]
Sample 2: RNAPII EC + ATP (3D Variability)	Intermediate Model (Window=0, Frame 12)	EMD-54741 [https://www.ebi.ac.uk/emdb/EMD-54741]	PDB 9SBW [http://doi.org/10.2210/pdb9SBW/pdb]
Sample 2: RNAPII EC + ATP (3D Variability)	Intermediate Model (Window=0, Frame 13)	EMD-54742 [https://www.ebi.ac.uk/emdb/EMD-54742]	PDB 9SBX [http://doi.org/10.2210/pdb9SBX/pdb]

Sample 2: RNAPII EC + ATP (3D Variability)	Intermediate Model (Window=0, Frame 14)	EMD-54743 [https://www.ebi.ac.uk/emdb/EMD-54743]	PDB 9SBY [http://doi.org/10.2210/pdb9SBY/pdb]
Sample 2: RNAPII EC + ATP (3D Variability)	Intermediate Model (Window=0, Frame 15)	EMD-54744 [https://www.ebi.ac.uk/emdb/EMD-54744]	PDB 9SBZ [http://doi.org/10.2210/pdb9SBZ/pdb]
Sample 2: RNAPII EC + ATP (3D Variability)	Intermediate Model (Window=0, Frame 16)	EMD-54745 [https://www.ebi.ac.uk/emdb/EMD-54745]	PDB 9SC0 [http://doi.org/10.2210/pdb9SC0/pdb]
Sample 2: RNAPII EC + ATP (3D Variability)	Intermediate Model (Window=0, Frame 17)	EMD-54746 [https://www.ebi.ac.uk/emdb/EMD-54746]	PDB 9SC1 [http://doi.org/10.2210/pdb9SC1/pdb]
Sample 2: RNAPII EC + ATP (3D Variability)	Intermediate Model (Window=0, Frame 18)	EMD-54747 [https://www.ebi.ac.uk/emdb/EMD-54747]	PDB 9SC2 [http://doi.org/10.2210/pdb9SC2/pdb]
Sample 2: RNAPII EC + ATP (3D Variability)	Intermediate Model (Window=0, Frame 19)	EMD-54748 [https://www.ebi.ac.uk/emdb/EMD-54748]	PDB 9SC3 [http://doi.org/10.2210/pdb9SC3/pdb]
Sample 2: RNAPII EC + ATP (3D Variability)	Intermediate Model (Window=0, Frame 20)	EMD-54749 [https://www.ebi.ac.uk/emdb/EMD-54749]	PDB 9SC4 [http://doi.org/10.2210/pdb9SC4/pdb]
Additional Maps (3D Classification)	3D Class A, Frame 1	EMD-54677 [https://www.ebi.ac.uk/emdb/EMD-54677]	NA
Additional Maps (3D Classification)	3D Class B, Frame 4	EMD-54682 [https://www.ebi.ac.uk/emdb/EMD-54682]	NA

Additional Maps (3D Classification)	3D Class C, Frame 7	EMD-54681 [https://www.ebi.ac.uk/emdb/EMD-54681]	NA
Additional Maps (3D Classification)	3D Class D, Frame 8	EMD-54680 [https://www.ebi.ac.uk/emdb/EMD-54680]	NA
Additional Maps (3D Classification)	3D Class E, Frame 9	EMD-54679 [https://www.ebi.ac.uk/emdb/EMD-54679]	NA
Additional Maps (3D Classification)	3D Class F, Frame 10	EMD-54683 [https://www.ebi.ac.uk/emdb/EMD-54683]	NA
Additional Maps (3D Classification)	3D Class G, Frame 13	EMD-54684 [https://www.ebi.ac.uk/emdb/EMD-54684]	NA
Additional Maps (3D Classification)	3D Class H, Frame 15	EMD-54685 [https://www.ebi.ac.uk/emdb/EMD-54685]	NA
Additional Maps (3D Classification)	3D Class I, Frame 17	EMD-54686 [https://www.ebi.ac.uk/emdb/EMD-54686]	NA
Additional Maps (3D Classification)	3D Class J, Frame 20	EMD-54687 [https://www.ebi.ac.uk/emdb/EMD-54687]	NA
Sample 3: RNAPII EC + NTP	State-II	EMD-54713 [https://www.ebi.ac.uk/emdb/EMD-54713]	PDB 9SB5 [http://doi.org/10.2210/pdb9SB5/pdb]
Sample 3: RNAPII EC + NTP	State-III	EMD-57322 [https://www.ebi.ac.uk/emdb/EMD-57322]	PDB 29RF [http://doi.org/10.2210/pdb29RF/pdb]
Sample 3: RNAPII EC + NTP	State-VI	EMD-54708 [https://www.ebi.ac.uk/emdb/EMD-54708]	PDB 9SB0 [http://doi.org/10.2210/pdb9SB0/pdb]

Sample 3: RNAPII EC + NTP	State-VII-A	EMD-54709 [https://www.ebi.ac.uk/emdb/EMD-54709]	PDB 9SB1 [http://doi.org/10.2210/pdb9SB1/pdb]
Sample 3: RNAPII EC + NTP	State-VII-B	EMD-54710 [https://www.ebi.ac.uk/emdb/EMD-54710]	PDB 9SB2 [http://doi.org/10.2210/pdb9SB2/pdb]
Sample 3: RNAPII EC + NTP	State-VII-C	EMD-54711 [https://www.ebi.ac.uk/emdb/EMD-54711]	PDB 9SB3 [http://doi.org/10.2210/pdb9SB3/pdb]
Sample 3: RNAPII EC + NTP	State-VII-D	EMD-54712 [https://www.ebi.ac.uk/emdb/EMD-54712]	PDB 9SB4 [http://doi.org/10.2210/pdb9SB4/pdb]
Sample 4: Long-RNA EC + ATP	State III (TL-open)	EMD-57673 [https://www.ebi.ac.uk/emdb/EMD-57673]	PDB 30EP [http://doi.org/10.2210/pdb30EP/pdb]
Sample 4: Long-RNA EC + ATP	State-V (TL-closed)	EMD-57676 [https://www.ebi.ac.uk/emdb/EMD-57676]	PDB 30ES [http://doi.org/10.2210/pdb30ES/pdb]

References

[1]. Sievers, F. *et al.* Fast, scalable generation of high-quality protein multiple sequence alignments using Clustal Omega. *Molecular Systems Biology* **7**, 539 (2011).

[2]. Zhu, C. *et al.* Transcription factors modulate RNA polymerase conformational equilibrium. *Nat Commun* **13**, 1546 (2022).

[3]. Kang, J. Y. *et al.* RNA Polymerase Accommodates a Pause RNA Hairpin by Global Conformational Rearrangements that Prolong Pausing. *Molecular Cell* **69**, 802-815.e5 (2018).
