# Supplementary material for: Structural dynamics of RNA polymerase II throughout the nucleotide addition cycle
Source: Nat Commun. 2026 Jul 8;17:8440. doi: 10.1038/s41467-026-75257-5 (PMC13478619; doi:10.1038/s41467-026-75257-5)
Supplement: Supplementary file 4 [file 41467_2026_75257_MOESM2_ESM.pdf]

Description of Additional Supplementary Files

Supplementary Movie 1: Summary of the nucleotide addition cycle and conformational dynamics of the RNAPII elongation complex

This movie summarizes the nucleotide addition cycle and associated conformational dynamics of the RNAPII EC. It integrates all experimentally determined structures to reconstruct a continuous trajectory of RNAPII during catalysis, spanning substrate binding, phosphodiester bond formation, and post-catalysis relaxation. The movie is organized into three hierarchical levels. First, a close-up view of the active site illustrates substrate incorporation, associated with TL folding and BH bending. Second, the view expands to the core region of the RNAPII EC, including the TL, BH, funnel, cleft, and RNA-DNA hybrid. Coordinated conformational rearrangements of these elements are shown alongside progression through the nucleotide addition cycle, highlighting their coupling to substrate binding and catalysis. Finally, the movie transitions to a global view of the EC, illustrating large-scale conformational changes across the entire complex, including clamp and nucleic acid scaffold dynamics across different functional states. Color coding of RNAPII domains and structural elements follows Table S6.

Supplementary Movie 2: Conformational dynamics of template loading in the RNAPII EC

This movie illustrates the conformational dynamics of template loading in the RNAPII EC, captured by the transition from state I to state II. The movie integrates cryo-EM density and corresponding atomic models from Dataset 2 to visualize the structural transition along this pathway. The i+1 nucleotide is highlighted in gold. The trajectory begins with the pre-template loading state (state I), followed by intermediate conformations (state I-A and state I-B), and progresses to the post-translocation state (state II). Color coding of RNAPII domains and structural elements follows Table S6.

Supplementary Movie 3: Conformational dynamics of TL during substrate incorporation in the RNAPII EC

This movie illustrates substrate incorporation coordinated with TL dynamics in the RNAPII EC. The animation integrates cryo-EM density and corresponding atomic models from Dataset 2 to visualize the conformational trajectory. The movie presents 20 intermediate frames derived from 3DVA, capturing a continuous transition from state I to state V. The trajectory begins with the pre-template loading state (state I), followed by the post-translocation state (state II) and the substrate-bound state (state III). This is followed by TL folding (state IV), culminating in the pre-catalysis state (state V). Color coding of RNAPII domains and structural elements follows Table S6.

Supplementary Movie 4: Conformational dynamics of the core region during substrate incorporation in the RNAPII EC

This movie illustrates substrate incorporation coordinated with conformational dynamics of the core region in the RNAPII EC. The animation integrates cryo-EM density from Dataset 2 to visualize the structural transition along this pathway. The core region includes the funnel, TL, cleft, hybrid binding (HB), BH, and RNA-DNA hybrid. The funnel tip is highlighted in red throughout the movie. The movie presents 20 intermediate frames derived from 3DVA, capturing the transition from state I to state V. During this process, coordinated conformational changes are observed within the core region, including funnel closure, BH bending, and TL folding. Color coding of RNAPII domains and structural elements follows Table S6.

Supplementary Movie 5: Global RNAPII EC tightening during substrate incorporation

This movie illustrates substrate incorporation coordinated with global conformational dynamics of the RNAPII EC. The animation integrates cryo-EM density from Dataset 2 to visualize the structural transition along this pathway. The movie begins with conformational changes within the core region and then transitions to a global view of the EC, highlighting the conformational transition and coordination from the core to the entire complex. The trajectory presents 20 intermediate frames derived from 3DVA, capturing the transition from state I to state V. At the global level, the tightening process is characterized by coordinated

conformational changes, including clamp closure and nucleic acid scaffold rearrangement across different functional states. Color coding of RNAPII domains and structural elements follows Table S6.

Supplementary Movie 6: Repositioning of the RNA-DNA hybrid and alignment of the RNA 3′-OH with the incoming substrate in the RNAPII EC

This movie illustrates substrate incorporation coordinated with repositioning of the RNA-DNA hybrid and alignment of the RNA 3′-OH with the incoming substrate in the RNAPII EC. The animation integrates cryo-EM density and corresponding atomic models from Dataset 2 to visualize the structural transition along this pathway. The trajectory presents 20 intermediate frames derived from 3DVA, capturing the transition from state I to state V. During this transition, the RNA-DNA hybrid undergoes progressive repositioning within the active site. In parallel, the 3′-OH of the RNA primer and the α-phosphate of the incoming substrate become increasingly aligned. Color coding of RNAPII structural elements follows Table S6.

Supplementary Movie 7: Phosphodiester bond formation in the RNAPII EC

This movie illustrates phosphodiester bond formation in the RNAPII EC. The animation integrates cryo-EM density and corresponding atomic models from Dataset 2 (state V, frame 20) and Dataset 3 (state VI) to visualize the transition between pre-catalysis and product states. A morphing approach is applied to generate a continuous transition from state V to state VI. During this transition, the α-phosphate of the incoming substrate forms a phosphodiester bond with the 3′-OH of the RNA primer. Concurrently, pyrophosphate is generated as a product of the reaction. The TL remains in a closed conformation in the product state. Color coding of RNAPII structural elements follows Table S6.

Supplementary Movie 8: TL unfolding during post-catalysis in the RNAPII EC

This movie illustrates TL unfolding during post-catalysis in the RNAPII EC. The animation integrates cryo-EM density and corresponding atomic models from Dataset 3 to visualize the structural transition along this pathway. A morphing approach is applied to generate a continuous transition across the product state (state VI) and subsequent intermediate states

(state VII-A to VII-D). These states are reconstructed from five cryo-EM density maps, capturing the progression of conformational changes following catalysis. During this transition, the TL undergoes progressive unfolding from a closed conformation in the product state to more open conformations in later states. Color coding of RNAPII structural elements follows Table S6.
